# High-Pressure Metal-Free Catalyzed One-Pot Two-Component Synthetic Approach for New 5-Arylazopyrazolo[3,4-*b*]Pyridine Derivatives

**DOI:** 10.3390/molecules27196369

**Published:** 2022-09-27

**Authors:** AbdElAziz A. Nayl, Hamada Mohamed Ibrahim, Kamal M. Dawood, Wael A. A. Arafa, Ahmed I. Abd-Elhamid, Ismail M. Ahmed, Mohamed A. Abdelgawad, Hazim M. Ali, Ibrahim Hotan Alsohaimi, Ashraf A. Aly, Stefan Bräse, Asmaa Kamal Mourad

**Affiliations:** 1 Department of Chemistry, College of Science, Jouf University, Sakaka 72341, Al Jouf, Saudi Arabia; 2 Chemistry Department, Faculty of Science, Fayoum University, Fayoum 63514, Faiyum, Egypt; 3 Chemistry Department, Faculty of Science, Cairo University, Giza 12613, Giza, Egypt; 4 Composites and Nanostructured Materials Research Department, Advanced Technology and New Materials Research Institute, City of Scientific Research and Technological Applications (SRTA-City), New Borg Al-Arab 21934, Alexandria, Egypt; 5 Department of Pharmaceutical Chemistry, College of Pharmacy, Jouf University, Sakaka 72341, Al Jouf, Saudi Arabia; 6 Chemistry Department, Faculty of Science, Organic Division, Minia University, El-Minia 61519, Menia, Egypt; 7 Institute of Organic Chemistry (IOC), Karlsruhe Institute of Technology (KIT), Fritz-Haber-Weg 6, 76133 Karlsruhe, Germany; 8 Institute of Biological and Chemical Systems—Functional Molecular Systems (IBCS-FMS), Director Hermann-von-Helmholtz-Platz 1, 76344 Eggenstein-Leopoldshafen, Germany

**Keywords:** 5-amino-2-phenyl-4*H*-pyrazol-3-one, arylhydrazonals, 5-arylazopyrazolo[3,4-*b*]pyridines, Q-tube

## Abstract

An appropriate and efficient Q-tube-assisted ammonium acetate-mediated protocol for the assembly of the hitherto unreported 5-arylazopyrazolo[3,4-*b*]pyridines was demonstrated. This methodology comprises the cyclocondensation reaction of 5-amino-2-phenyl-4*H*-pyrazol-3-one with an assortment of arylhydrazonals in an NH_4_OAc/AcOH buffer solution operating a Q-tube reactor. This versatile protocol exhibited several outstanding merits: easy work-up, mild conditions, scalability, broad substrate scope, safety (the Q-tube kit is simply for pressing and sealing), and a high atom economy. Consequently, performing such reactions under elevated pressures and utilizing the Q-tube reactor seemed preferable for achieving the required products in comparison to the conventional conditions. Diverse spectroscopic methods and X-ray single-crystal techniques were applied to confirm the proposed structure of the targeted compounds.

## 1. Introduction

**Figure 1:**
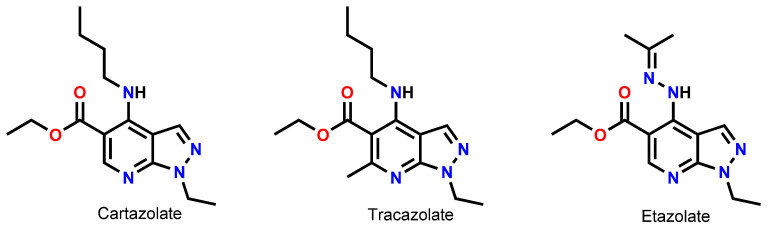
Structure of cartazolate, etazolate, and tracazolate.

Heterocycles are pivotal in diversified domains as they are considered the essential motif in industrial, agricultural, and biological fields. Intriguingly, heterocyclic compounds are prevalent in more than 85% of the pharmacologically active substances, and over 60% of FDA-approved medications possess, in their structure, nitrogen-based heterocycles [1]. Considering that they are contained in numerous therapeutic medicines marketed as anxiolytics, including cartazolate, etazolate, and tracazolate, they have been recognized as potent pharmaceutically important compounds (Figure 1) [2]. Furthermore, pyrazolo[3,4-*b*]pyridines are the key components of the cardiovascular therapeutic drug BAY 41-2272 [3] and the Glycogen Synthase Kinase 3 (GSK-3) inhibitor that is effective in the treatment of Alzheimer’s disease [4,5]. They are generally utilized to treat pulmonary hypertension as sGC stimulators [6,7]. Interestingly, pyrazolo[3,4-*b*]pyridines have a substantial inhibitory impact on diverse enzymes, such as Cyclin-Dependent Kinase (CDK) [8], Anaplastic Lymphoma Kinase (ALK) [9], nucleotide pyrophosphatase, and human recombinant alkaline phosphatase [10,11,12]. It is noteworthy that the pyrazolo[3,4-*b*]pyridine motif is a versatile system with various advantages. Antiproliferative, antiviral, antimicrobial [13], anticancer [14], anti-inflammatory [15], anti-HIV [16], antioxidant [17], antiallergic, and antiherpetic [18] biological activities are just a few of these. Due to the diverse applications of the heterocycles that comprise the pyrazolo[3,4-*b*]pyridine moiety, developing new protocols for their synthesis is challenging for pharmaceutical and organic chemists. The first Q-tube-mediated, high-pressure strategy for the preparation of an unparalleled series of thiazolo[4,5-*c*]pyridazines was recently published by our group [19]. Consequently, as part of our continued endeavors to develop efficient, environmentally friendly, and expedient protocols, a metal-free catalyst, high-pressure-assisted strategy for synthesizing pyrazolo[3,4-*b*]pyridines was explored in the present study. High-pressure chemistry (HPC) has been an unconventional, promising, practical, and full-potential approach to organic synthesis since 1981 [20]. Specifically, the use of the Q-tube is regarded as a pioneer in HPC. Notably, the Q-tube-mediated approach has numerous substantial advantages, including high reaction rates, cleaner reaction profiles, smaller reaction volumes, and quantitative conversions. According to the Arrhenius equation, utilizing the Q-tube approach might exponentially enhance the reaction rate as the boiling point is elevated.

Furthermore, increasing the pressure inside the Q-tube improves the probability of reactant collision, which accelerates the reaction rate, resulting in the minimization of competitive reagent decompositions and a smoother reaction profile [20]. In addition, the Q-tube is a cost-effective alternative to the costly microwave (MW) technique; it permits the reactions to be performed at a temperature higher than the solvent’s boiling point, even for MW-transparent solvents [21]. AlMarzouq et al. reported an intriguing assessment of numerous traditional and alternative heating procedures in 2016. The Q-tube strategy was recommended as the technique of preference for the cleanest, shortest, and most effective preparation of the heterocyclic compounds under study [22]. In other respects, the coupling Q-tube-assisted approach with the one-pot multicomponent reactions (MCRs) strategy is considered to be one of the most advantageous protocols for achieving step efficiency and atom economy [19,23,24,25,26]. In this study, the coupling of the Q-tube-mediated protocol with the two-component reaction (MCR) strategy can be investigated to provide universal access to a series of unreported 5-arylazopyrazolo[3,4-*b*]pyridines with superior reaction profiles and higher rates and approximately quantitative yields.

## 2. Materials and Methods

### 2.1. General

The measured melting points were determined by employing a Griffin melting point device, and the results were given incorrectly. The FT-IR spectra (KBr) were obtained utilizing the Jasco FT-IR-6300 spectrometer. The NMR spectra (^1^H: 600 MHz and ^13^C: 150 MHz) were obtained utilizing the Bruker DPX 600 super-conducting spectrometer, where the TMS was used as an internal reference and DMSO-*d6* or TFA-*d* as the solvent. The molecular weights of the synthesized compounds were recorded by employing both a high-resolution GC-MS (DFS) thermo-spectrometer [MS (EI) at 70.1 eV] and the magnetic sector mass analyzer [HRMS (EI)]. Thin layer chromatography (TLC) was used to monitor the progress of the reactions and to ensure product purity. All the reactions were carried out using a Q-tube gas purging kit (180 psi) from Q Labtech (Sigma-Aldrich, St. Louis, MO, USA), which included a catch bottle, PTFE-faced silicone septa, a borosilicate glass pressure tube (35.0 mL), a needle adapter, a Teflon sleeve, and a stainless steel adapter with a pressure gauge (300 psi). Microwave heating was carried out with a single-mode cavity Explorer Microwave synthesizer (CEM Corporation, Matthews, NC, USA), producing continuous irradiation and equipped with a simultaneous external air-cooling system. The Bruker X8 Prospector or Rigaku R-AXIS RAPID II diffractometer (Billerica, MA, USA) was used to record the X-ray crystallographic results. The arylhydrazonal derivatives (**2**) were synthesized by following up on the reported protocols [27].

### 2.2. General Protocol for the Synthesis of Compounds ***3a**–**v***

In the Q-tube reactor’s tube, a mixture of 5-amino-2-phenyl-4*H*-pyrazol-3-one (**1**) (5.0 mmol), arylhydrazonals **2** (5.0 mmol), glacial AcOH (10.0 mL), and AcONH_4_ (10.0 mmol) was introduced. The obtained mixture was then heated at 160 °C (oil bath) for 30 min, and the reaction progress was followed by employing TLC and GC/MS. After the reaction, the mixture was cooled to the ambient temperature. The obtained precipitate was filtered off, rinsed with EtOH (3 × 5.0 mL), and crystallized from the indicated solvent to yield pyrazolo[3,4-*b*]pyridin-3-ones **3a**–**v** as pure products.

1. **(*E*)-2,6-Diphenyl-5-(phenyldiazenyl)-2,7-dihydro-3*H*-pyrazolo[3,4-*b*]pyridin-3-one (3a).** Yellow crystals [EtOH/dioxane mixture (1:3)], yield: 1.90 g (98%), m.p. 255–256 °C; FT-IR 𝜈/cm^−1^: 3105 (N-H), 1656 (C=O), 1606 (C=N); ^1^H NMR (DMSO-*d6*, 600 MHz): *δ* 7.27–7.59 (m, 9H, Ar-H), 7.74–7.99 (m, 6H, Ar-H), 8.52 (s, 1H, C-H4), 12.34 ppm (brs, 1H, NH); ^13^C NMR (TFA-*d*, 100 MHz): *δ* 114.74, 12571, 126.29, 130.96, 131.47, 131.69, 132.27, 133.00, 133.23, 134.61, 135.32, 135.81, 136.00, 141.85, 146.39, 153.04, 159.07, 159.33 ppm; MS (EI) *m/z* (%): 392 (M^+^ + 1, 18.04), 391 (M^+^, 69.57), 390 (M^+^ − 1, 100); HRMS (EI) *m/z*: [M]^+^ calcd for C_24_H_17_N_5_O 391.1428, found 391.1428.
2. **(*E*)-5-[(4-Chlorophenyl)diazenyl]-2,6-diphenyl-2,7-dihydro-3*H*-pyrazolo[3,4-*b*]pyridin-3-one (3b).** Pale yellow crystals (dioxane), yield: 2.00 g (96%), m.p. 283–284 °C; FT-IR 𝜈/cm^−1^: 3106 (N-H), 1650 (C=O), 1620 (C=N); ^1^H NMR (TFA-*d*, 600 MHz): *δ* 8.19 (d, *J* = 8.4 Hz, 2H, Ar-H), 8.31–8.36 (m, 3H, Ar-H), 8.42–8.46 (m, 4H, Ar-H), 8.51–8.53 (m, 3H, Ar-H), 8.60 (d, *J* = 8.4 Hz, 2H, Ar-H), 10.18 ppm (s, 1H, C-H4), (exchanged proton with TFA, 1H, NH); ^13^C NMR (TFA-*d*, 150 MHz): *δ* 115.00, 126.97, 12765, 131.83, 132.07, 132.54, 132.92, 133.57, 133.80, 135.32, 136.28, 136.44, 142.90, 143.31, 147.49, 153.36, 159.33, 159.68 ppm; MS (EI) *m/z* (%): 427 (M^+^ + 2, 25.31), 426 (M^+^ + 1, 52.13), 425 (M^+^, 76.98), 424 (M^+^ − 1, 100); HRMS (EI) *m/z*: [M]^+^ calcd for C^24^H^16^ClN^5^O (M^+^) 425.1038, found 425.1038.
3. **(*E*)-5-[(4-Methoxyphenyl)diazinyl]-2,6-diphenyl-2,7-dihydro-3*H*-pyrazolo[3,4-*b*]pyridin-3-one (3c).** Yellow crystals [EtOH/dioxane mixture (1:2)], yield: 1.95 g (94%), m.p. 273–274 °C; FT-IR 𝜈/cm^−1^: 3116 (N-H), 1655 (C=O), 1602 (C=N); ^1^H NMR (DMSO-*d6*, 600 MHz): *δ* 3.82 (s, 3H, OC*H*_3_), 7.08 (d, *J* = 9.0 Hz, 2H, Ar-H), 7.27 (t, *J* = 7.2 Hz, 1H, Ar-H), 7.51 (t, *J* = 7.8 Hz, 2H, Ar-H), 7.54–7.56 (m, 3H, Ar-H), 7.71 (d, *J* = 7.8 Hz, 2H, Ar-H), 7.78 (d, *J* = 9.0 Hz, 2H, Ar-H), 7.98 (d, *J* = 7.2 Hz, 2H, Ar-H), 8.45 (s, 1H, C-H4), 12.30 ppm (brs, 1H, NH); ^13^C NMR (TFA-*d*, 100 MHz): *δ* 58.39 (O*C*H_3_), 113.77, 119.43, 126.38, 130.11, 130.75, 132.09, 132.41, 133.46, 133.56, 136.09, 136.46, 136.77, 137.03, 141.19, 146.86, 157.89, 160.54, 170.93 ppm; MS (EI) *m/z* (%): 422 (M^+^ + 1, 27.18), 421 (M^+^, 96.22), 420 (M^+^ − 1, 100); HRMS (EI) *m/z*: [M]^+^ calcd for C_25_H_19_N_5_O_2_ (M^+^) 421.1533, found 421.1533.
4. **(*E*)-5-[(4-Nitrophenyl)diazinyl]-2,6-diphenyl-2,7-dihydro-3*H*-pyrazolo[3,4-*b*]pyridin-3-one (3d).** Orange crystals [EtOH/DMF mixture (1:2)], yield: 2.05 g (95%), m.p. 299–300 °C; FT-IR 𝜈/cm^−1^: 3103 (N-H), 1659 (C=O), 1607 (C=N); ^1^H NMR (DMSO-*d6*, 600 MHz): *δ* 7.27 (t, *J* = 7.2 Hz, 1H, Ar-H), 7.51 (t, *J* = 7.8 Hz, 2H, Ar-H), 7.57–7.62 (m, 3H, Ar-H), 7.81 (d, *J* = 7.2 Hz, 2H, Ar-H), 7.87 (d, *J* = 8.4 Hz, 2H, Ar-H), 8.02 (d, *J* = 8.4 Hz, 2H, Ar-H), 8.37 (d, *J* = 7.8 Hz, 2H, Ar-H), 8.59 (s, 1H, C-H4), 12.33 ppm (brs, 1H, NH); ^13^C NMR (TFA-*d*, 100 MHz): *δ* 114.24, 126.56, 126.82, 127.42, 131.24, 131.71, 132.51, 133.28, 133.44, 134.96, 135.62, 136.17, 142.31, 147.04, 151.78, 157.88, 159.22, 159.79 ppm; MS (EI) *m/z* (%): 437 (M^+^ + 1, 28.19), 436 (M^+^, 92.89), 435 (M^+^ − 1, 100); HRMS (EI) *m/z*: [M]^+^ calcd for C_24_H_16_N_6_O_3_ (M^+^) 436.1278, found 436.1278.
5. **(*E*)-5-[(4-Chlorophenyl)diazinyl]-6-(naphthalen-2-yl)-2-phenyl-2,7-dihydro-3*H*-pyrazolo[3,4-*b*]pyridin-3-one (3e).** Pale orange crystals [EtOH/DMF mixture (1:3)], yield: 2.10 g (89%), m.p. 289–290 °C; FT-IR 𝜈/cm^−1^: 3111 (NH), 1653 (C=O), 1621 (C=N); ^1^H NMR (DMSO-*d6*, 600 MHz): *δ* 7.28 (t, *J* = 7.2 Hz, 1H, Ar-H), 7.53 (t, *J* = 7.8 Hz, 2H, Ar-H), 7.60–7.74 (m, 6H, Ar-H), 7.93–8.09 (m, 6H, Ar-H), 8.38 (s, 1H, Ar-H), 8.59 (s, 1H, C-H4), 12.57 ppm (brs, 1H, NH); ^13^C NMR (TFA-*d*, 150 MHz): *δ* 113.98, 126.49, 127.36, 128.32, 128.52, 130.40, 130.68, 131.57, 131.80, 132.25, 132.52, 132.65, 133.30, 134.59, 135.31, 135.81, 135.87, 138.06, 142.50, 142.56, 147.02, 152.71, 158.64, 159.05 ppm; MS (EI) *m/z* (%): 477 (M^+^ + 2, 34.11), 476 (M^+^ + 1, 52.35), 475 (M^+^, 100.00), 474 (M^+^ − 1, 71.98); HRMS (EI) *m/z*: [M]^+^ calcd for C_28_H_18_ClN_5_O (M^+^) 475.1194, found 475.1195.
6. **(*E*)-6-(Naphthalen-2-yl)-2-phenyl-5-(p-tolyldiazenyl)-2,7-dihydro-3*H*-pyrazolo[3,4-*b*]pyridin-3-one (3f).** Orange crystals [EtOH/DMF mixture (1:2)], yield: 2.00 g (88%), m.p. 284–285 °C; FT-IR 𝜈/cm^−1^: 3116 (NH), 1656 (C=O), 1605 (C=N); ^1^H NMR (DMSO-*d6*, 600 MHz): *δ* 2.36 (s, 3H, C*H*_3_), 7.29 (t, *J* = 7.8 Hz, 1H, Ar-H), 7.34 (d, *J* = 7.8 Hz, 2H, Ar-H), 7.53 (t, *J* = 7.8 Hz, 2H, Ar-H), 7.61–7.68 (m, 4H, Ar-H), 7.95 (d, *J* = 7.8 Hz, 1H, Ar-H), 8.01–8.09 (m, 5H, Ar-H), 8.38 (s, 1H, Ar-H), 8.55 (s, 1H, C-H4), 12.42 ppm (brs, 1H, NH); ^13^C NMR (TFA-*d*, 150 MHz): *δ* 22.68 (*C*H_3_), 115.00, 125.88, 126.30, 128.20, 128.40, 130.36, 130.70, 131.63, 131.91, 132.57, 132.64, 133.06, 133.10, 133.41, 134.23, 135.26, 136.15, 138.16, 140.78, 146.82, 149.10, 150.03, 159.45, 159.57 ppm; MS (EI) *m/z* (%): 456 (M^+^ + 1, 29.67), 455 (M^+^, 100.00), 454 (M^+^ − 1, 72.86); HRMS (EI) *m/z*: [M]^+^ calcd for C_29_H_21_N_5_O (M^+^) 455.1741, found 455.1740.
7. **(*E*)-6-(Naphthalen-2-yl)-5-[(4-nitrophenyl)diazinyl]-2-phenyl-2,7-dihydro-3*H*-pyrazolo[3,4-*b*]pyridin-3-one (3g).** Deep orange crystals [EtOH/DMF mixture (1:3)], yield: 2.05 g (85%), m.p. above 300 °C; FT-IR 𝜈/cm^−1^: 3108 (NH), 1654 (C=O), 1622 (C=N); ^1^H NMR (DMSO-*d6*, 600 MHz): *δ* 7.27 (t, *J* = 7.8 Hz, 1H, Ar-H), 7.52 (t, *J* = 7.8 Hz, 2H, Ar-H), 7.60–7.69 (m, 4H, Ar-H), 7.88 (d, *J* = 7.8 Hz, 1H, Ar-H), 7.94–8.06 (m, 4H, Ar-H), 8.10 (d, *J* = 7.8 Hz, 2H, Ar-H), 8.36 (d, *J* = 8.4 Hz, 1H, Ar-H), 8.42 (s, 1H, Ar-H), 8.65 (s, 1H, C-H4), 11.99 ppm (brs, 1H, NH); ^13^C NMR (TFA-*d*, 100 MHz): *δ* 114.13, 126.98, 127.16, 127.28, 127.92, 128.81, 129.04, 129.83, 130.82, 131.10, 131.97, 132.25, 133.02, 135.09, 135.74, 136.20, 136.51, 138.47, 142.67, 147.85, 152.21, 158.34, 159.40, 160.19 ppm; MS (EI) *m/*z (%): 487 (M^+^ + 1, 29.98), 486 (M^+^, 100.00), 485 (M^+^ − 1, 66.18); HRMS (EI) *m/z*: [M]^+^ calcd for C_28_H_18_N_6_O_3_ (M^+^) 486.1435, found 486.1435.
8. **(*E*)-5-[(4-Chlorophenyl)diazinyl]-6-(naphthalen-1-yl)-2-phenyl-2,7-dihydro-3*H*-pyrazolo[3,4-*b*]pyridin-3-one (3h).** Orange crystals [EtOH/DMF mixture (1:3)], yield: 2.10 g (88%), m.p. 277–278 °C; FT-IR 𝜈/cm^−1^: 3117 (NH), 1659 (C=O), 1611 (C=N); ^1^H NMR (DMSO-*d6*, 600 MHz): *δ* 7.25–7.27 (m, 3H, Ar-H), 7.42 (d, *J* = 8.4 Hz, 2H, Ar-H), 7.47 (t, *J* = 7.2 Hz, 1H, Ar-H), 7.51 (t, *J* = 7.8 Hz, 2H, Ar-H), 7.68–7.74 (m, 3H, Ar-H), 8.06–8.08 (m, 3H, Ar-H), 8.16 (d, *J* = 7.8 Hz, 2H, Ar-H), 8.63 (s, 1H, C-H4), 12.52 ppm (brs, 1H, NH); ^13^C NMR (TFA-*d*, 150 MHz): *δ* 115.23, 125.98, 126.37, 126.73, 126.83, 128.73, 129.46, 130.67, 131.12, 131.52, 132.07, 132.30, 133.12, 133.46, 134.20, 135.00, 135.98, 136.00, 142.12, 143.86, 146.54, 152.40, 158.81, 159.28 ppm; MS (EI) *m/z* (%): 477 (M^+^ + 2, 28.97), 476 (M^+^ + 1, 52.00), 475 (M^+^, 94.87), 474 (M^+^ − 1, 100.00); HRMS (EI) *m/z*: [M]^+^ calcd for C_28_H_18_ClN_5_O (M^+^) 475.1194, found 475.1194.
9. **(*E*)-6-(Naphthalen-1-yl)-5-[(4-nitrophenyl)diazinyl]-2-phenyl-2,7-dihydro-3*H*-pyrazolo[3,4-*b*]pyridin-3-one (3i).** Orange crystals [EtOH/DMF mixture (1:3)], yield: 2.00 g (83%), m.p. 275–276 °C; FT-IR 𝜈/cm^−1^: 3112 (NH), 1660 (C=O), 1618 (C=N); ^1^H NMR (DMSO-*d6*, 600 MHz): *δ* 7.26 (t, *J* = 7.2 Hz, 1H, Ar-H), 7.38 (d, *J* = 9.0 Hz, 2H, Ar-H), 7.47–7.51 (m, 3H, Ar-H), 7.58 (t, *J* = 7.8 Hz, 1H, Ar-H), 7.69–7.74 (m, 2H, Ar-H), 7.77 (d, *J* = 7.8 Hz, 1H, Ar-H), 8.05 (d, *J* = 7.8 Hz, 2H, Ar-H), 8.08 (d, *J* = 7.8 Hz, 1H, Ar-H), 7.15–7.18 (m, 3H, Ar-H), 8.68 (s, 1H, C-H4), 12.28 ppm (brs, 1H, NH); ^13^C NMR (TFA-*d*, 150 MHz): *δ* 115.07, 126.54, 12671, 126.90, 127.16, 127.23, 129.10, 129.19, 129.81, 131.00, 131.52, 132.27, 132.53, 132.60, 133.46, 133.90, 134.28, 136.37, 143.54, 147.26, 151.63, 157.86, 159.31, 160.26 ppm; MS (EI) *m/z* (%): 487 (M^+^ + 1, 26.02), 486 (M^+^, 100.00), 485 (M^+^ − 1, 97.84); HRMS (EI) *m/z*: [M]^+^ calcd for C_28_H_18_N_6_O_3_ (M^+^) 486.1435, found 486.1435.
10. **(*E*)-2-Phenyl-5-(phenyldiazenyl)-6-(thiophen-2-yl)-2,7-dihydro-3*H*-pyrazolo[3,4-*b*]pyridin-3-one (3j).** Pale yellow crystals [EtOH/dioxane mixture (1:2)], yield: 1.75 g (90%), m.p. 256–257 °C; FT-IR 𝜈/cm^−1^: 3112 (NH), 1656 (C=O), 1618 (C=N); ^1^H NMR (TFA-*d*, 600 MHz): *δ* 7.33 (t, *J* = 5.4 Hz, 1H, thiophene-H), 7.45–7.50 (m, 6H, Ar-H), 7.57 (d, *J* = 7.8 Hz, 2H, Ar-H), 7.92 (d, *J* = 7.8 Hz, 2H, Ar-H), 8.07 (d, *J* = 5.4 Hz, 1H, thiophene-H), 8.28 (d, *J* = 5.4 Hz, 1H, thiophene-H), 9.15 ppm (s, 1H, C-H4), (exchanged proton with TFA, 1H, NH); ^13^C NMR (TFA-*d*, 150 MHz): *δ* 111.76, 123.26, 124.85, 125.94, 126.48, 131.27, 131.71, 132.14, 132.75, 135.53, 135.90, 136.61, 139.61, 144.99, 146.45, 150.75, 153.13, 158.53 ppm; MS (EI) *m/z* (%): 398 (M^+^ + 1, 4.95), 397 (M^+^, 15.81), 396 (M^+^ − 1, 15.34); HRMS (EI) *m/z*: [M]^+^ calcd for C_22_H_15_N_5_OS (M^+^) 397.0992, found 397.0992.
11. **(*E*)-5-[(4-Chlorophenyl)diazinyl]-2-phenyl-6-(thiophen-2-yl)-2,7-dihydro-3*H*-pyrazolo[3,4-*b*]pyridin-3-one (3k).** Yellow crystals (dioxane), yield: 2.00 g (92%), m.p. 265–266 °C; FT-IR 𝜈/cm^−1^: 3114 (NH), 1659 (C=O), 1622 (C=N); ^1^H NMR (DMSO-*d6*, 600 MHz): *δ* 7.23 (t, *J* = 4.8 Hz, 1H, thiophene-H), 7.27 (t, *J* = 7.8 Hz, 1H, Ar-H), 7.50 (t, *J* = 7.8 Hz, 2H, Ar-H), 7.64 (d, *J* = 8.4 Hz, 2H, Ar-H), 7.89–7.92 (m, 5H, Ar-H), 8.07 (d, *J* = 4.8 Hz, 1H, thiophene-H), 8.30 (s, 1H, C-H4), 12.32 ppm (brs, 1H, NH); ^13^C NMR (TFA-*d*, 150 MHz): *δ* 112.24, 126.49, 128.28, 131.74, 131.92, 132.48, 132.69, 133.19, 133.55, 136.44, 136.99, 140.84, 143.02, 144.52, 147.59, 150.99, 153.29, 159.19 ppm; MS (EI) *m/z* (%): 433 (M^+^ + 2, 7.14), 432 (M^+^ + 1, 9.97), 431 (M^+^, 18.21), 430 (M^+^ − 1, 14.06); HRMS (EI) *m/z*: [M]^+^ calcd for C_22_H_14_ClN_5_OS (M^+^) 431.0602, found 431.0601.
12. **(*E*)-2-Phenyl-6-(thiophen-2-yl)-5-(p-tolyldiazenyl)-2,7-dihydro-3*H*-pyrazolo[3,4-*b*]pyridin-3-one (3l).** Yellow crystals (dioxane), yield: 2.05 g (88%), m.p. 263–264 °C; FT-IR 𝜈/cm^−^1: 3112 (NH), 1660 (C=O), 1618 (C=N); ^1^H NMR (DMSO-*d6*, 600 MHz): *δ* 2.44 (s, 3H, C*H*_3_), 7.28–7.31 (m, 2H, Ar-H), 7.47 (d, *J* = 7.8 Hz, 2H, Ar-H), 7.53 (t, *J* = 7.8 Hz, 2H, Ar-H), 7.91–7.94 (m, 4H, Ar-H), 7.96 (d, *J* = 4.8 Hz, 1H, thiophene-H), 8.15 (d, *J* = 4.8 Hz, 1H, thiophene-H), 8.39 (s, 1H, C-H4), 12.21 ppm (brs, 1H, NH); ^13^C NMR (TFA-*d*, 150 MHz): *δ* 22.40 (*C*H_3_), 111.96, 125.76, 126.63, 131.10, 131.41, 132.20, 132.69, 135.82, 136.66, 139.19, 145.17, 146.49, 148.78, 150.52, 150.78, 158.47 ppm; MS (EI) *m/z* (%): 412 (M^+^ + 1, 5.61), 411 (M^+^, 18.17), 410 (M^+^ − 1, 15.01); HRMS (EI) *m/z*: [M]^+^ calcd for C_23_H_17_N_5_OS (M^+^) 411.1148, found 411.1149.
13. **(*E*)-5-[(2,4-Difluorophenyl)diazinyl]-2-phenyl-6-(thiophen-2-yl)-2,7-dihydro-3*H*-pyrazolo- [3,4-*b*]pyridin-3-one (3m).** Orange crystals [EtOH/DMF mixture (1:2)], yield: 2.00 g (93%), m.p. 272–273 °C; FT-IR 𝜈/cm^−1^: 3110 (NH), 1650 (C=O), 1607 (C=N); ^1^H NMR (DMSO-*d6*, 600 MHz): *δ* 7.28–7.34 (m, 3H, Ar-H), 7.52 (t, *J* = 8.4 Hz, 2H, Ar-H), 7.62 (t, *J* = 8.4 Hz, 1H, Ar-H), 7.90–7.93 (m, 3H, Ar-H), 7.97 (d, *J* = 4.8 Hz, 1H, thiophene-H), 8.13 (d, *J* = 4.8 Hz, 1H, thiophene-H), 8.34 (s, 1H, C-H4), 12.45 ppm (brs, 1H, NH); ^13^C NMR (DMSO-*d6*, 150 MHz): *δ* (105.60, 105.77, 105.94) (t, ^2^*J*CF = 25.5 Hz), 109.60, (112.71, 112.86)] (dd, ^2^*J*CF = 22.5 Hz), 119.41, (120.15, 120.22) (d, ^3^*J*CF = 10.5 Hz), 121.14, 125.38, 128.27, 129.07, 132.60, 133.80, 137.01, 137.27, 139.30, 158.52, 159.03, 160.79, (163.28, 164.95 ppm) (d, ^1^*J*CF = 250.5 Hz); MS (EI) *m/z* (%): 434 (M^+^ + 1, 5.34), 433 (M^+^, 18.45), 432 (M^+^ − 1, 15.23); HRMS (EI) *m/z*: [M]^+^ calcd for C_22_H_13_F_2_N_5_OS (M^+^) 433.0803, found 433.0803.
14. **(*E*)-6-(4-Chlorophenyl)-5-[(4-chlorophenyl)diazinyl]-2-phenyl-2,7-dihydro-3*H*-pyrazolo- [3,4-*b*]pyridin-3-one (3n).** Yellow crystals (dioxane), yield: 2.25 g (99%), m.p. above 300 °C; FT-IR 𝜈/cm^−1^: 3114 (NH), 1651 (C=O), 1622 (C=N); ^1^H NMR (TFA-*d*, 600 MHz): *δ* 8.41 (d, *J* = 9.0 Hz, 2H, Ar-H), 8.53–8.57 (m, 3H, Ar-H), 8.62–8.66 (m, 4H, Ar-H), 8.74–8.77 (m, 4H, Ar-H), 10.42 ppm (s, 1H, C-H4), (exchanged proton with TFA, 1H, NH); ^13^C NMR (TFA-*d*, 150 MHz): *δ* 126.76, 127.47, 127.74, 129.78, 132.27, 132.35, 132.73, 133.54, 134.84, 135.10, 135.80, 142.65, 142.77, 143.87, 147.10, 152.85, 157.85, 159.04 ppm; MS (EI) *m/z* (%): 461 (M^+^ + 2, 66.28), 460 (M^+^ + 1, 92.93), 459 (M^+^, 100.00), 458 (M^+^ − 1, 98.02); HRMS (EI) *m/z*: [M]^+^ calcd for C_24_H_15_Cl_2_N_5_O (M^+^) 459.0648, found 459.0647.
15. **(*E*)-5-[(3-Bromophenyl)diazenyl]-6-(4-chlorophenyl)-2-phenyl-2,7-dihydro-3*H*-pyrazolo- [3,4-*b*]pyridin-3-one (3o).** Orange crystals (dioxane), yield: 2.40 g (97%), m.p. above 300 °C; FT-IR 𝜈/cm^−1^: 3111 (NH), 1650 (C=O), 1621 (C=N); ^1^H NMR (TFA-*d*, 600 MHz): *δ* 7.23 (t, *J* = 7.8 Hz, 1H, Ar-H), 7.42–7.48 (m, 4H, Ar-H), 7.53–7.57 (m, 4H, Ar-H), 7.66–7.70 (m, 3H, Ar-H), 7.74 (s, 1H, Ar-H), 9.28 ppm (s, 1H, C-H4), (exchanged proton with TFA, 1H, NH); ^13^C NMR (TFA-*d*, 150 MHz): *δ* 114.32, 125.57, 126.07, 126.26, 127.53, 129.37, 131.95, 132.36, 132.96, 133.08, 134.53, 134.74, 135.43, 138.11, 142.09, 143.59, 146.75, 155.01, 157.38, 158.89 ppm; MS (EI) *m/z* (%): 505 (M^+^ + 2, 89.91), 504 (M^+^ + 1, 100.00), 503 (M^+^, 61.42), 502 (M^+^ − 1, 55.03); HRMS (EI) *m/z*: [M]^+^ calcd for C_24_H_15_BrClN_5_O (M^+^) 503.0143, found 503.0144.
16. **(*E*)-6-(4-Chlorophenyl)-5-[(2-nitrophenyl)diazinyl]-2-phenyl-2,7-dihydro-3*H*-pyrazolo[3,4-*b*]pyridin-3-one (3p).** Orange crystals [EtOH/DMF mixture (1:3)], yield: 2.15 g (92%), m.p. 296–297 °C; FT-IR 𝜈/cm^−1^: 3106 (NH), 1662 (C=O), 1626 (C=N); ^1^H NMR (DMSO-*d6*, 600 MHz): *δ* 7.28 (t, *J* = 7.8 Hz, 1H, Ar-H), 7.50–7.52 (m, 3H, Ar-H), 7.61 (dd, *J* = 1.8, 8.4 Hz, 2H, Ar-H), 7.69 (t, *J* = 7.8 Hz, 1H, Ar-H), 7.79 (t, *J* = 7.8 Hz, 1H, Ar-H), 7.83 (d, *J* = 8.4 Hz, 2H, Ar-H), 7.99 (d, *J* = 8.4 Hz, 2H, Ar-H), 8.04 (d, *J* = 7.8 Hz, 1H, Ar-H), 8.37 (s, 1H, C-H4), 12.34 ppm (brs, 1H, NH); ^13^C NMR (DMSO-*d6*, 150 MHz): *δ* 118.12, 119.23, 122.20, 123.58, 124.93, 127.48, 128.51, 130.64, 132.28, 132.97, 133.77, 134.73, 137.23, 138.58, 140.62, 143.79, 146.80, 152.79, 158.36, 158.66 ppm; MS (EI) *m/z* (%): 472 (M^+^ + 2, 10.04), 471 (M^+^ + 1, 13.45), 470 (M^+^, 27.09), 469 (M^+^ − 1, 21.27); HRMS (EI) *m/z*: [M]^+^ calcd for C_24_H_15_ClN_6_O_3_ (M^+^) 470.0889, found 470.0887.
17. **(*E*)-6-(4-Fluorophenyl)-2-phenyl-5-(p-tolyldiazenyl)-2,7-dihydro-3*H*-pyrazolo[3,4-*b*] pyridin-3-one (3q).** Yellow crystals (dioxane), yield: 1.80 g (86%), m.p. above 300 °C; FT-IR 𝜈/cm^−1^: 3114 (NH), 1651 (C=O), 1622 (C=N); ^1^H NMR (TFA-*d*, 600 MHz): *δ* 2.43 (s, 3H, C*H*_3_), 7.35–7.40 (m, 4H, Ar-H), 7.57–8.62 (m, 3H, Ar-H), 7.69 (d, *J* = 8.4 Hz, 2H, Ar-H), 7.77 (d, *J* = 8.4 Hz, 2H, Ar-H), 7.90–7.92 (m, 2H, Ar-H), 9.46 (s, 1H, C-H4), (exchanged proton with TFA, 1H, NH); ^13^C NMR (TFA-*d*, 150 MHz): *δ* 22.29 (CH3), (118.91, 119.07) (d, ^2^*J*CF = 24.0 Hz), (125.34, 125.36) (d, ^4^JCF = 3.0 Hz), 126.87, 127.27, 128.26, 132.36, 132.42, (135.80, 135.87) (d, ^3^*J*CF = 10.5 Hz), 139.46, 140.78, 150.04, 150.28, 153.31, 153.45, 154.17, 155.53, 168.02, (168.18, 169.90 ppm) (d, ^1^*J*CF = 258.0 Hz); MS (EI) *m/z* (%): 424 (M^+^ + 1, 29.05), 423 (M^+^, 100.00), 422 (M^+^ − 1, 89.92); HRMS (EI) *m/z*: [M]^+^ calcd for C_25_H_18_FN_5_O (M^+^) 423.1490, found 423.1490.
18. **(*E*)-6-(4-Fluorophenyl)-5-[(4-nitrophenyl)diazinyl]-2-phenyl-2,7-dihydro-3*H*-pyrazolo[3,4-*b*]pyridin-3-one (3r).** Orange crystals [EtOH/DMF mixture (1:3)], yield: 2.05 g (91%), m.p. above 300 °C; FT-IR 𝜈/cm^−1^: 3112 (NH), 1662 (C=O), 1604 (C=N); ^1^H NMR (TFA-*d*, 600 MHz): *δ* 7.26 (t, J = 8.4 Hz, 2H, Ar-H), 7.45–7.49 (m, 3H, Ar-H), 7.56 (d, *J* = 7.8 Hz, 2H, Ar-H), 7.77–7.80 (m, 2H, Ar-H), 7.91 (d*, J* = 9.0 Hz, 2H, Ar-H), 8.29 (d, *J* = 9.0 Hz, 2H, Ar-H), 9.43 (s, 1H, C-H4), (exchanged proton with TFA, 1H, NH); ^13^C NMR (TFA-*d*, 150 MHz): *δ* 114.15, (118.86, 119.01) (d, ^2^*J*CF = 22.5 Hz), 126.35, 126.58, 127.16, 127.26, 132.32, 133.14, 134.83, 135.35, (135.81, 135.88) (d, ^3^*J*CF = 10.5 Hz), 142.02, 146.87, 151.65, 157.69, 158.36, 159.08, (167.97, 169.68 ppm) (d, ^1^*J*CF = 256.5 Hz); MS (EI) *m/z* (%): 455 (M^+^ + 1, 32.41), 454 (M^+^, 100.00), 422 (M^+^ − 1, 89.92); HRMS (EI) *m/z*: [M]^+^ calcd for C_24_H_15_FN_6_O_3_ (M^+^) 454.1184, found 454.1184.
19. **(*E*)-5-[(3-Bromophenyl)diazinyl]-6-(4-fluorophenyl)-2-phenyl-2,7-dihydro-3*H*-pyrazolo- [3,4-*b*]pyridin-3-one (3s).** Deep orange crystals [dioxane/DMF mixture (1:1)], yield: 2.10 g (87%), m.p. 285–286 °C; FT-IR 𝜈/cm^−1^: 3112 (NH), 1650 (C=O), 1622 (C=N); ^1^H NMR (DMSO-*d6*, 600 MHz): *δ* 7.26 (t, *J* = 7.2 Hz, 1H, Ar-H), 7.39 (t, *J* = 8.4 Hz, 2H, Ar-H), 7.48–7.51 (m, 3H, Ar-H), 7.66 (d, *J* = 7.2 Hz, 1H, Ar-H), 7.72 (d, *J* = 7.2 Hz, 1H, Ar-H), 7.77 (s, 1H, Ar-H), 7.82–7.96 (m, 4H, Ar-H), 8.46 (s, 1H, C-H4), 12.75 ppm (brs, 1H, NH); ^13^C NMR (TFA-*d*, 150 MHz): *δ* 114.13, (118.70, 118.85) (d, ^2^*J*CF = 22.5 Hz), 125.40, 125.96, 126.25, 127.25, 132.20, 132.68, 133.03, 134.72, 135.03, (135.70, 135.76) (d, ^3^*J*CF = 9.0 Hz), 138.04, 142.25, 146.41, 152.56, 154.87, 157.45, 158.94, (167.85, 169.56 ppm) (d, ^1^*J*CF = 256.5 Hz); MS (EI) *m/z* (%): 489 (M^+^ + 2, 72.19), 488 (M^+^ + 1, 83.04), 487 (M^+^, 71.43), 486 (M^+^ − 1, 62.35); H HRMS (EI) *m/z*: [M]^+^ calcd for C_24_H_15_BrFN_5_O (M^+^) 487.0439, found 487.0438.
20. **(*E*)-6-(4-Bromophenyl)-5-[(2,4-difluorophenyl)diazinyl]-2-phenyl-2,7-dihydro-3*H*-pyrazolo[3,4-*b*]pyridin-3-one (3t).** Orange crystals (DMF), yield: 2.40 g (97%), m.p. above 300 °C; FT-IR 𝜈/cm^−1^: 3112 (NH), 1655 (C=O), 1610 (C=N); ^1^H NMR (TFA-*d*, 600 MHz): *δ* 6.92 (td, *J* = 9.0, 4.5 Hz, 1H, Ar-H), 7.01 (td, *J* = 9.0, 4.5 Hz, 1H, Ar-H), 7.57–7.67 (m, 4H, Ar-H), 7.69 (d, *J* = 7.2 Hz, 2H, Ar-H), 7.73 (d, *J* = 8.4, Hz, 2H, Ar-H), 8.84 (d, *J* = 8.4, Hz, 2H, Ar-H), 9.49 (s, 1H, C-H4), (exchanged proton with TFA, 1H, NH); ^13^C NMR (TFA-*d*, 150 MHz): *δ* (107.36, 107.53, 107.69) (t, ^2^*J*CF = 25.5 Hz), 114.37, (114.50, 114.53) (d, ^4^*J*CF = 4.5 Hz), (122.15, 122.23) (d, ^3^*J*CF = 12.0 Hz), 126.34, 129.77, 131.56, 132.31, 133.14, 134.37, 134.91, 135.13, 135.18, (139.40, 139.43, 139.46) (t, ^4^*J*CF = 4.5 Hz), 142.87, 146.59, 157.32, 158.89, [(163.00, 163.09), (164.76, 164.85)] (dd, ^1^*J*CF = 13.5, 264.0 Hz), [(168.27, 168.35), (170.00, 170.08 ppm)] (dd, ^1^*J*CF = 12.0, 259.5 Hz); MS (EI) *m/z* (%): 507 (M^+^ + 2, 85.00), 506 (M^+^ + 1, 100.00), 505 (M^+^, 83.57), 504 (M^+^ − 1, 80.76); HRMS (EI) *m/z*: [M]^+^ calcd for C_24_H_14_BrF_2_N_5_O (M^+^) 505.0344, found 505.0351.
21. **(*E*)-6-(4-Chlorophenyl)-5-[(2-fluoro-5-nitrophenyl)diazinyl]-2-phenyl-2,7-dihydro-3*H*-pyrazolo[3,4-*b*]pyridin-3-one (3u).** Orange crystals (DMF), yield: 2.25 g (94%), m.p. above 300 °C; FT-IR 𝜈/cm^−1^: 3117 (NH), 1656 (C=O), 1622 (C=N); ^1^H NMR (TFA-*d*, 600 MHz): *δ* 6.91–7.73 (m, 9H, Ar-H), 9.66–9.98 (m, 3H, Ar-H), 10.61 (s, 1H, C-H4), (exchanged proton with TFA, 1H, NH); ^13^C NMR (TFA-*d*, 150 MHz): *δ* 114.22, 117.38, (121.76, 121.92) (d, ^2^*J*CF = 24.0 Hz), 127.13, 130.02, (131.71, 131.79) (d, ^3^*J*CF = 12.0 Hz), 132.66, 133.01, 133.92, 135.12, 135.47, 136.35, 142.43, (142.89, 142.96) (d, ^3^*J*CF = 10.5 Hz), 144.31, 147.20, 147.89, 159.05, 159.35, (166.23, 168.04 ppm) (d, ^1^*J*CF = 271.5 Hz); MS (EI) *m/z* (%): 490 (M^+^ + 2, 36.42), 489 (M^+^ + 1, 57.98), 488 (M^+^, 100.00), 487 (M^+^ − 1, 89.83); HRMS (EI) *m/z*: [M]^+^ calcd for C_24_H_14_ClFN_6_O_3_ (M^+^) 488.0794, found 488.0796.
22. **(*E*)-6-Methyl-2-phenyl-5-(phenyldiazenyl)-2,7-dihydro-3*H*-pyrazolo[3,4-*b*]pyridin-3-one (3v).** Pale yellow [EtOH/dioxane mixture (1:1)], yield: 1.35 g (83%), m.p. 283–284 °C; FT-IR 𝜈/cm^−1^: 3122 (NH), 1661 (C=O), 1620 (C=N); ^1^H NMR (DMSO-*d6*, 600 MHz): *δ* 2.89 (s, 3H, C*H*_3_), 7.18 (t, *J* = 7.8 Hz, 1H, Ar-H), 7.44 (t, *J* = 7.8 Hz, 2H, Ar-H), 7.49 (t, *J* = 7.8 Hz, 1H, Ar-H), 7.56 (t, *J* = 7.8 Hz, 2H, Ar-H), 7.84 (d, *J* = 7.8 Hz, 2H, Ar-H), 8.07 (d, *J* = 7.8 Hz, 2H, Ar-H), 8.42 (s, 1H, C-H4), 13.94 ppm (s, 1H, NH); ^13^C NMR (DMSO-*d6*, 150 MHz): *δ* 17.74 (*C*H_3_), 119.20, 122.75, 124.14, 124.89, 129.24, 129.77, 130.95, 135.71, 139.62, 152.71, 157.89, 160.03 ppm; MS (EI) *m/z* (%): 330 (M^+^ + 1, 23.91), 329 (M^+^, 100.00), 328 (M^+^ − 1, 8.23); HRMS (EI) *m/z*: [M]^+^ calcd for C_19_H_15_N_5_O (M^+^) 329.1271, found 329.1271.

## 3. Results and Discussion

Owing to the remarkable therapeutic usages of the pyrazolopyridines, it is worthwhile to assemble a unique family of arylazopyrazolo[3,4-*b*]pyridines (**3a**–**v**, Scheme 1, Figures S1–S44), utilizing a safer and greener approach. A series of arylhydrazonals **1a**–**v** was constructed following the reported protocols [27]. The reaction involving 5-amino-2-phenyl-4*H*-pyrazol-3-one (**1**) and 3-oxo-2-arylhydrazonopropanal (**2a**) was chosen as a template reaction to evaluate and study the optimal reaction conditions (Scheme 1 and Table 1).

**Scheme 2:**
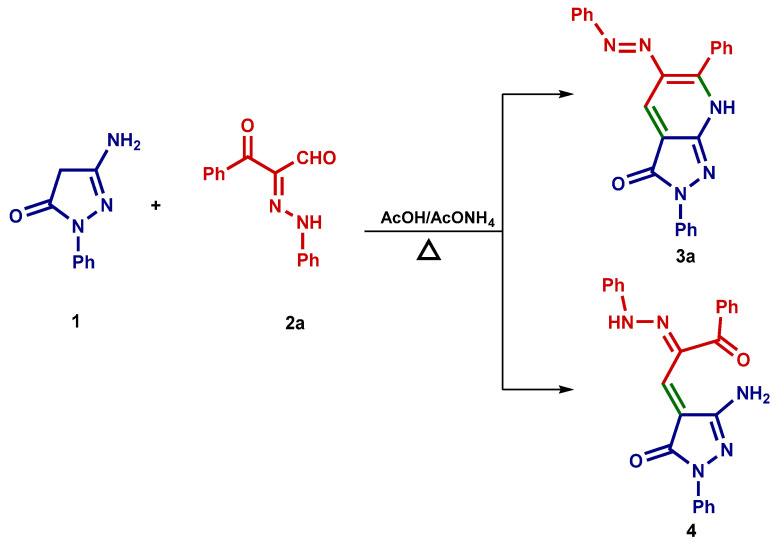
Reactions of 5-amino-2-phenyl-4*H*-pyrazol-3-one (**1**) with arylhydrazonal derivative **2a**.

At the outset, it was observed that refluxing a mixture of 5-amino-2-phenyl-4*H*-pyrazol-3-one (1, 5.0 mmol) and arylhydrazonal (**2a**, 5.0 mmol) in various solvents, including polar aprotic solvents (dioxane and CH_3_CN) and polar protic solvents (ethanol and propanol), comprising AcONH_4_ or anhydrous AcONa (10.0 mmol) under normal pressure for 12 h, did not produce any new products (Table 1, entries 1–4). They were interesting; utilizing DMF as a reaction solvent produced a new product in a 14% yield within 6 h, while the reaction yield did not increase with the increasing of the reaction duration (Table 1, entry 6). Furthermore, refluxing the selected reactants in acetic acid for 3 h yielded a product of a 45% yield when AcONH_4_ was used as an additive and a 30% yield when anhydrous AcONa was employed (Table 1, entries 6 and 7). Consequently, AcONH_4_ will be employed as an additive in the subsequent experiments. According to the results of several analyses, the newly obtained products in the cases above (Table 1, entries 5–7) are matched and elucidated to be 2,6-diphenyl-5-(phenyldiazenyl)-2,7-dihydro-3*H*-pyrazolo[3,4-*b*]pyridin-3-one (**3a**) and not the open-chain derivative **4** (Scheme 2). Among these analyses, the high-resolution mass and mass spectrometric analyses (See SI) of **3a** exhibited an exact mass of *m/z* 391.1428 and a mass of *m/z* 391, respectively, for the related molecular formula of C_24_H_17_N_5_O. The ^1^H NMR spectrum of **3a** in DMSO-*d*6 revealed a multiplet at δ 7.27–7.99 ppm due to 15 aromatic protons, a singlet signal for pyridine C-H4 at *δ* 8.25 ppm, and an abroad singlet assigned for the NH proton at *δ* 12.34 ppm. Furthermore, as anticipated, the ^13^C NMR spectrum of **3a** exhibited 18 signals with only one carbonyl signal.

The remarkable results motivated us to investigate the optimal parameters that impact the model reaction in a green and sustainable approach. Additionally, the investigation will be extended to demonstrate a comparative study between the microwave technique and the Q-tube methodology as an economical and affordable alternative to the costly MW. For comparison, we initially employed a MW (250 watts, 140 °C, 30 min) to perform the template reaction by mixing an equimolar amount (2.0 mmol) of compound **1** and **2a** in the presence of ammonium acetate (4.0 mmol)/acetic acid (5.0 mL) buffer solution. After the usual working up, compound **3a** was delivered in a 66% yield (Table 1, entry 8). Unfortunately, both the reaction rate and the obtained yield did not improve with the increasing of the reaction temperature and time. Interestingly, on employing the above-mentioned reaction utilizing the Q-tube pressure reactor (140 °C, 30 min), the targeted product **3a** was obtained with an 85% yield (Table 1, entry 9). It is worthwhile observing that doubling the amount of the substrates yielded **3a** with a comparatively similar efficiency and that prolonging the reaction interval would not enhance the reaction yield; therefore, employing the Q-tube provides a cleaner reaction profile and higher yields. Additionally, the Q-tube reactor was employed to perform such reactions safely under high pressure, avoiding the risk of unintentional explosions that could occur when a conventional sealed tube was utilized. After affirming the effectiveness and merits of the Q-tube and the AcOH/AcONH_4_ buffer in carrying out the desired reaction (Table 1, entry 9), the study was extended to investigate the impact of temperature on the reaction progress. The obtained results (Table 1, entries 10–12) indicated that the temperature considerably affects the reaction efficiency. For example, when the reaction was carried out at 150 °C, the target product was obtained at 92% (Table 1, entry 10); however, when the temperature was raised to 155, 160, and then 165 °C (Table 1, entries 11–13), compound **3a** was obtained at 96%, 98%, and 98%, respectively, indicating that 160 °C is the optimized temperature for such a conversion (Table 1, entry 12).

**Figure 2:**
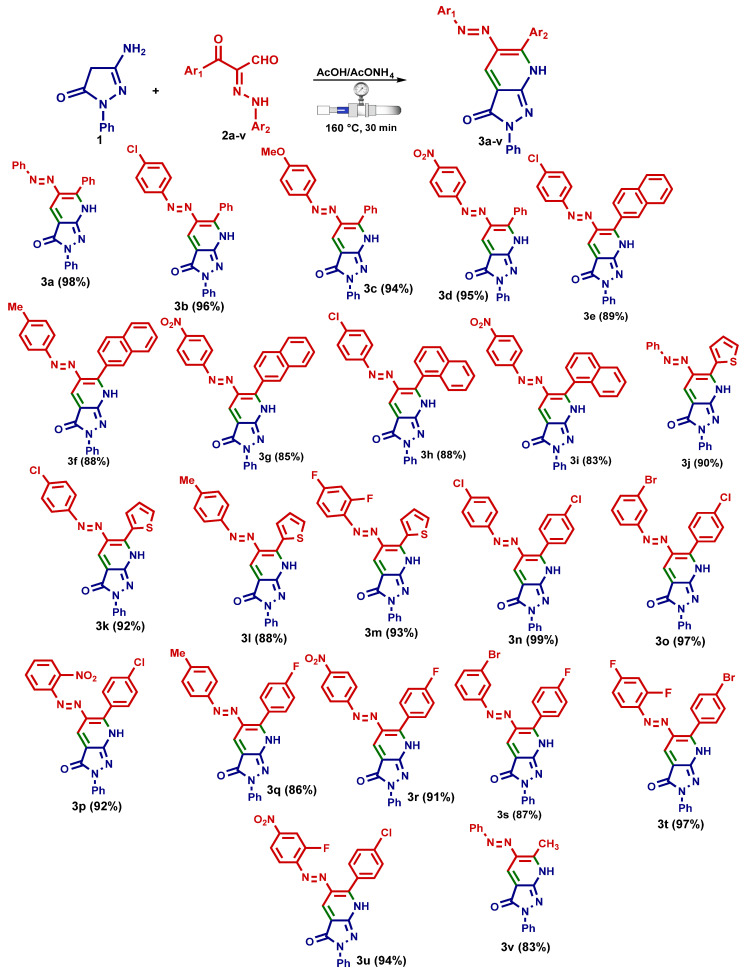
Cyclocondensation reactions between pyrazol-3-one **1** and arylhydrazonals **2** using Q-tube.

**Figure 3:**
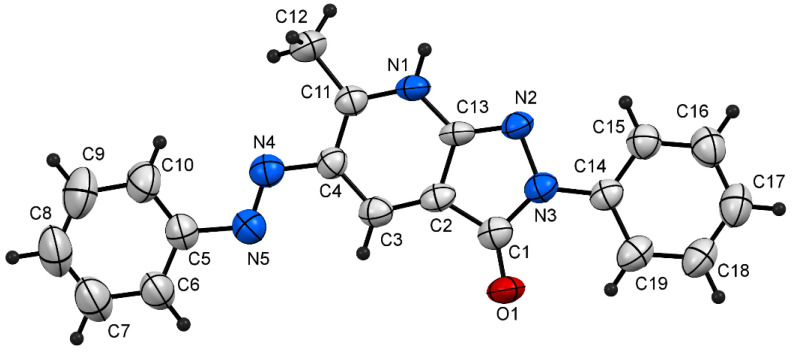
The X-ray crystallographic structure determined for **3v** [30].

Further investigations were carried out to study the potential, applicability, and limitations of the two NH_4_OAc-prompted successive condensation reactions (Figure 2) under the established optimal conditions (Table 1, entry 12). To achieve this target, a diversity of 3-oxo-arylhydrazonals **2a**–**v** was prepared and introduced in order to evaluate their reactions with 5-amino-2-phenyl-4*H*-pyrazol-3-one (**1**) under the specified optimal conditions (Table 1, entry 12). In general, the electronic properties of the aryl motifs attached to 3-oxo-arylhydrazonals (**2**) had a minimal influence on reaction efficacy [28,29]. The reaction was exceptionally adaptable to both electron-releasing motifs as well as the electron-accepting motifs. Gratifyingly, the naphthyl **2e**–**i** and thienyl **2j**–**m** derivatives had similar, successful, and smooth pathways in the case of 5-amino-2-phenyl-4*H*-pyrazol-3-one (**1**), yielding the condensed products in excellent yields (Figure 2). After several attempts, a suitable crystal for the X-ray single-crystallographic investigations was isolated as **3v** to confirm the initial results (Figure 3, Table 2). Moreover, the obtained single crystallographic data for the derivative **3v** (Figure 3, Table 2) confirmed the proposed structure and verified the regioselectivity of the reaction, yielding only the (*E*)-isomer of the 5-arylazopyrazolo[3,4-*b*]pyridine derivatives.

**Scheme 3:**
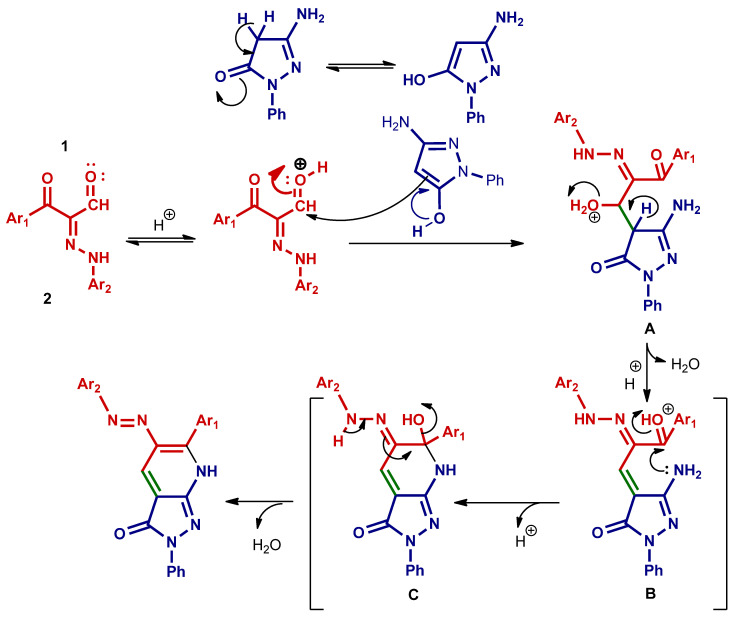
Plausible mechanistic route describes the formation of 5-arylazopyrazolo[3,4-*b*]pyridines **3a–v**.

Scheme 3 depicts a plausible mechanistic approach for synthesizing 5-arylazopyrazolo[3,4-*b*]pyridines **3a**–**v**. In the presence of acetic acid, the carbonyl groups became more polarized, and thus, their reactivity towards nucleophiles was enhanced. Firstly, the nucleophile generated from compound **1** underwent nucleophilic attack to the protonated carbonyl carbon of derivative **2**, to give the adduct **A**. Subsequently, the adduct (**A**) was easily converted to the non-isolable intermediary **B** by removing the good releasing group (OH_2_^+^). Secondly, the amino group underwent an intramolecular nucleophilic attack to the second protonated carbonyl carbon to obtain the intermediate **C**. Finally, the targeted products **3a**–**v** were formed via the exclusion of another water molecule (Scheme 3).

## 4. Conclusions

In conclusion, the present study represented an effective, practical, and adequate protocol for designing an assortment of the unprecedented 5-arylazopyrazolo[3,4-*b*]pyridines via the reaction between 5-amino-2-phenyl-4*H*-pyrazol-3-one and a variety of arylhydrazonals in good to excellent yields. The applied methodology involves the utilization of the Q-tube reactor as a safe and efficacious technique to perform the targeted reactions successfully under high pressure. The protocol possesses a variety of remarkable merits, including an excellent atom economy, a broad range of reactants, the avoidance of the employment of toxic solvents, and safe, straightforward purification and work-up methods. The X-ray structure analysis confirmed regioselectivity and yielded only the (*E*)-isomer of 5-arylazopyrazolo[3,4-*b*]pyridines. Consequently, we anticipate that this protocol will deliver a possibility for the implementation of such heterocycles in developing and designing new pharmaceuticals.

## Figures and Tables

**Scheme 1:**
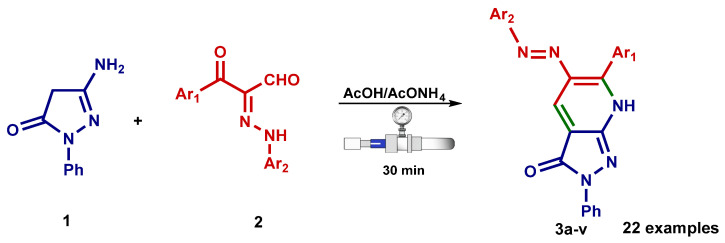
Synthesis of 5-arylazopyrazolo[3,4-*b*]pyridine derivatives **3a**–**v**.

**Table 1:**
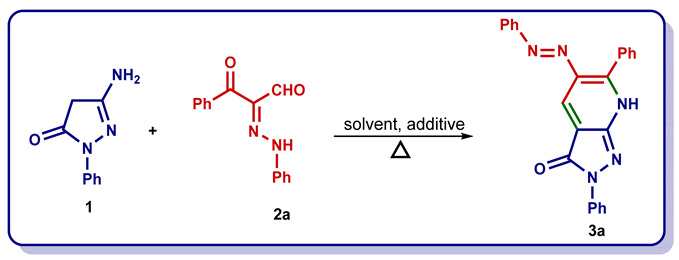
Optimization of the reaction of pyrazol-3-one **1** with phenylhydrazonal (**2a**).

Entry	Solvent	Additive (Equiv)	Temp. (°C)	Time	Yield (%)
1 ^a^	EtOH	NH_4_OAc or NaOAc	120	12 h	0
2 ^a^	CH_3_CN	NH_4_OAc or NaOAc	120	12 h	0
3 ^a^	1,4-dioxane	NH_4_OAc or NaOAc	140	12 h	0
4 ^a^	propanol	NH_4_OAc or NaOAc	130	12 h	0
5 ^a^	DMF	NH_4_OAc or NaOAc	140	6h	≈14
6 ^a^	AcOH	NH_4_OAc	140	6 h	45
7 ^a^	AcOH	NaOAc	140	6 h	30
8 ^b^	AcOH	NH_4_OAc	140	30 min	66
9 ^c^	AcOH	NH_4_OAc	140	30 min	85
10 ^c^	AcOH	NH_4_OAc	150	30 min	92
11 ^c^	AcOH	NH_4_OAc	155	30 min	96
12 ^c^	AcOH	NH_4_OAc	160	30 min	98
13 ^c^	AcOH	NH_4_OAc	165	30 min	98

^a^ Reaction conditions: independent mixture of 5-amino-2-phenyl-4*H*-pyrazol-3-one (**1**) (5.0 mmol) and arylhydrazonal **2a** (5.0 mmol) in AcOH (15.0 mL), comprising additive, was refluxed at the specified temp (°C) for the mentioned period. ^b^ Reaction conditions: independent mixture of **1** (2.0 mmol), arylhydrazonal **2a** (2.0 mmol), and NH_4_OAc (4.0 mmol) in AcOH (5.0 mL) was heated using the microwave irradiation (140 °C, 250 W) for 30 min. ^c^ Reaction conditions: independent mixture of **1** (5.0 mmol), arylhydrazonal **2a** (5.0 mmol), and NH_4_OAc (10.0 mmol) in AcOH (10.0 mL) was introduced into the Q-tube reactor and heated (oil bath) at the specified temp (°C) for 30 min.

**Table 2:** Some selected bond lengths and angles for compound **3c**.

Bond	Bond length (Å)	Bond	Bond Angle (^o^)
C5-C10	1.380 (9)	C7-C8-C9	119.5 (6)
C5-N5	1.444 (6)	C6-C5-C10	120.5 (5)
N4-C4	1.409 (6)	C3-C4-C11	121.0 (4)
N4-N5	1.258 (6)	N5-N4-C4	114.7 (5)
C3-C4	1.404 (8)	N4-N5-C5	112.3 (5)
N1-C11	1.353 (6)	C11-N1-C13	122.2 (4)
N2-N3	1.424 (6)	C2-C3-C4	118.7 (5)
C1-O1	1.238 (7)	N3-N2-C14	116.6 (4)
N3-C14	1.412 (6)	O1-C1-C2	131.9 (4)

## Data Availability

The data are contained within the article.

## Supplementary Materials

The following supporting information can be downloaded at: https://www.mdpi.com/article/10.3390/molecules27196369/s1. Figures S1–S44: Copies of NMR spectra for the reported compounds (PDF); the crystallographic data for compound **3v** (PDF, CIF).
