# Supplementary material for: High-Pressure Metal-Free Catalyzed One-Pot Two-Component Synthetic Approach for New 5-Arylazopyrazolo[3,4-b]Pyridine Derivatives
Source: Molecules. 2022 Sep 27;27(19):6369. doi: 10.3390/molecules27196369 (PMC9572034; doi:10.3390/molecules27196369)
Supplement: Supplementary file 1 [file molecules-27-06369-s001.zip › molecules-1923812-supplementary.pdf]

Supporting Information

For

High Pressure Metal-Free Catalyzed One-pot Two-component Synthetic Approach for Novel 5-Arylazopyrazolo[3,4-*b*]-Pyridine Derivatives

AbdElAziz A. Nayl[1,*], Hamada Mohamed Ibrahim [2, *], Kamal M. Dawood [3], Wael A. A. Arafa[1], Ahmed I. Abd-Elhamid[4], Ismail M. Ahmed[1], Mohamed A. Abdelgawad [5], Hazim M.Ali[1], Ibrahim Hotan Alsohaimi[1], Ashraf A. Aly [6], Stefan Bräse [7,8,*], Asmaa Kamal Mourad[2]

[1] Department of Chemistry, College of Science, Jouf University, Sakaka, Aljouf 72341, Saudi Arabia.
[2] Chemistry Department, Faculty of Science, Fayoum University, P.O. Box 63514, Fayoum, Egypt.
[3] Chemistry Department, Faculty of Science, Cairo University, Giza 12613, Egypt
[4] Composites and Nanostructured Materials Research Department, Advanced Technology and New Ma-terials Research Institute, City of Scientific Research and Technological Applications (SRTA-City), New Borg Al-Arab, Alexandria 21934, Egypt
[5] Department of Pharmaceutical Chemistry, College of Pharmacy, Jouf University, Sakaka, Al Jouf 72341, Saudi Arabia
[6] Chemistry Department, Faculty of Science, Organic Division, Minia University, El-Minia 61519, Egypt;
[7] Institute of Organic Chemistry (IOC), Karlsruhe Institute of Technology (KIT), Fritz-Haber-Weg 6, 76133 Karlsruhe, Germany
[8] Institute of Biological and Chemical Systems – Functional Molecular Systems (IBCS-FMS), Director Hermann-von-Helmholtz-Platz 1, D-76344 Eggenstein-Leopoldshafen, Germany

* Correspondence: aanayel@ju.edu.sa (A.A.N.); hmi00@fayoum.edu.eg (H.M.I.); stefan.braese@kit.edu (S.B.)

Contents

1- **Copies of ^{1}H, ^{13}C NMR spectra for the synthesized compounds.** **S2**

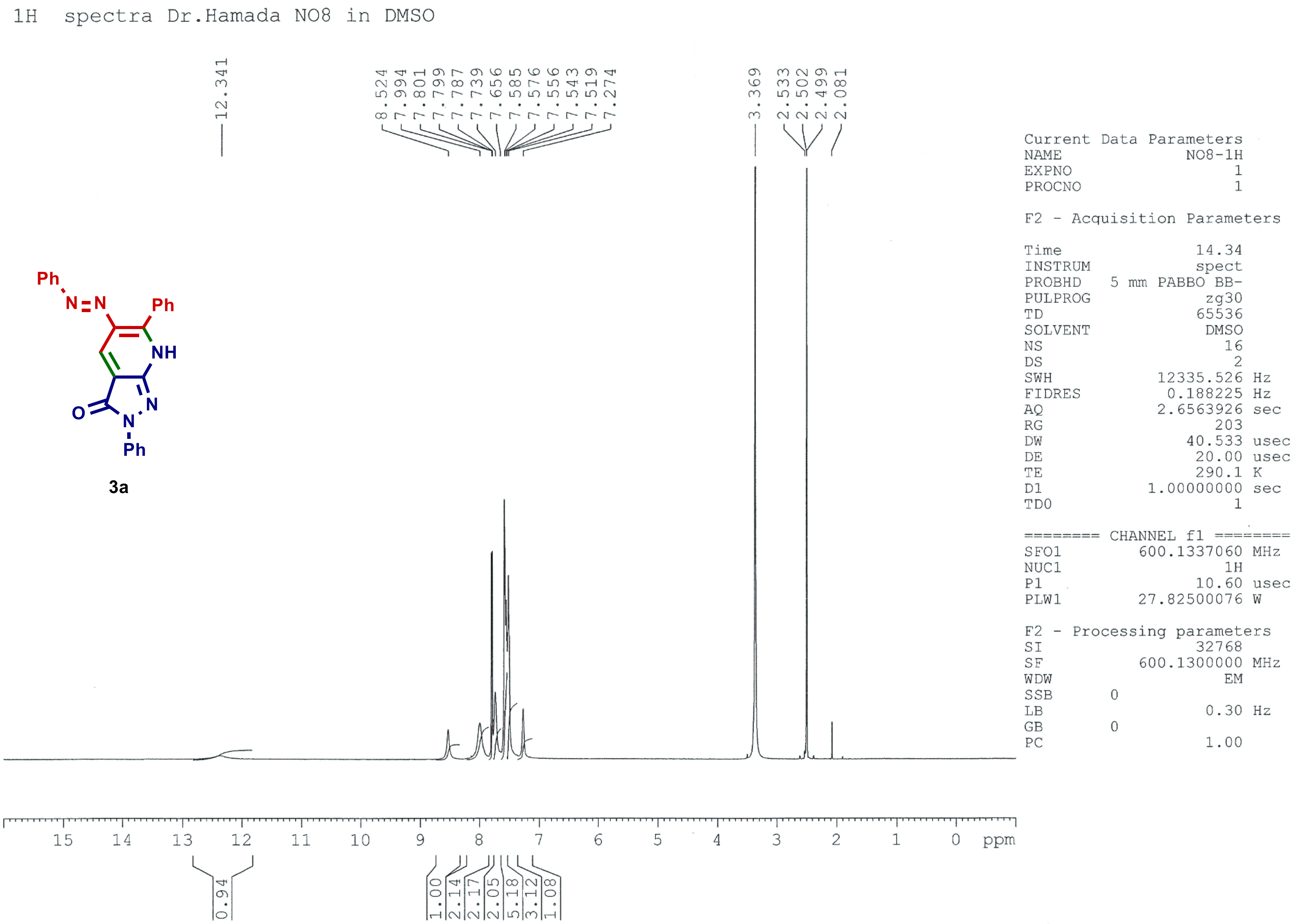

Figure S1. ^{1}H NMR Spectrum (DMSO-d_6, 600 MHz) for compound **3a**.

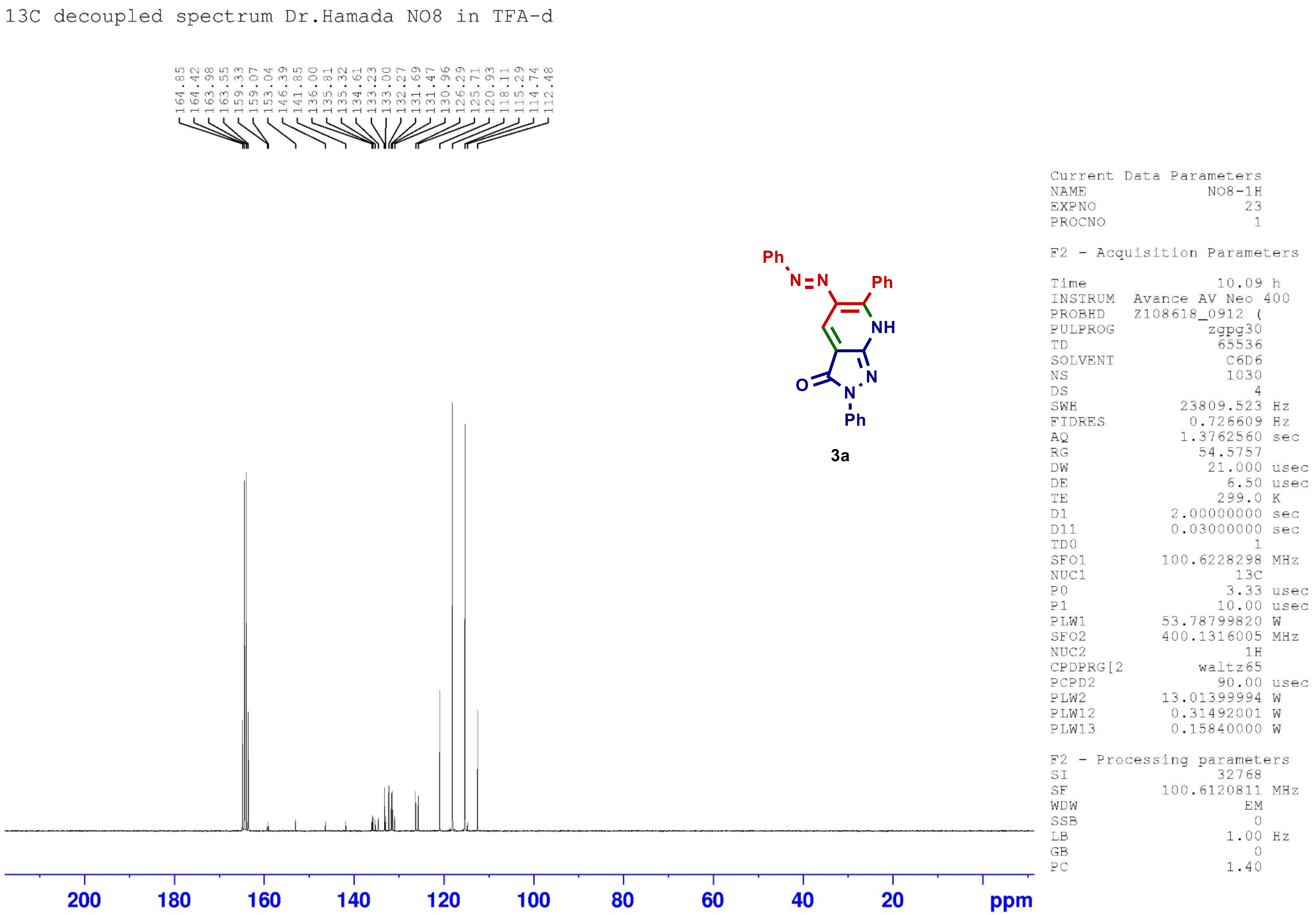

Figure S2. ^{13}C NMR Spectrum (TFA-*d*, 150 MHz) for compound **3a**.

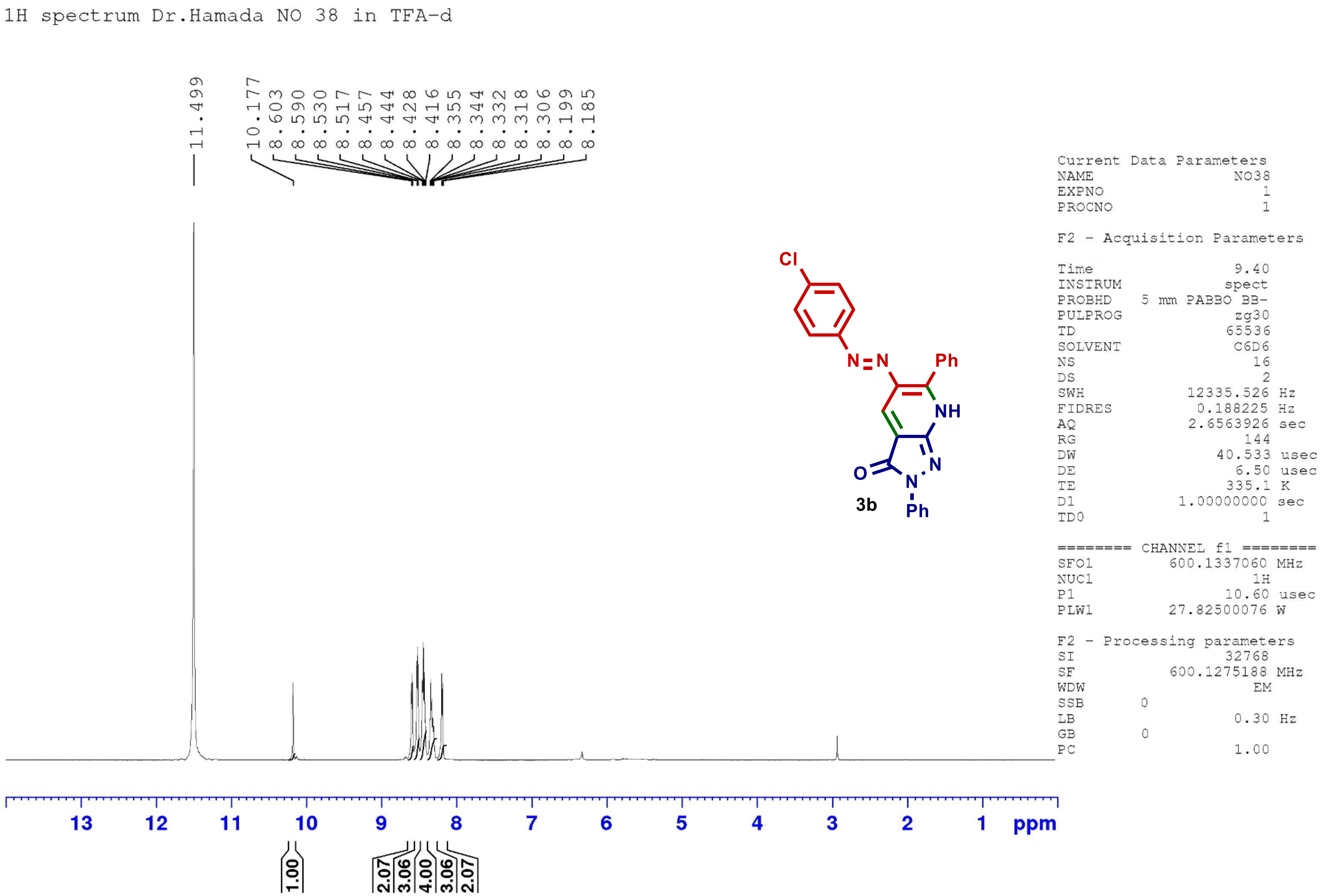

Figure S3. ^{1}H NMR Spectrum (TFA-*d*, 600 MHz) for compound **3b**.

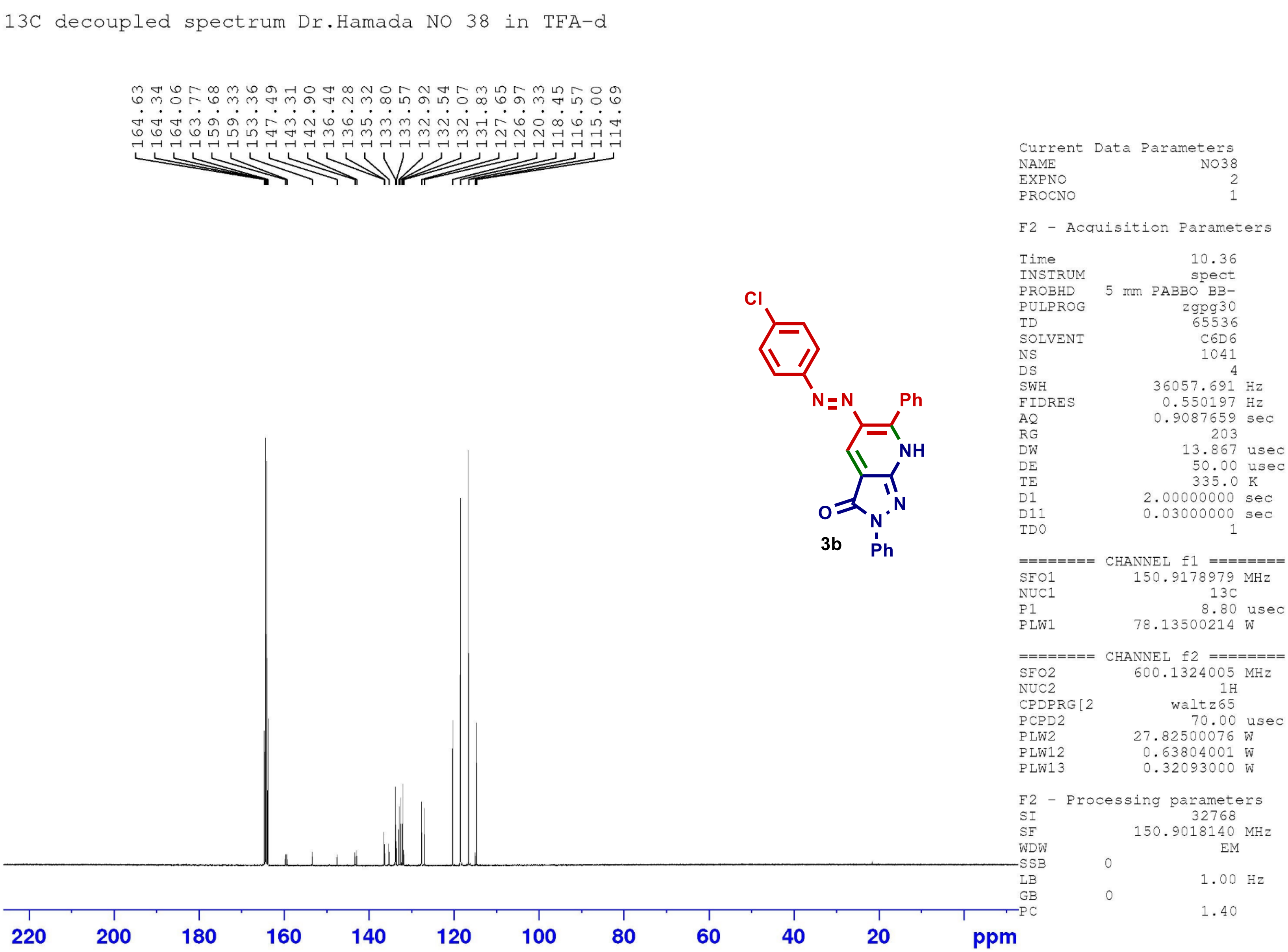

Figure S4. ^{13}C NMR Spectrum (TFA-*d*, 150 MHz) for compound **3b**.

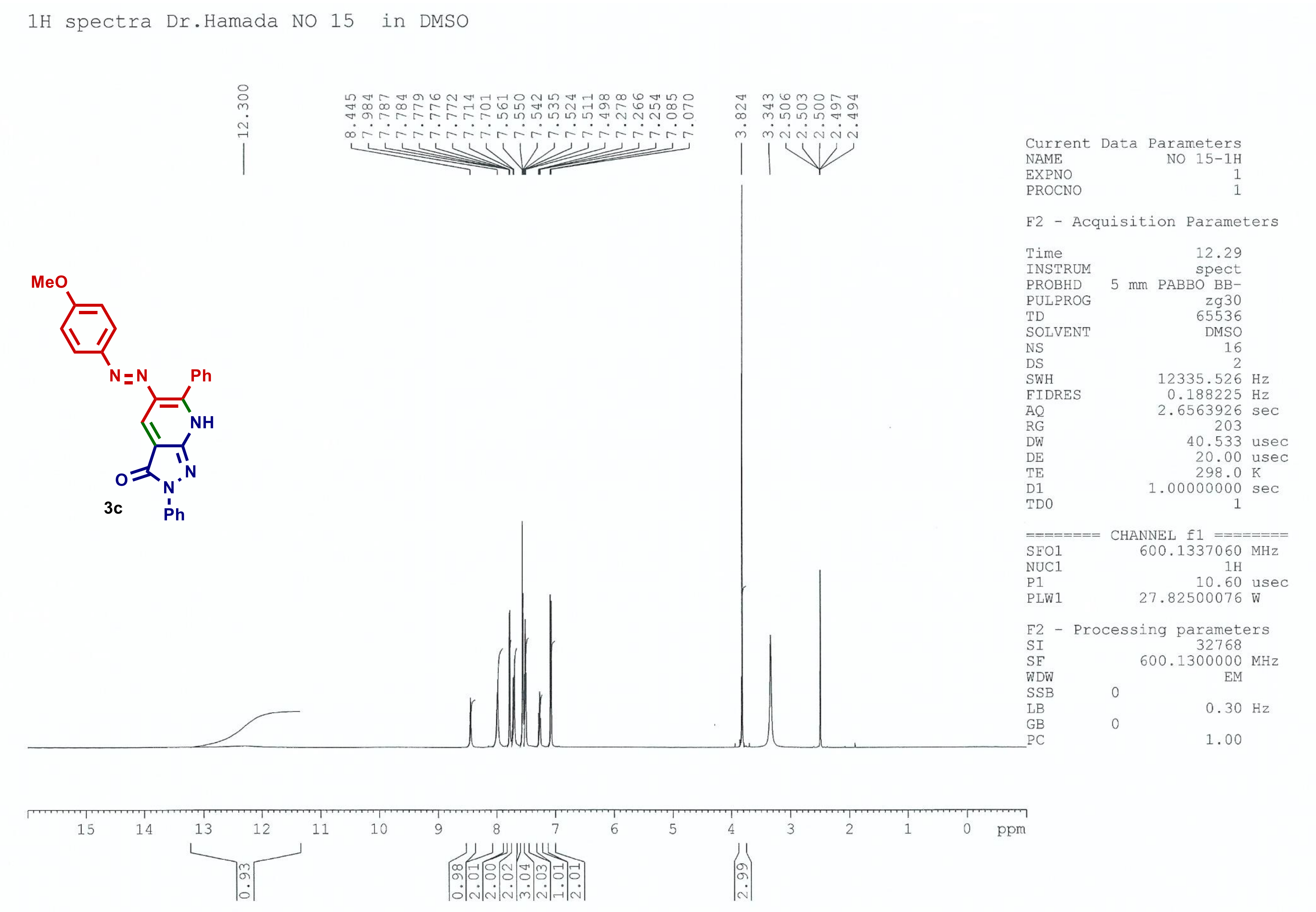

Figure S5. ^{1}H NMR Spectrum (DMSO-d_6, 600 MHz) for compound **3c**.

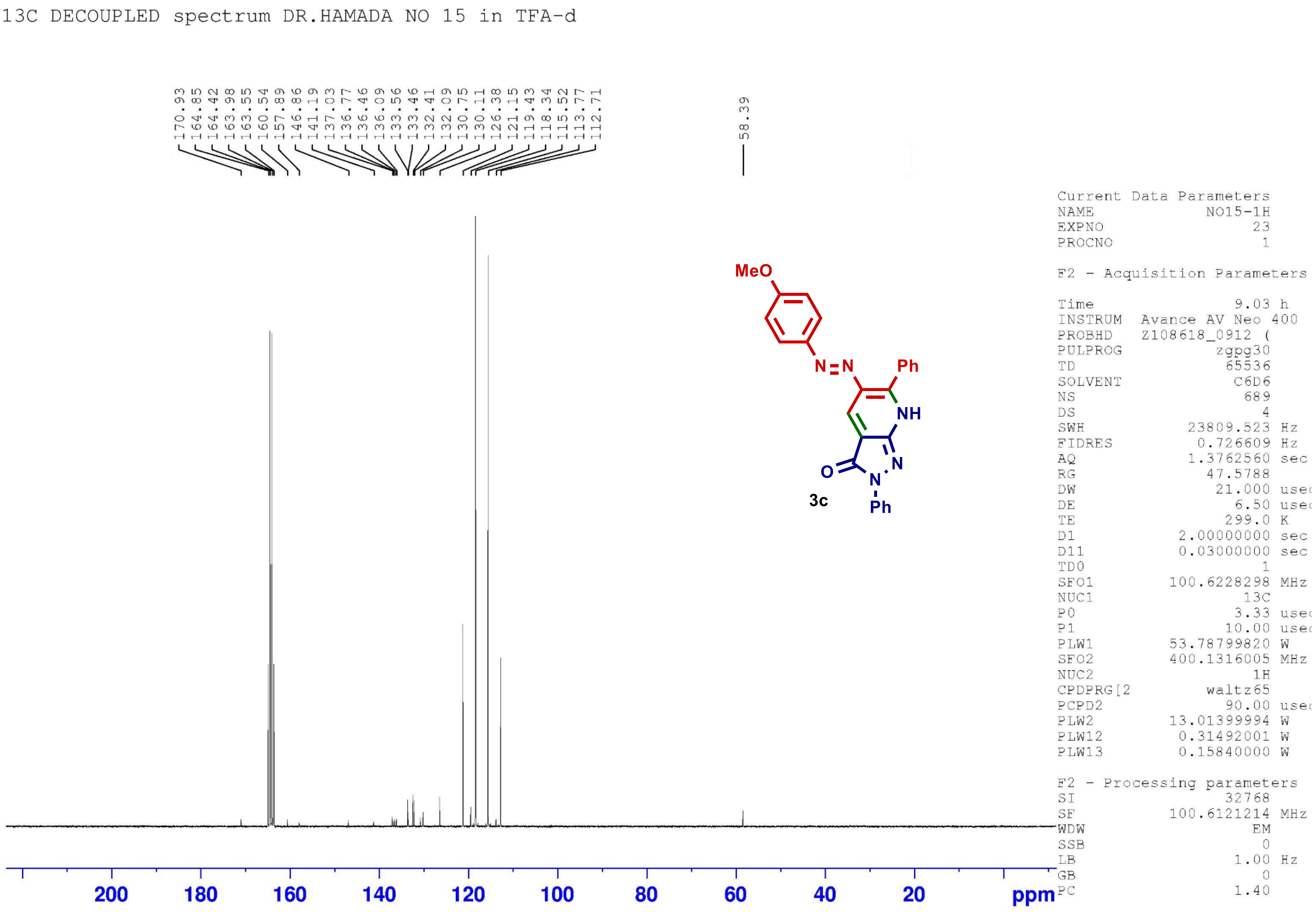

Figure S6. .^{13}C NMR Spectrum (TFA-*d*, 150 MHz) for compound **3c**.

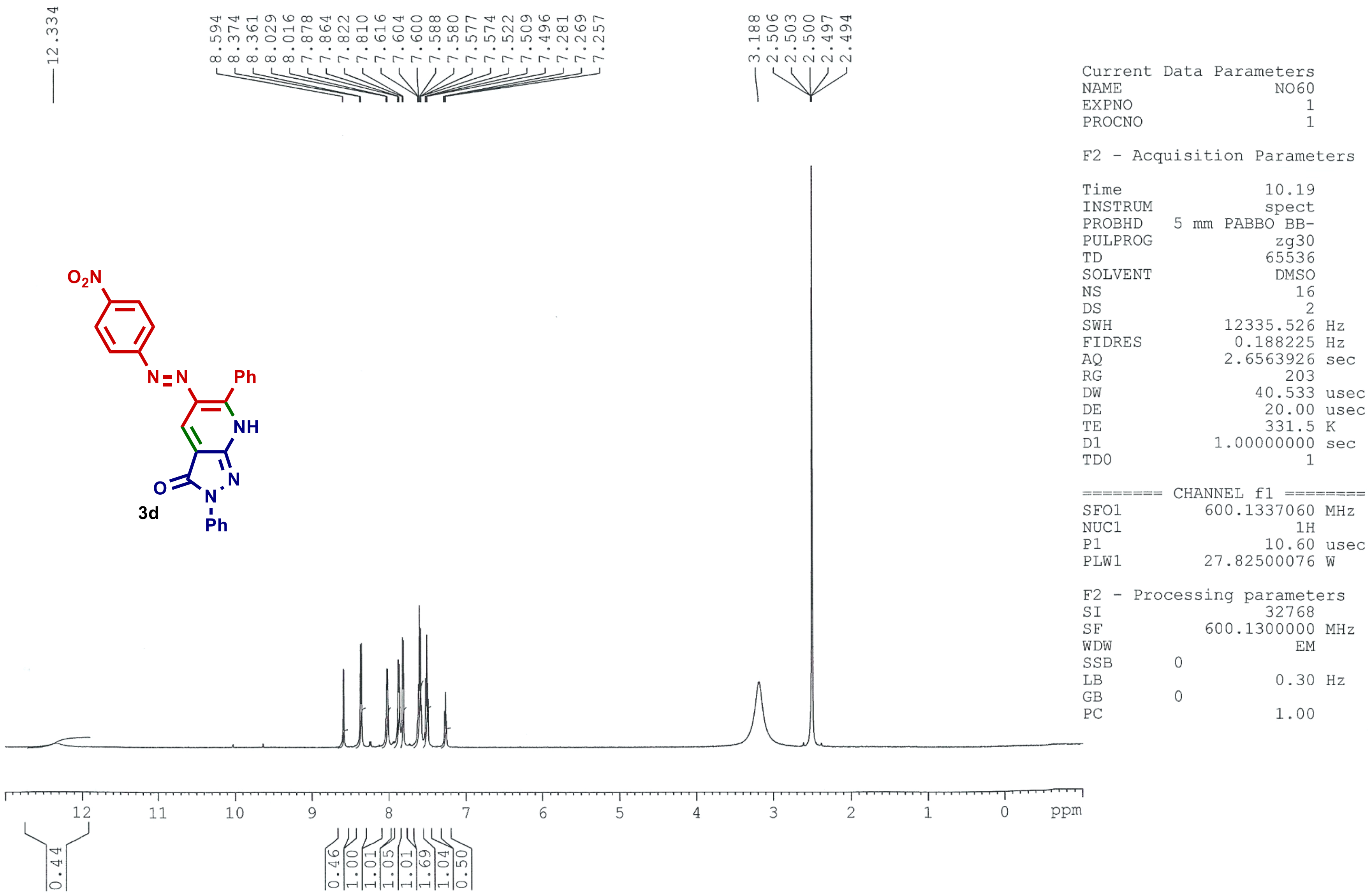

Figure S7. ^{1}H NMR Spectrum (DMSO-d_6, 600 MHz) for compound **3d**

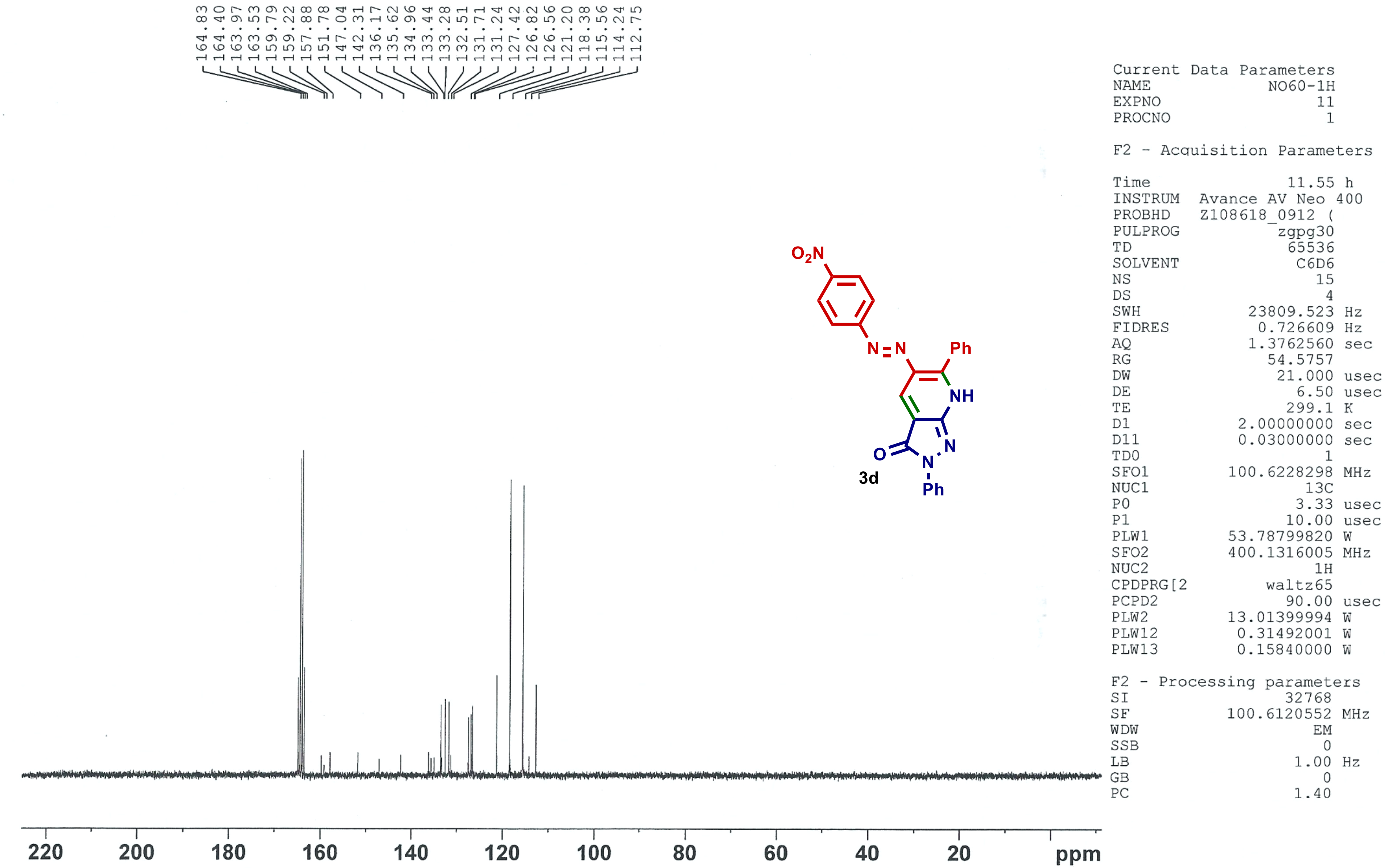

Figure S8. ^{13}C NMR Spectrum (TFA-*d*, 150 MHz) for compound **3d.**

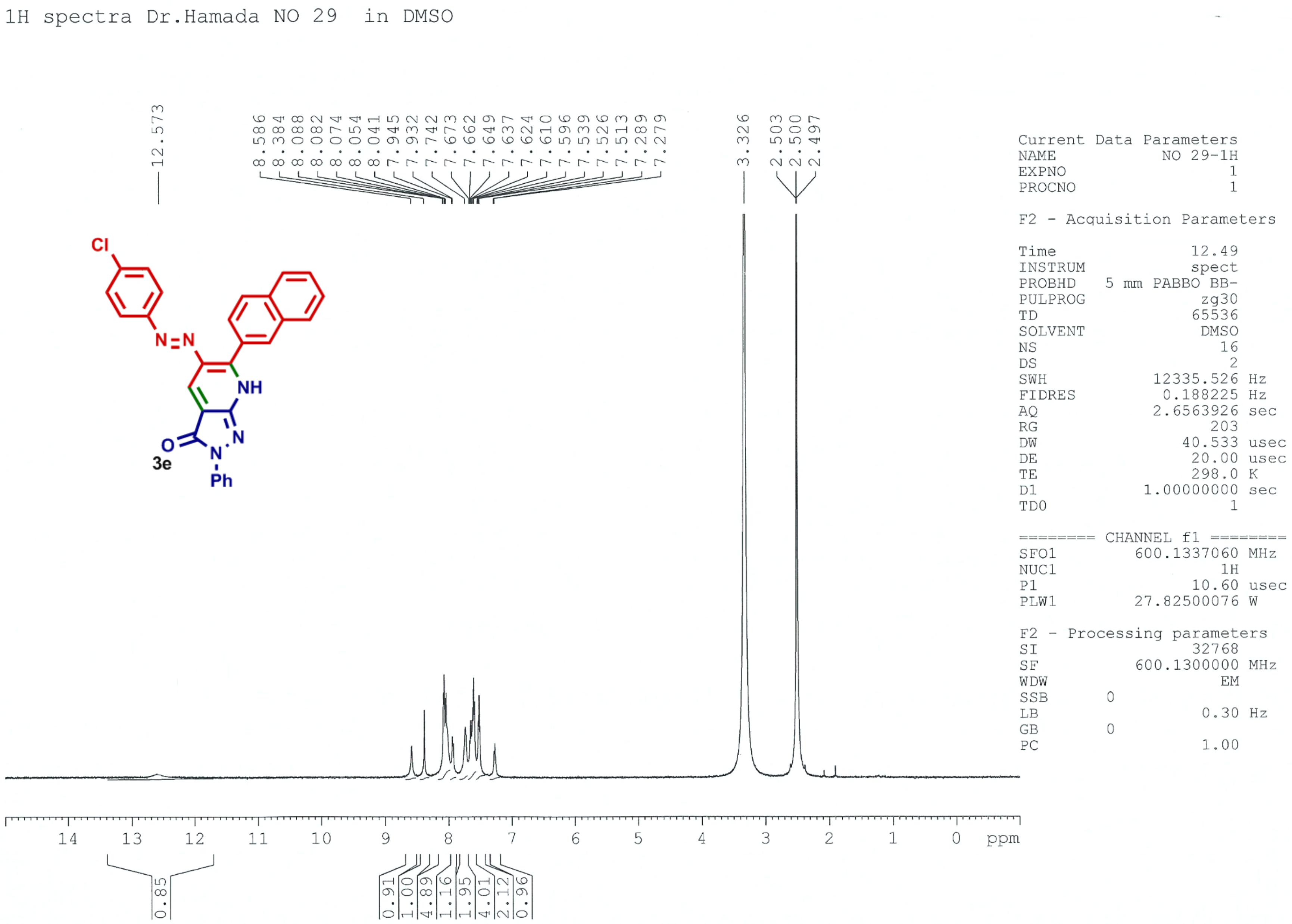

Figure S9. ^{1}H NMR Spectrum (DMSO-d_6, 600 MHz) for compound **3e**.

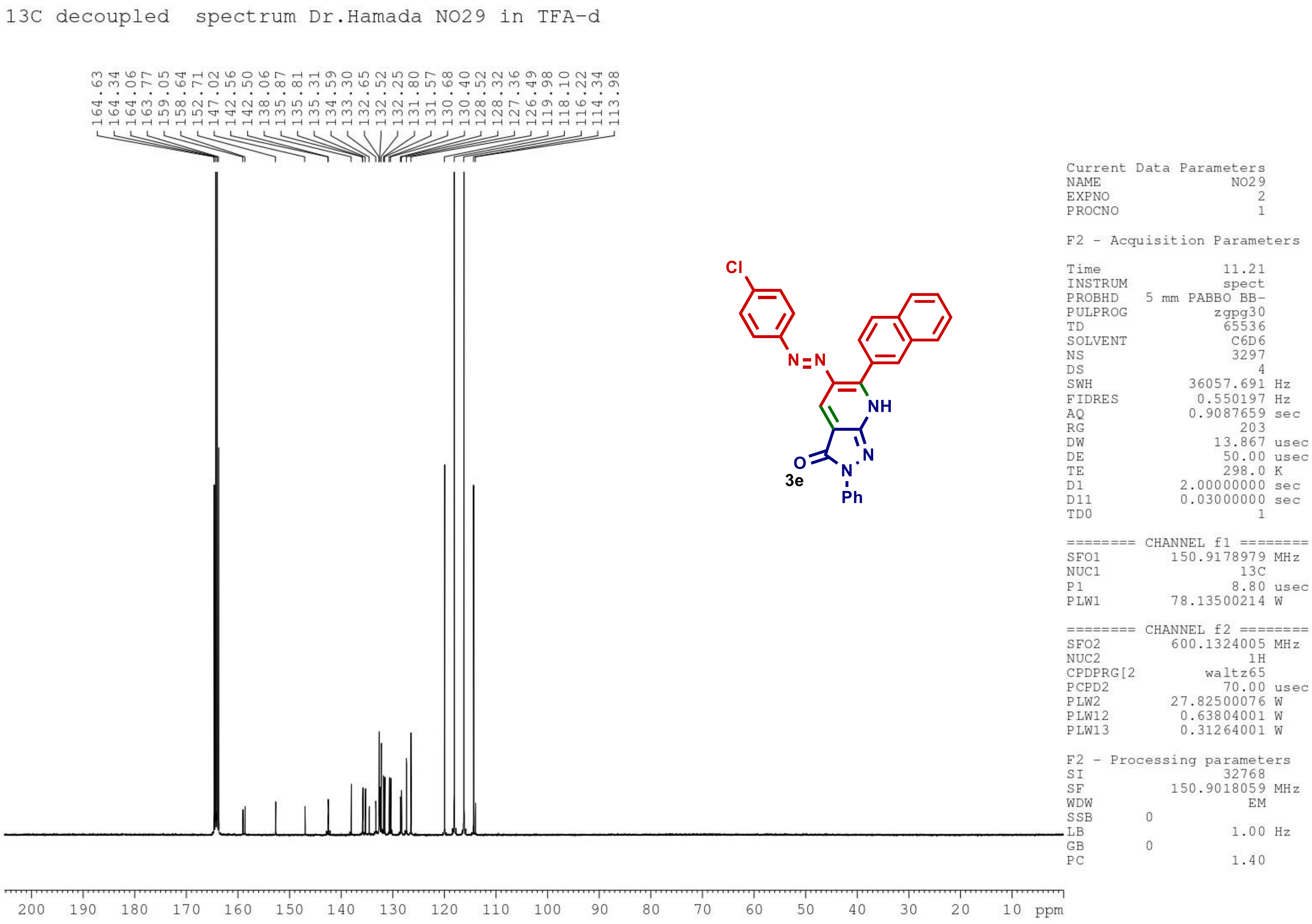

Figure S10. ^{13}C NMR Spectrum (TFA-*d*, 150 MHz) for compound **3e**.

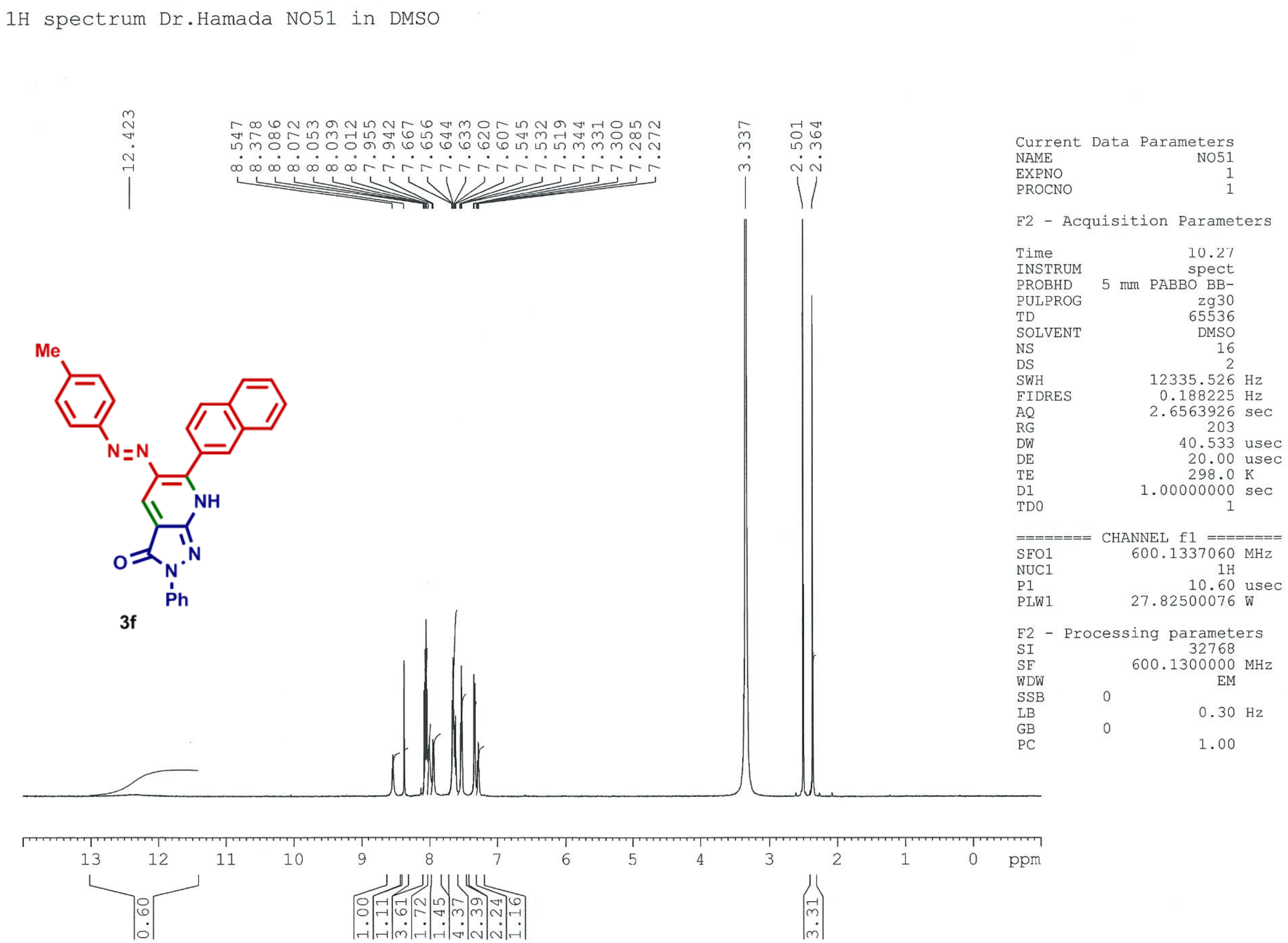

Figure S11. ^{1}H NMR Spectrum (DMSO-d_6, 600 MHz) for compound **3f**.

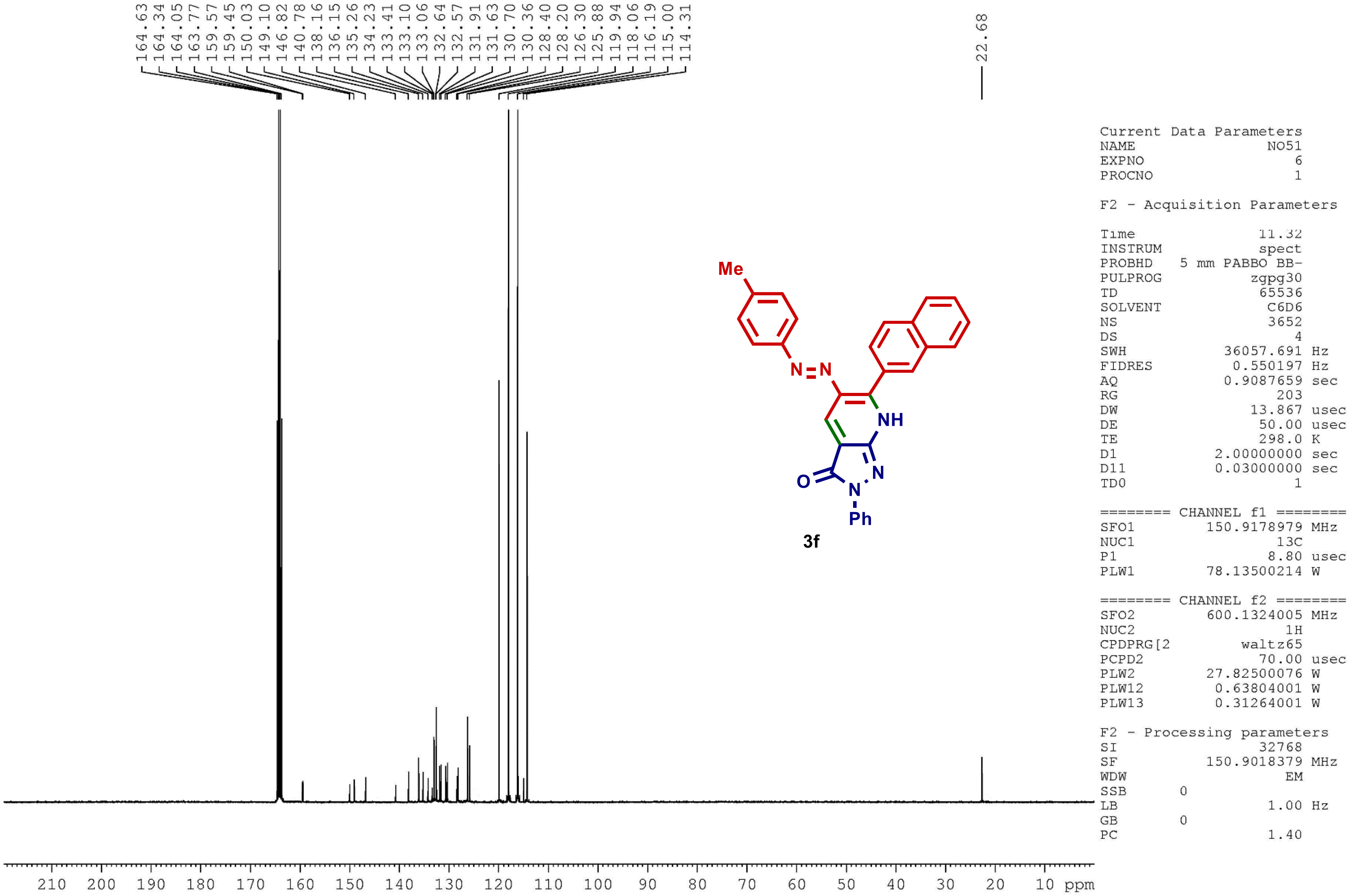

Figure S12. ^{13}C NMR Spectrum (TFA-*d*, 150 MHz) for compound **3f**.

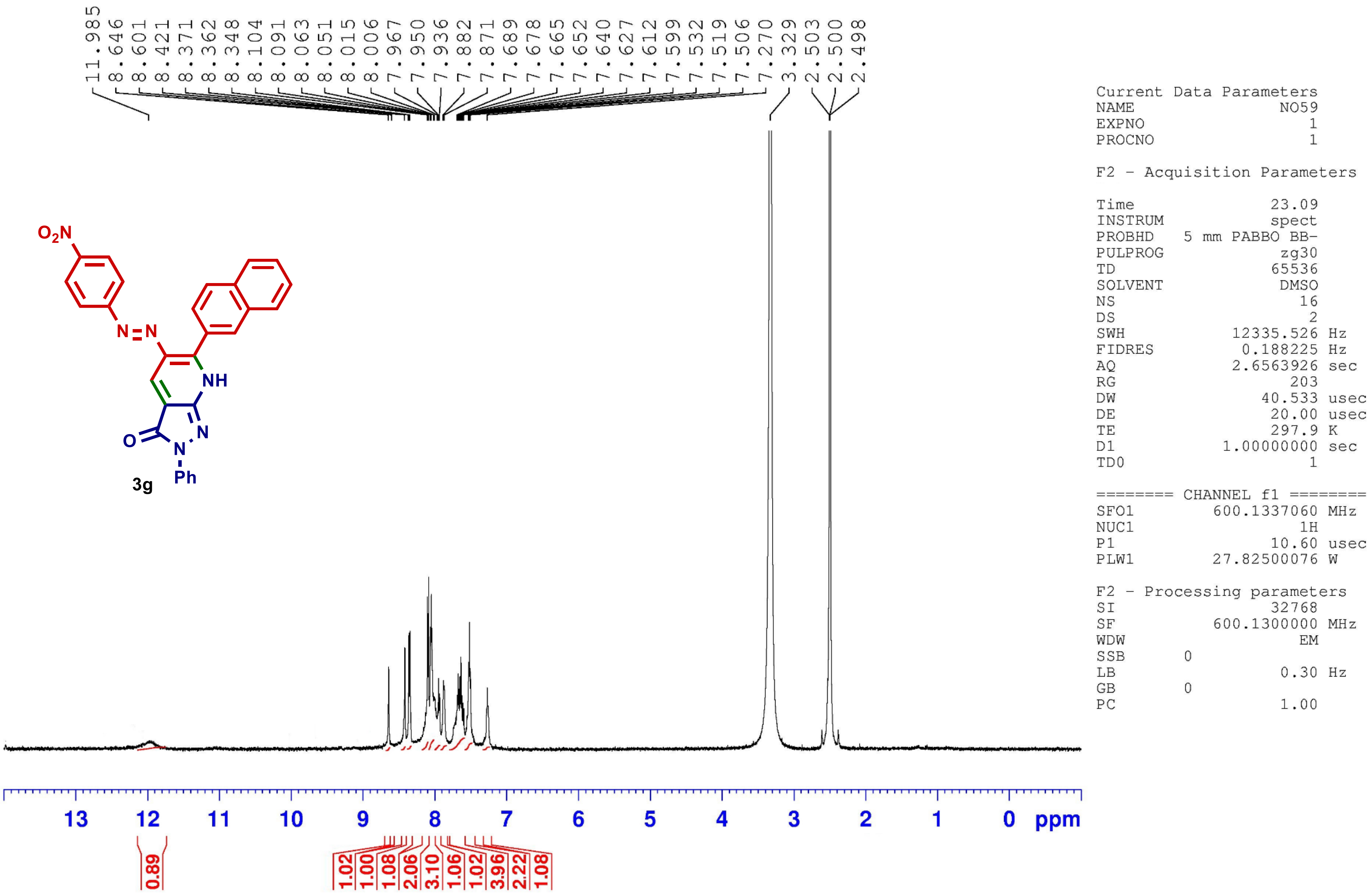

Figure S13. ^{1}H NMR Spectrum (DMSO-d_6, 600 MHz) for compound **3g**.

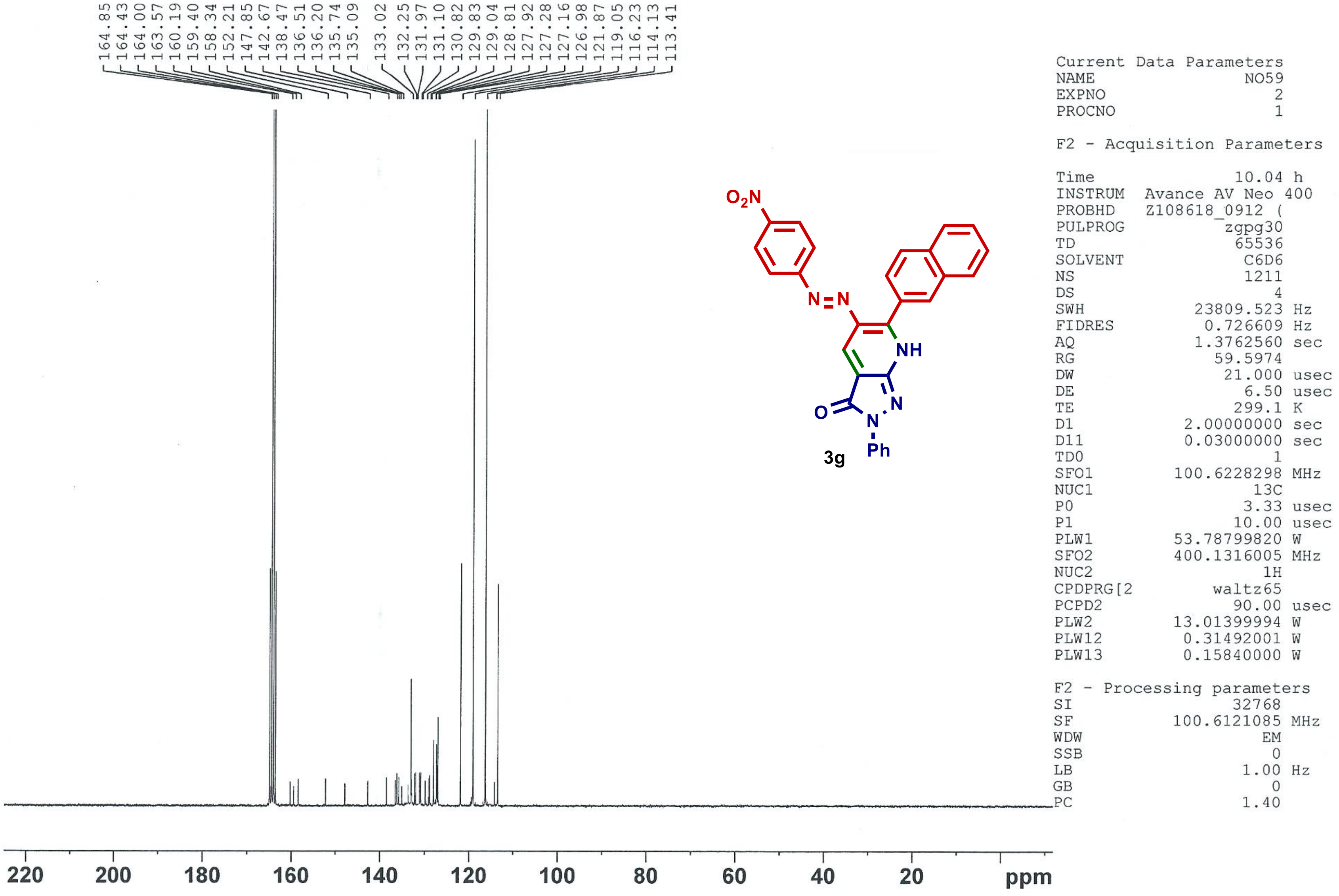

Figure S14. ^{13}C NMR Spectrum (TFA-*d*, 150 MHz) for compound **4g**.

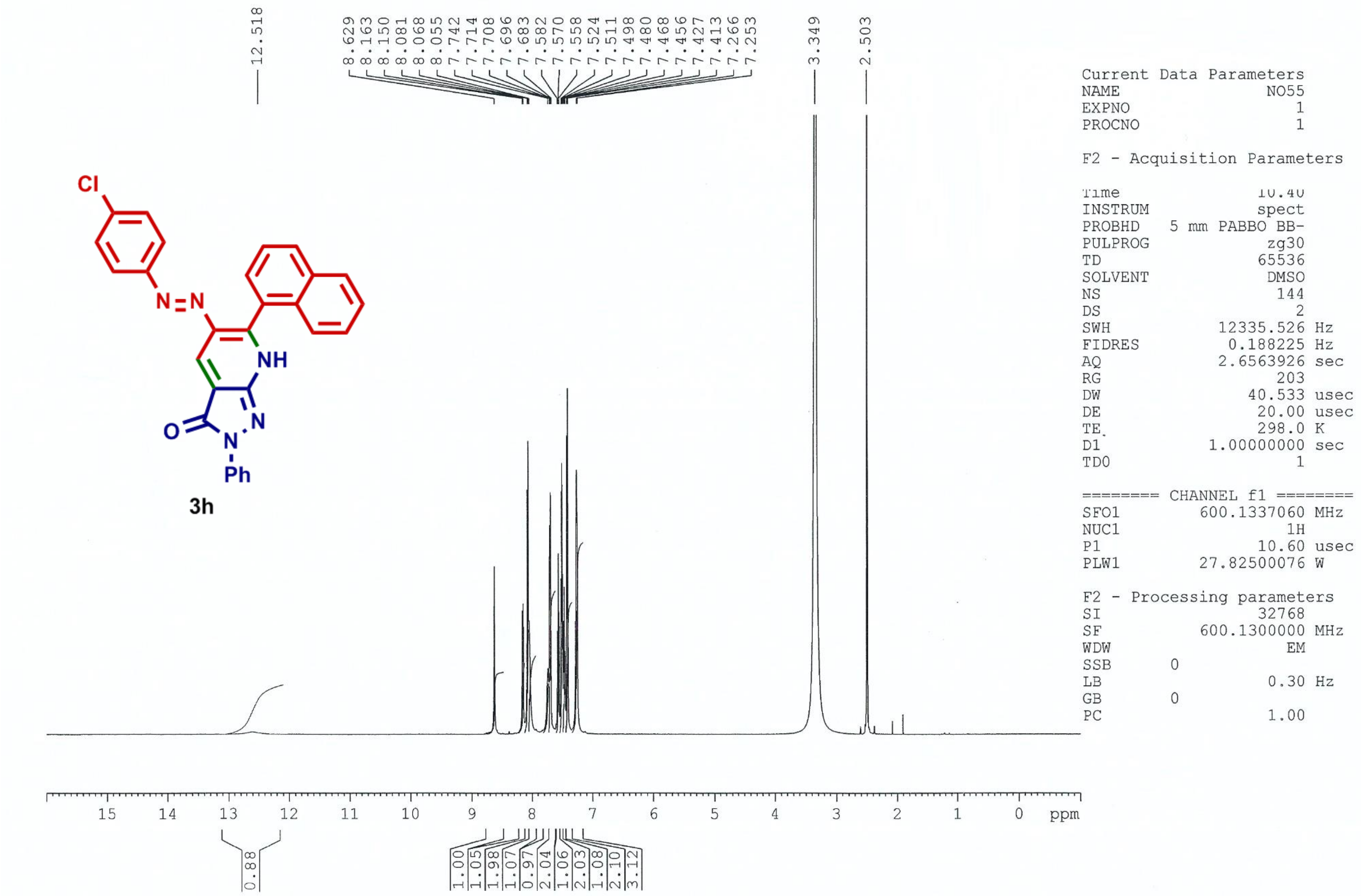

Figure S15. ^{1}H NMR Spectrum (DMSO-d_6, 600 MHz) for compound **3h**.

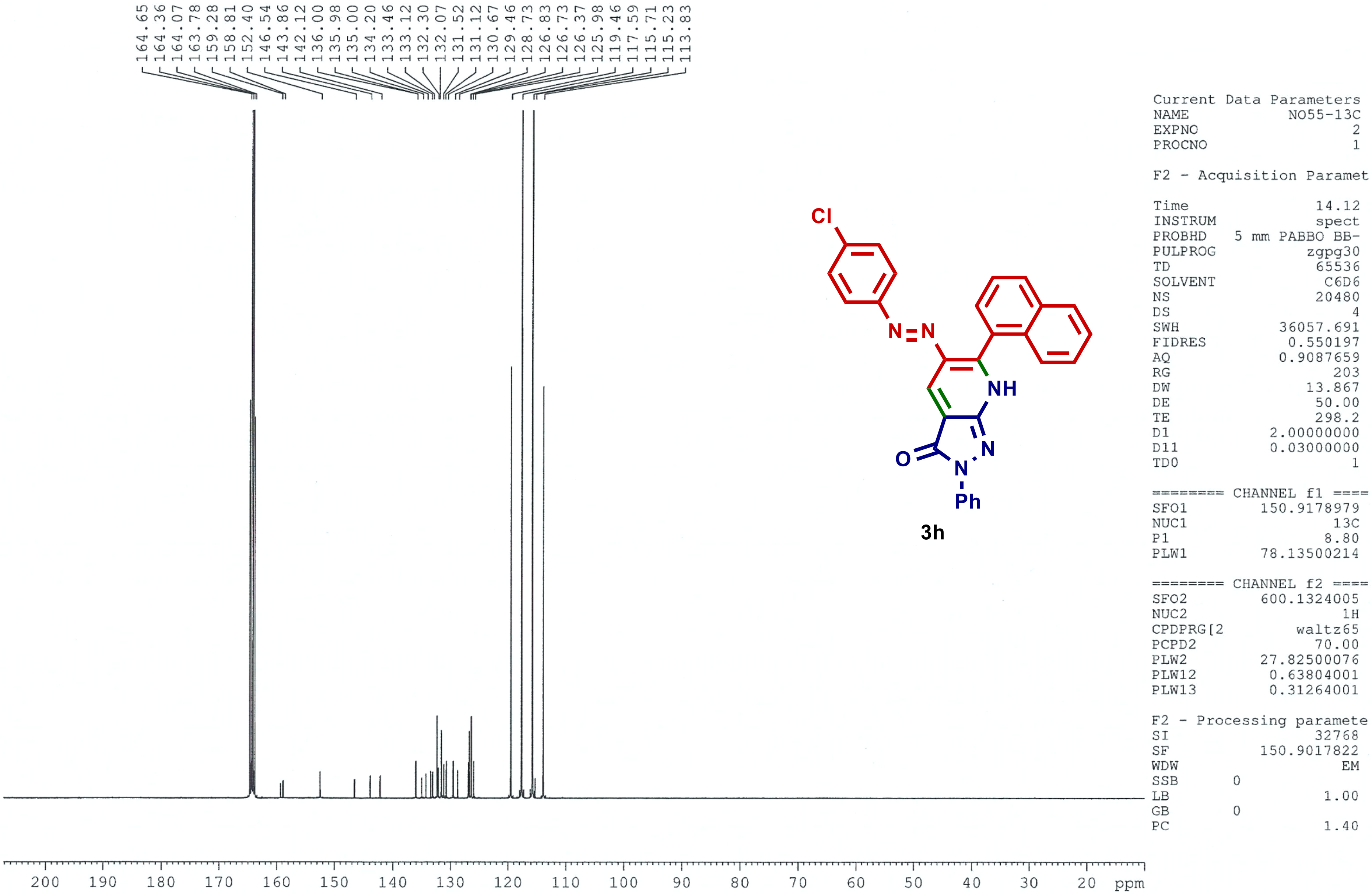

Figure S16. ^{13}C NMR Spectrum (TFA-*d*, 150 MHz) for compound **3h**.

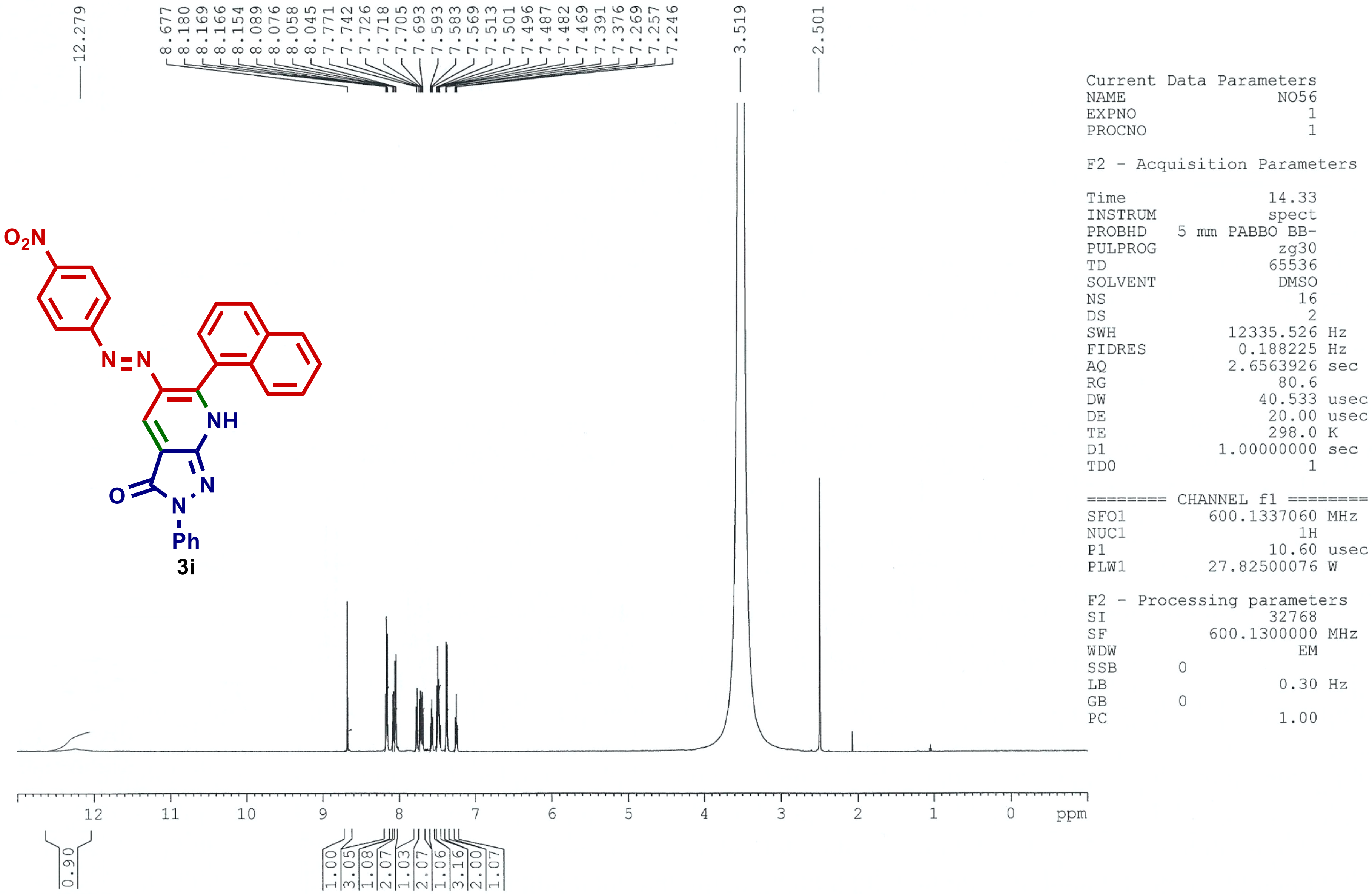

Figure S17. ^{1}H NMR Spectrum (DMSO-d_6, 600 MHz) for compound **3i**.

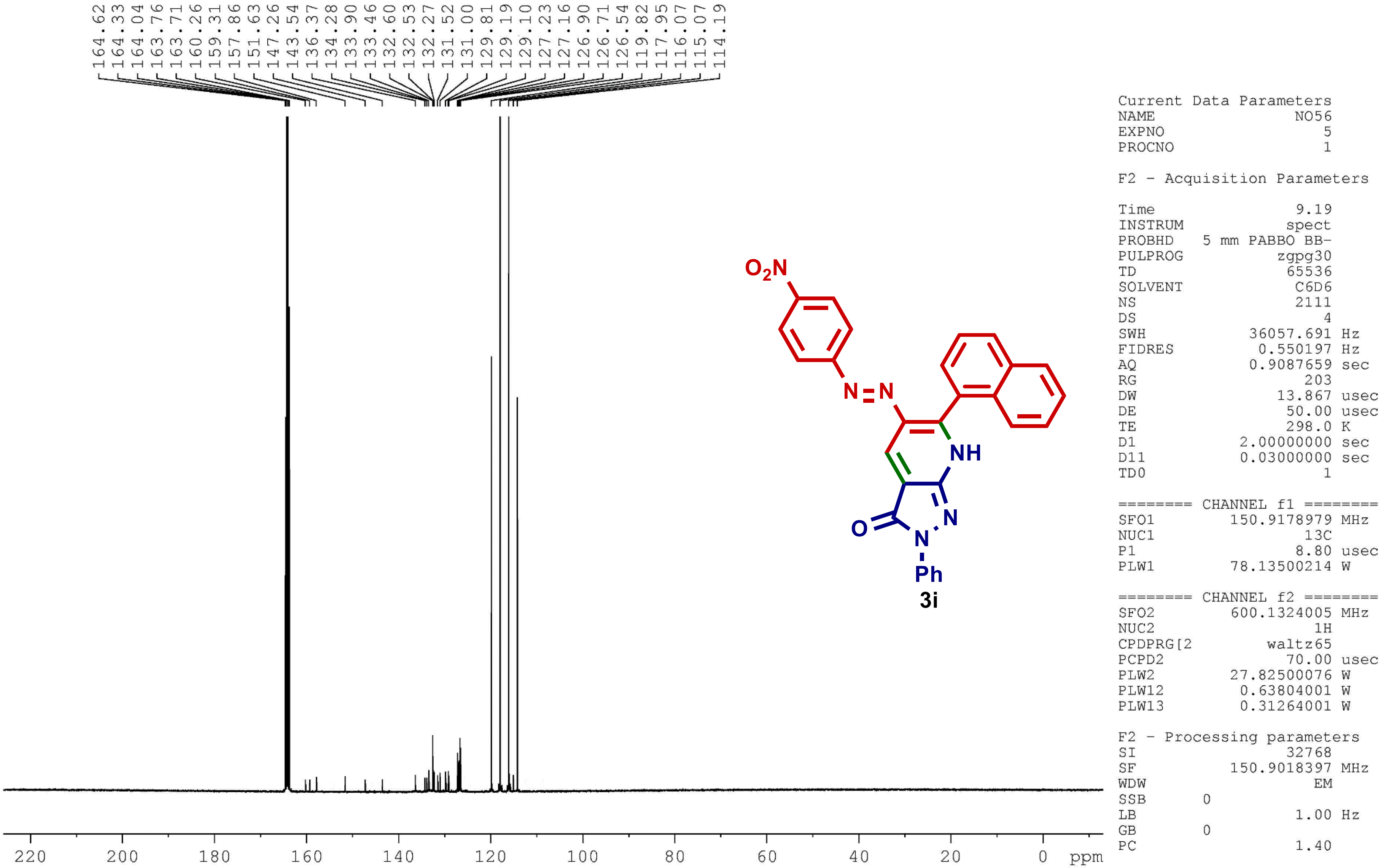

Figure S18. ^{13}C NMR Spectrum (TFA-*d*, 150 MHz) for compound **3i**.

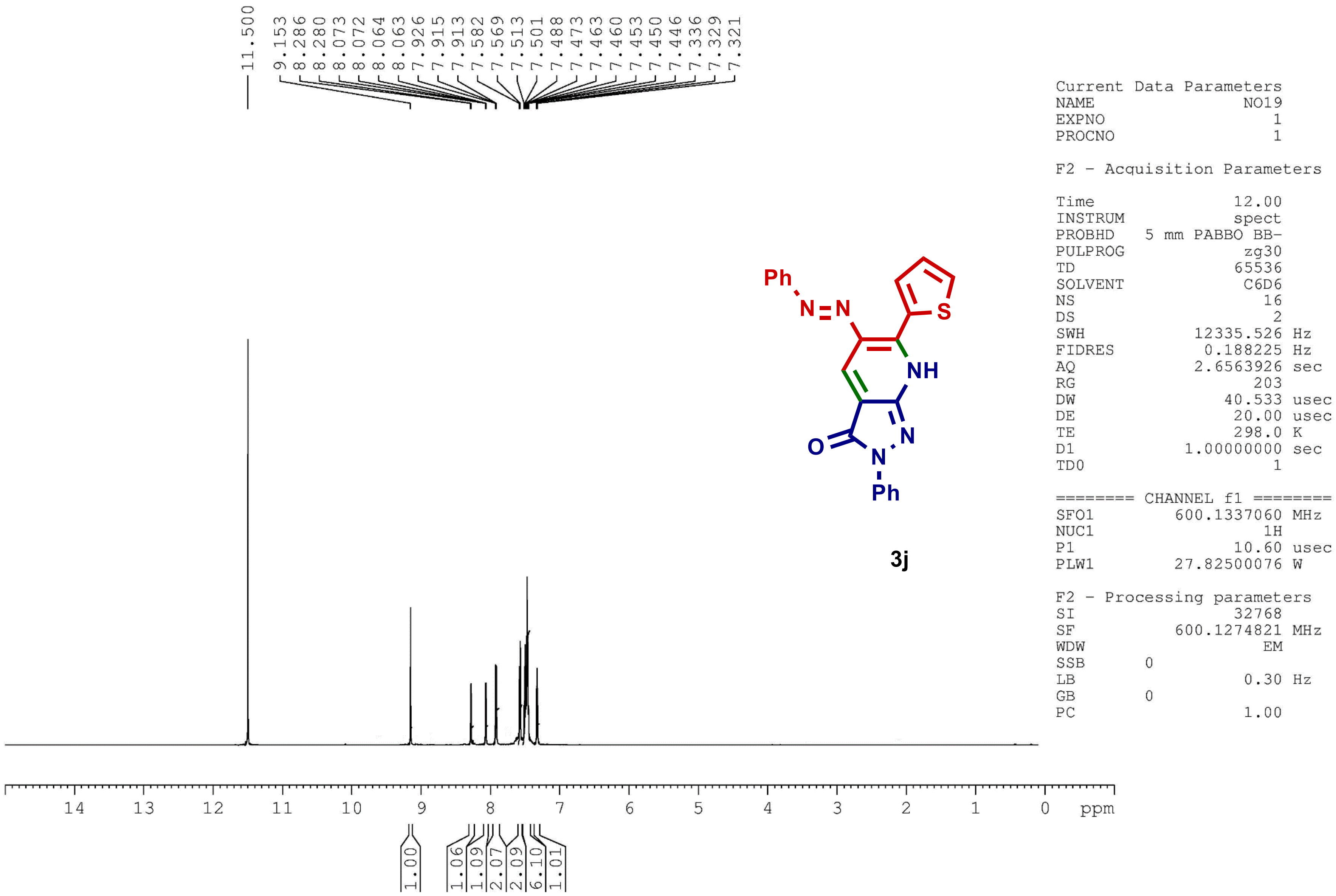

Figure S19. ^{1}H NMR Spectrum (TFA-*d*, 600 MHz) for compound **3j**.

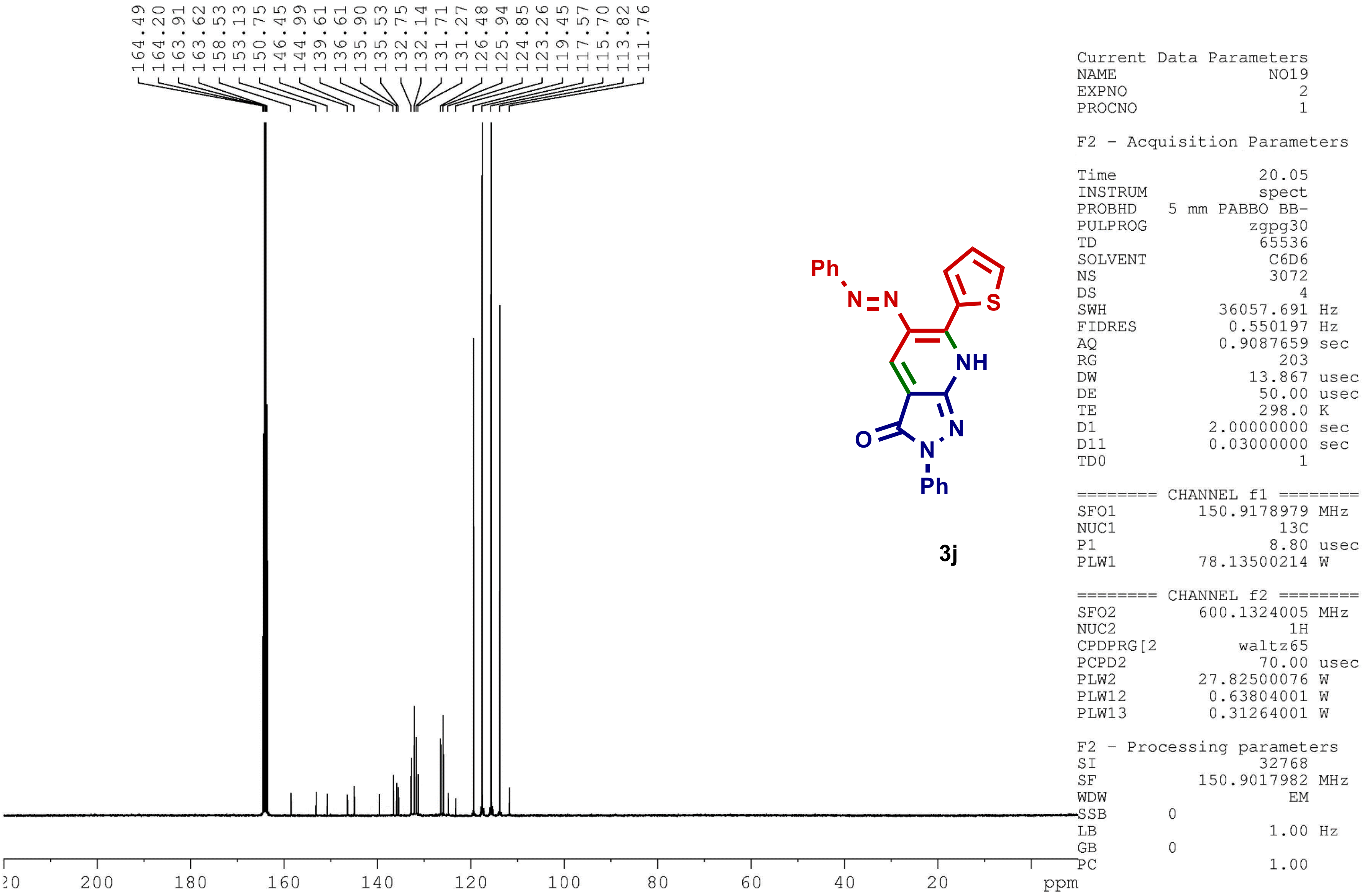

3j

```
Current Data Parameters
NAME                NO19
EXPNO                  2
PROCNO                 1

F2 - Acquisition Parameters
Time               20.05
INSTRUM            spect
PROBHD   5 mm PABBO BB-
PULPROG          zgpg30
TD                 65536
SOLVENT             C6D6
NS                  3072
DS                     4
SWH           36057.691 Hz
FIDRES         0.550197 Hz
AQ            0.9087659 sec
RG                   203
DW                13.867 usec
DE                 50.00 usec
TE                 298.0 K
D1            2.00000000 sec
D11           0.03000000 sec
TD0                    1

======== CHANNEL f1 ========
SFO1         150.9178979 MHz
NUC1                 13C
P1                  8.80 usec
PLW1         78.13500214 W

======== CHANNEL f2 ========
SFO2         600.1324005 MHz
NUC2                  1H
CPDPRG[2         waltz65
PCPD2              70.00 usec
PLW2         27.82500076 W
PLW12         0.63804001 W
PLW13         0.31264001 W

F2 - Processing parameters
SI                 32768
SF           150.9017982 MHz
WDW                   EM
SSB      0
LB                  1.00 Hz
GB       0
PC                  1.00
```

Figure S20. ^{13}C NMR Spectrum (TFA-*d*, 150 MHz) for compound **3j**.

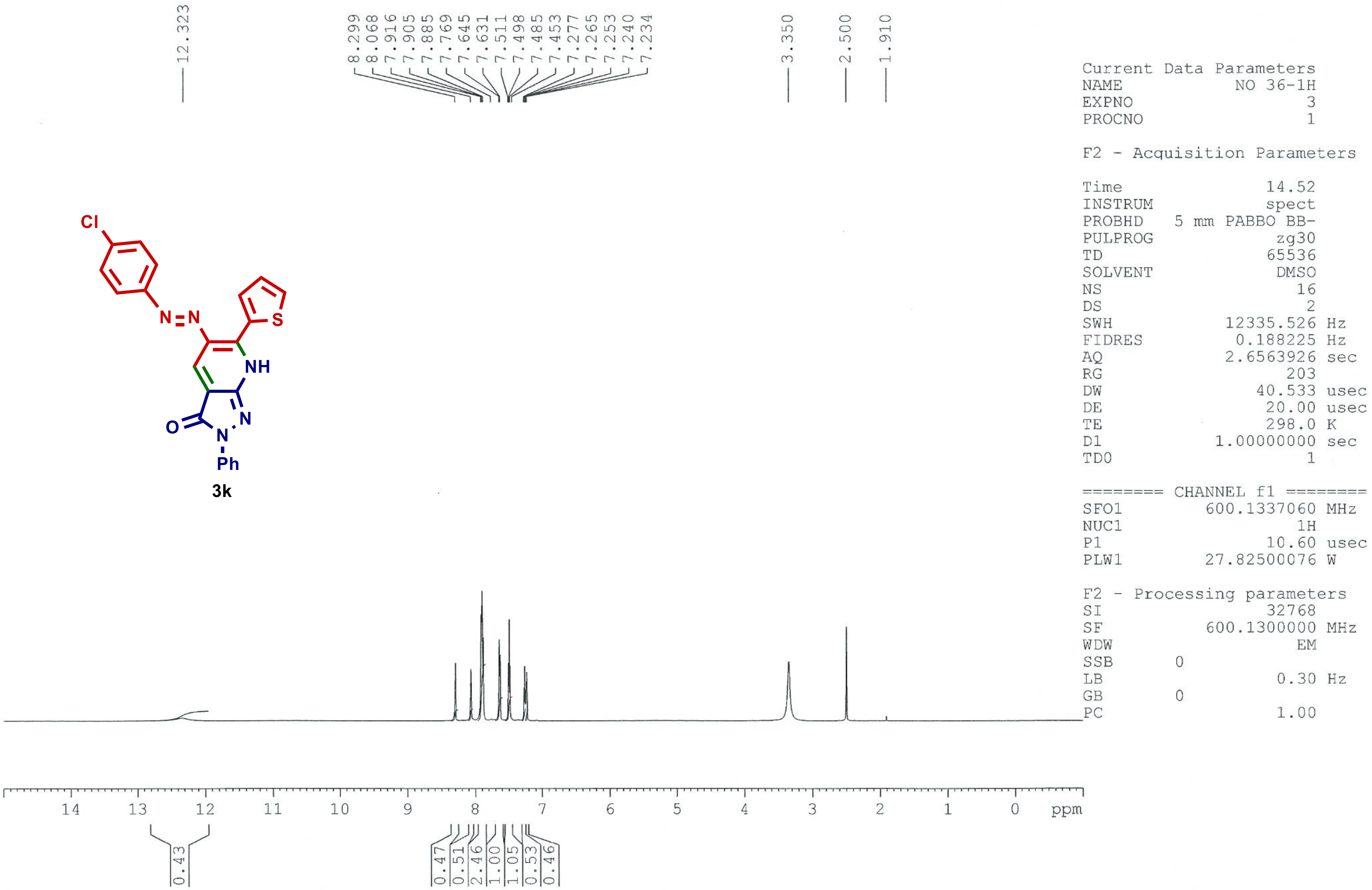

Figure S21. ^{1}H NMR Spectrum ((DMSO-d_6, 600 MHz) for compound **3k**.

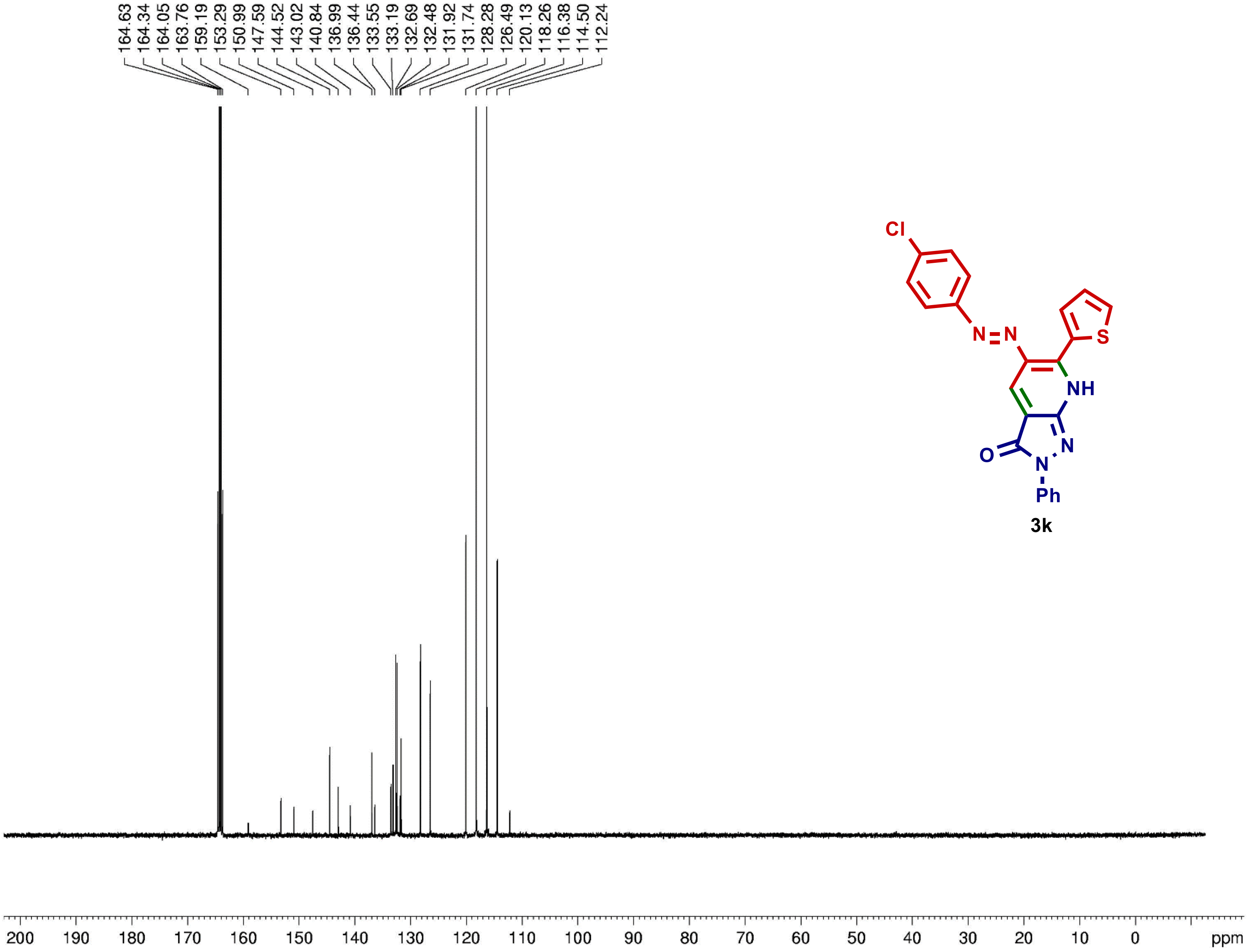

3k

Figure S22. ^{13}C NMR Spectrum (TFA-*d*, 150 MHz) for compound **3k**.

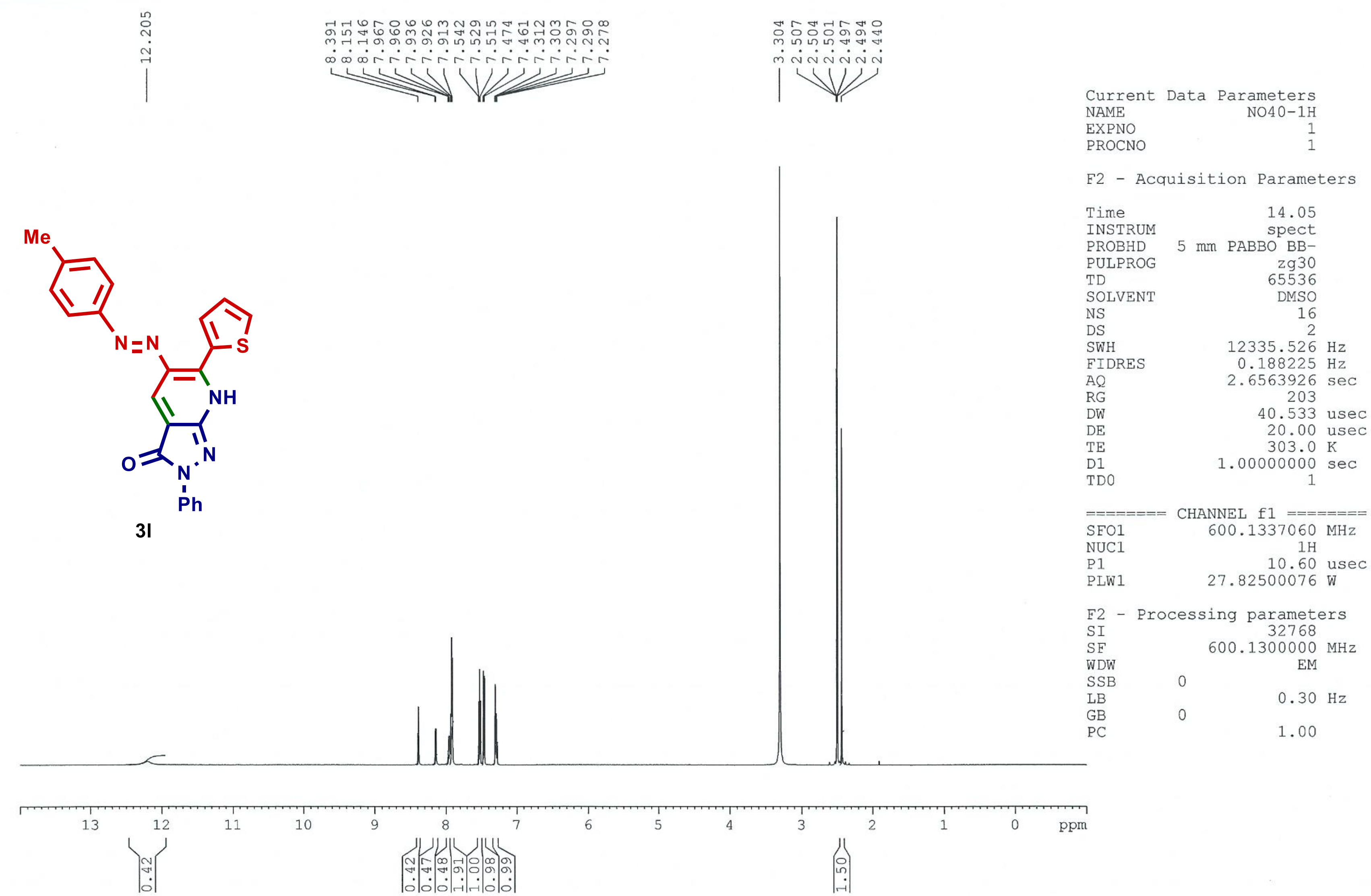

Figure S23. ^{1}H NMR Spectrum (DMSO-d_6, 600 MHz) for compound **3l**.

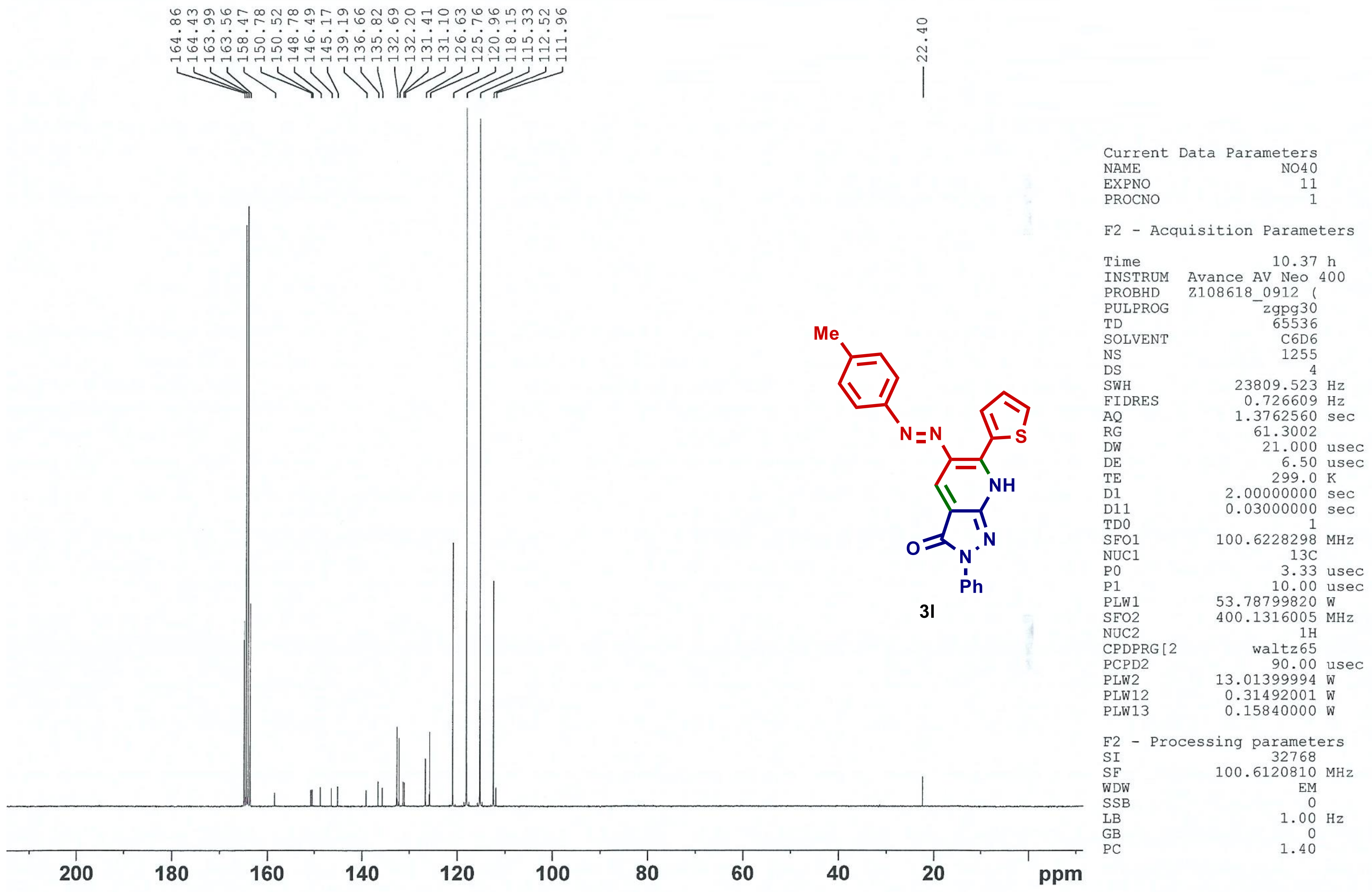

Figure S24. ^{13}C NMR Spectrum (TFA-*d*, 150 MHz) for compound **3l**.

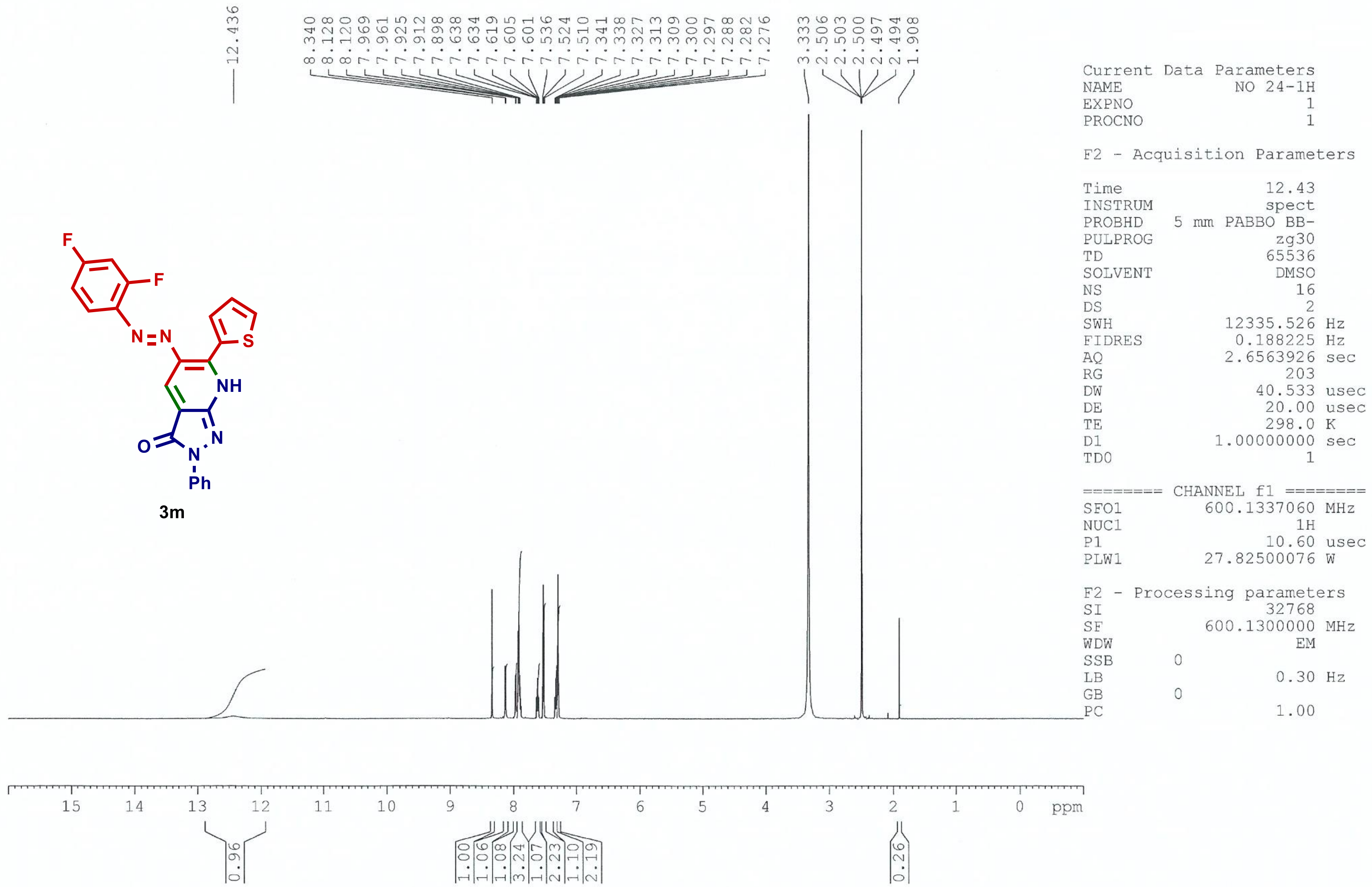

Figure S25. ^{1}H NMR Spectrum (DMSO-d_6, 600 MHz) for compound **3m**.

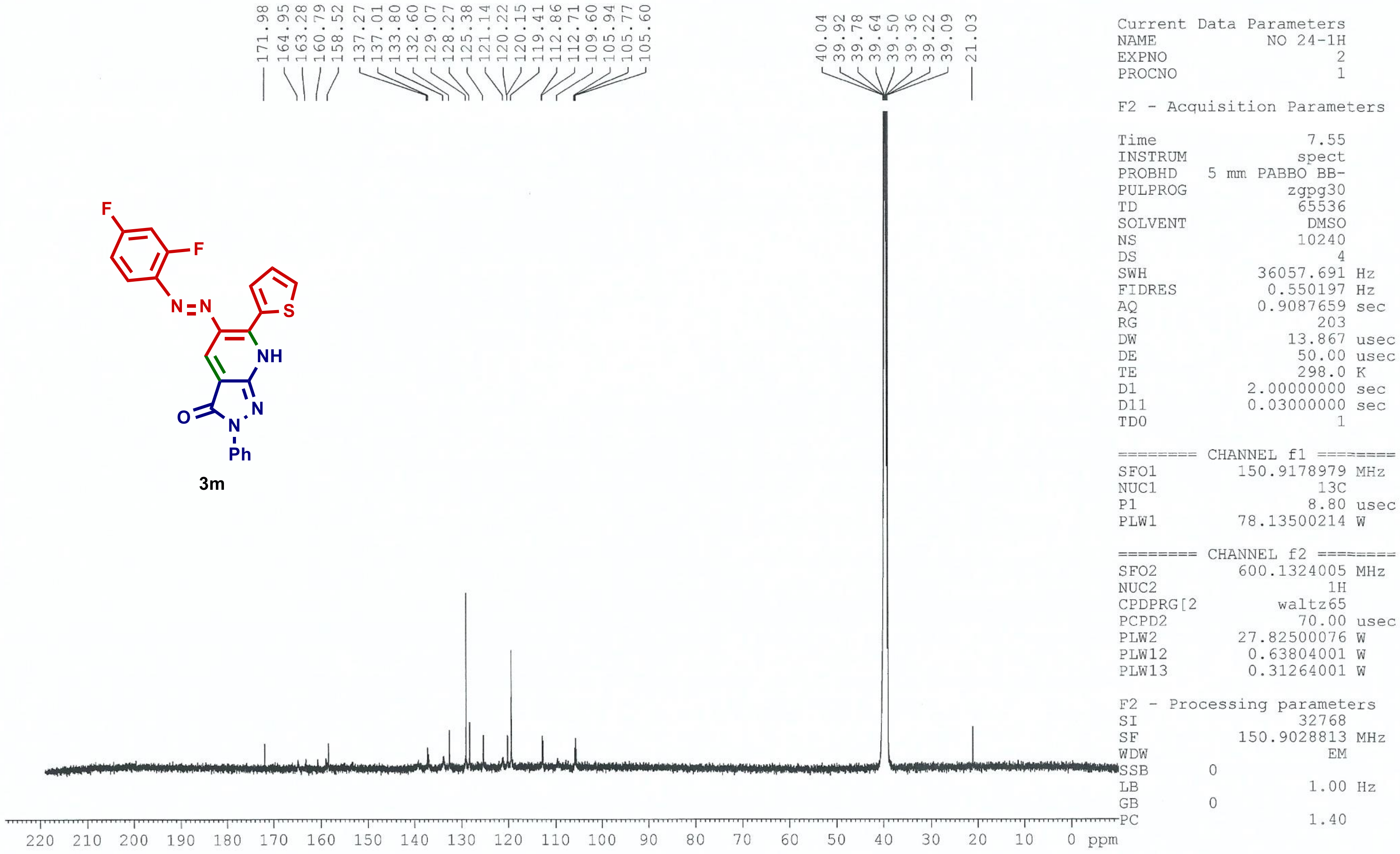

Figure S26. ^{13}C NMR Spectrum (DMSO-*d6*, 150 MHz) for compound **3m**.

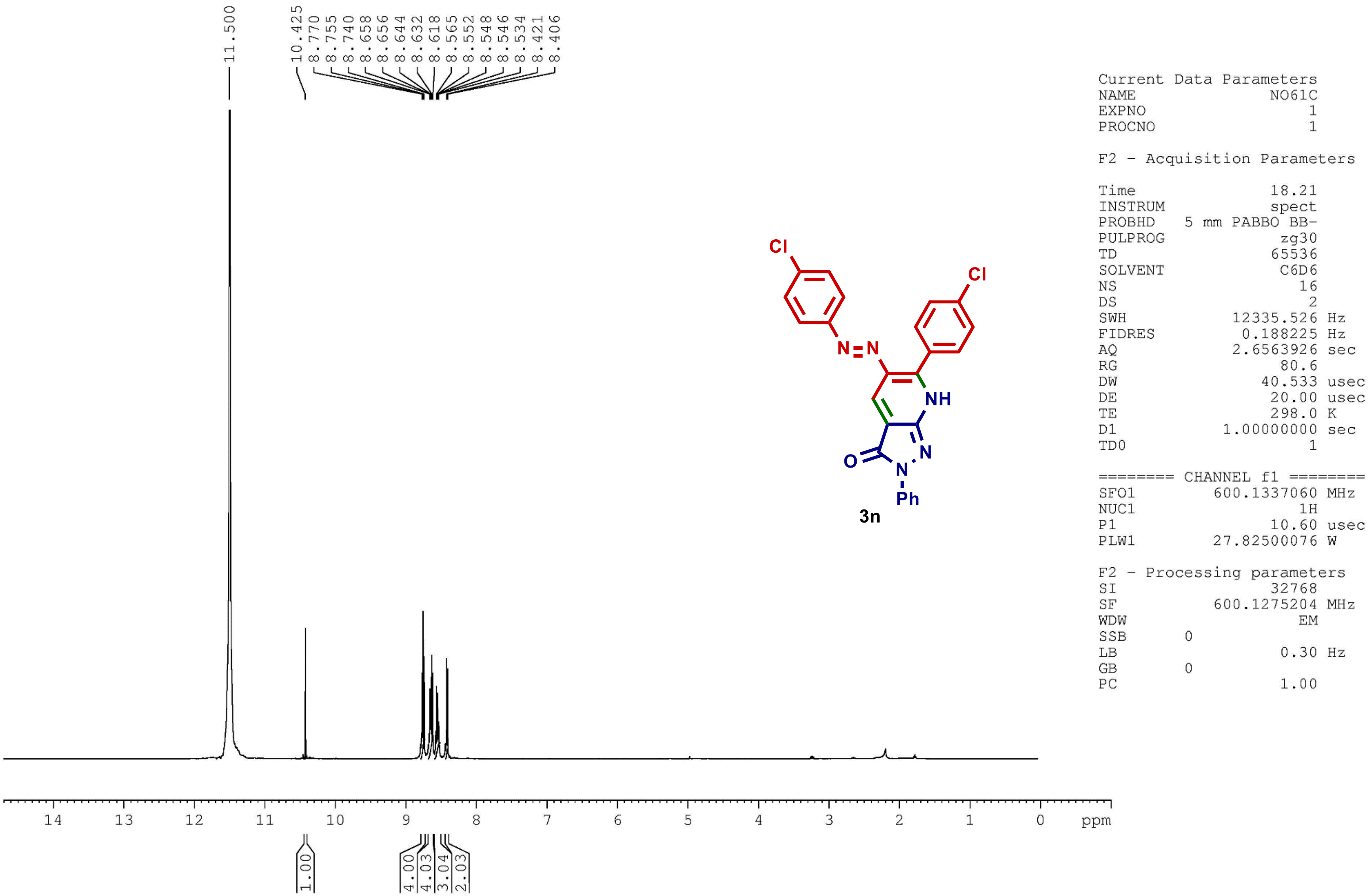

Figure S27. ^{1}H NMR Spectrum (TFA-*d*, 600 MHz) for compound **3n**.

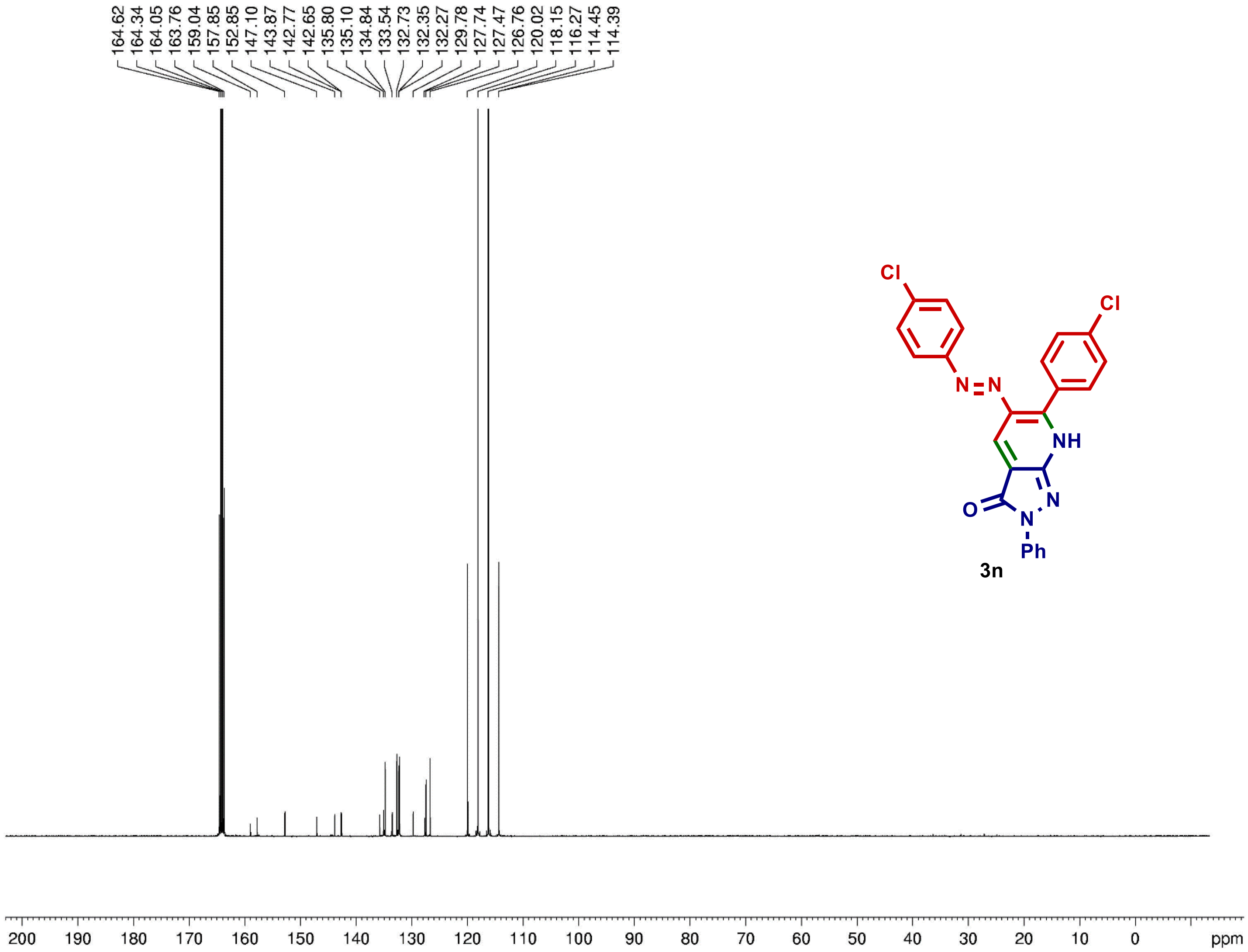

3n

Current Data Parameters
NAME NO61C
EXPNO 2
PROCNO 1

F2 – Acquisition Parameters

Time	22.37
INSTRUM	spect
PROBHD	5 mm PABBO BB–
PULPROG	zgpg30
TD	65536
SOLVENT	C6D6
NS	5120
DS	4
SWH	36057.691 Hz
FIDRES	0.550197 Hz
AQ	0.9087659 sec
RG	203
DW	13.867 usec
DE	50.00 usec
TE	298.0 K
D1	2.00000000 sec
D11	0.03000000 sec
TD0	1

======== CHANNEL f1 ========

SFO1	150.9178979 MHz
NUC1	13C
P1	8.80 usec
PLW1	78.13500214 W

======== CHANNEL f2 ========

SFO2	600.1324005 MHz
NUC2	1H
CPDPRG[2	waltz65
PCPD2	70.00 usec
PLW2	27.82500076 W
PLW12	0.63804001 W
PLW13	0.31264001 W

F2 – Processing parameters

SI	32768
SF	150.9018819 MHz
WDW	EM
SSB	0
LB	1.00 Hz
GB	0
PC	1.40

Figure S28. ^{13}C NMR Spectrum (TFA-*d*, 150 MHz) for compound **3n**.

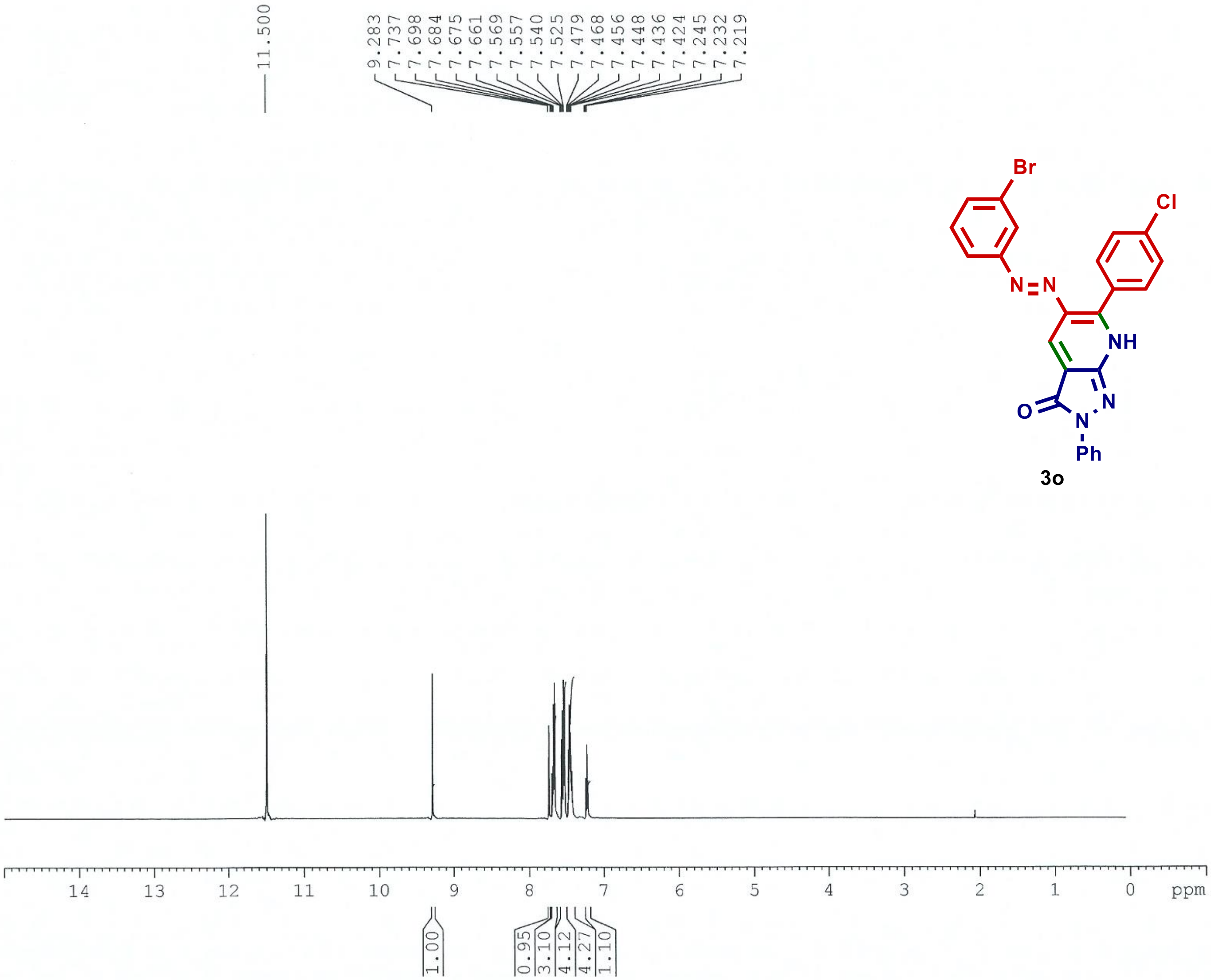

3o

```
Current Data Parameters
NAME              NO44-1H
EXPNO                   1
PROCNO                  1

F2 - Acquisition Parameters
Time                12.08
INSTRUM             spect
PROBHD     5 mm PABBO BB-
PULPROG              zg30
TD                  65536
SOLVENT              C6D6
NS                      8
DS                      2
SWH           12335.526 Hz
FIDRES         0.188225 Hz
AQ            2.6563926 sec
RG                    161
DW               40.533 usec
DE                20.00 usec
TE                298.0 K
D1           1.00000000 sec
TD0                     1

======== CHANNEL f1 ========
SFO1        600.1337060 MHz
NUC1                   1H
P1                  10.60 usec
PLW1          27.82500076 W

F2 - Processing parameters
SI                  32768
SF          600.1274997 MHz
WDW                    EM
SSB         0
LB                   0.30 Hz
GB          0
PC                   1.00
```

Figure S29. ^{1}H NMR Spectrum (TFA-*d*, 600 MHz) for compound **3o**.

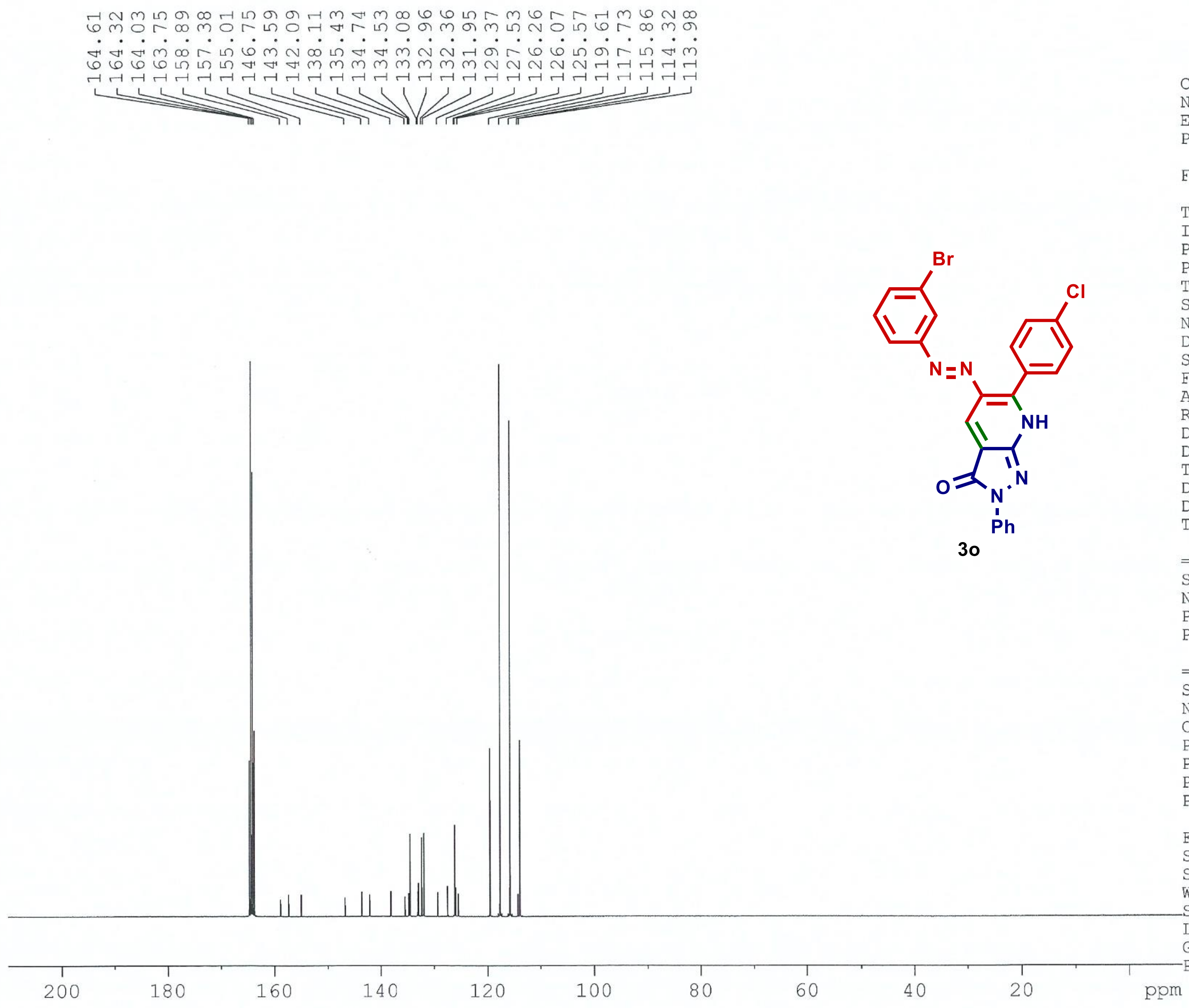

3o

Figure S30. ^{13}C NMR Spectrum (TFA-*d*, 150 MHz) for compound **3o**.

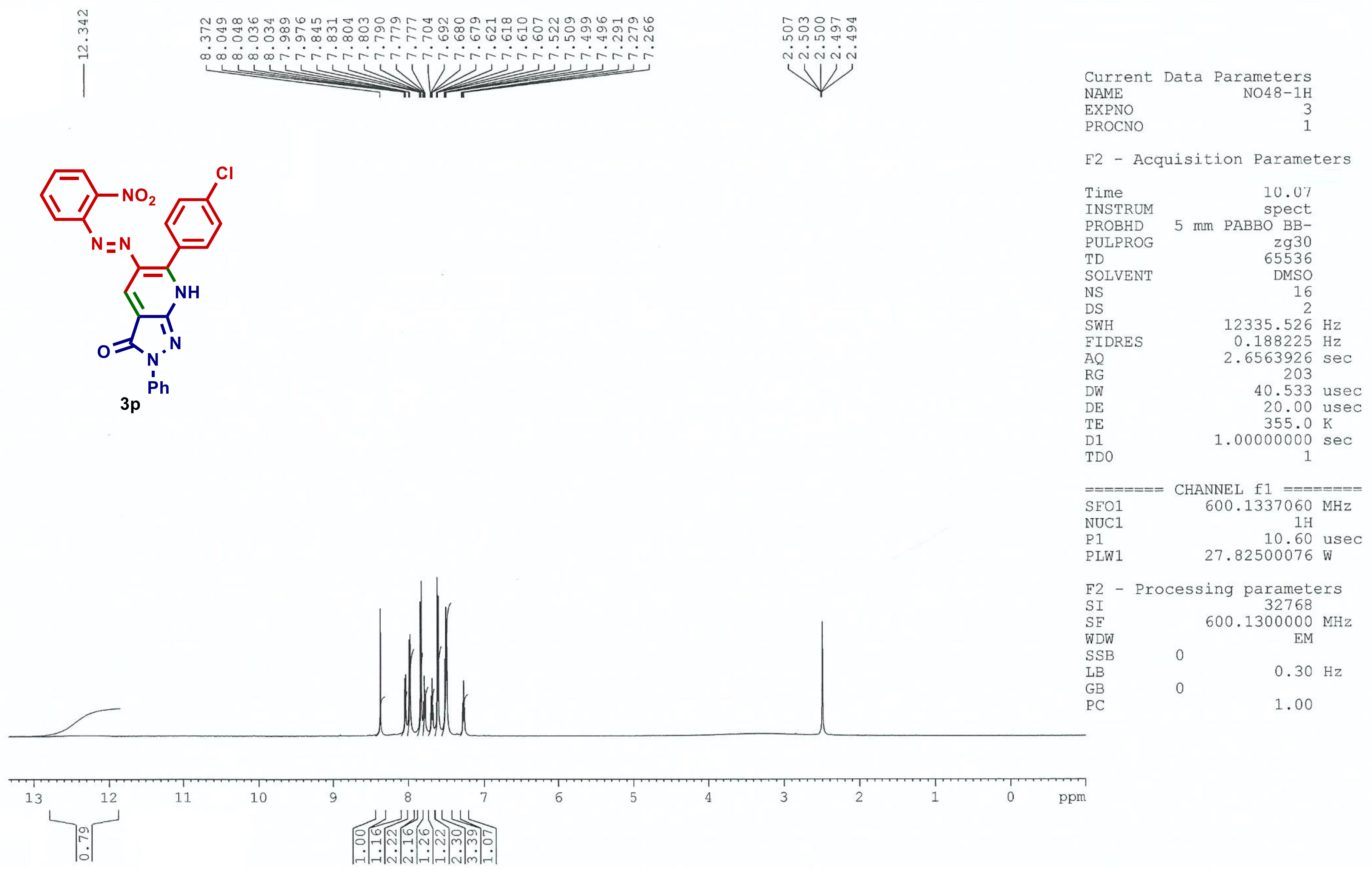

Figure S31. ^{1}H NMR Spectrum (DMSO-*d6*, 600 MHz) for compound **3p**.

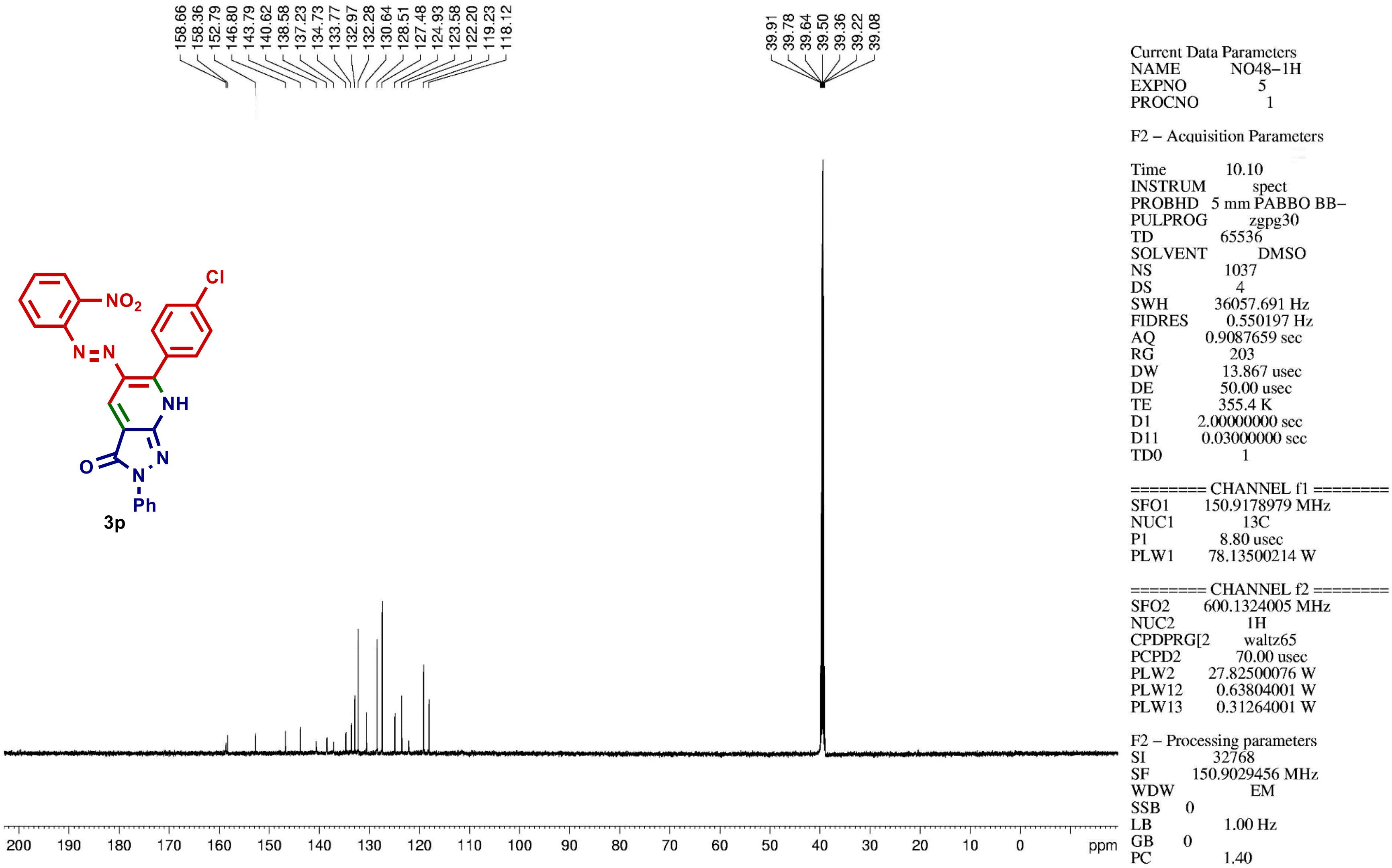

Figure S32. ^{13}C NMR Spectrum (DMSO-*d6*, 150 MHz) for compound **3p**.

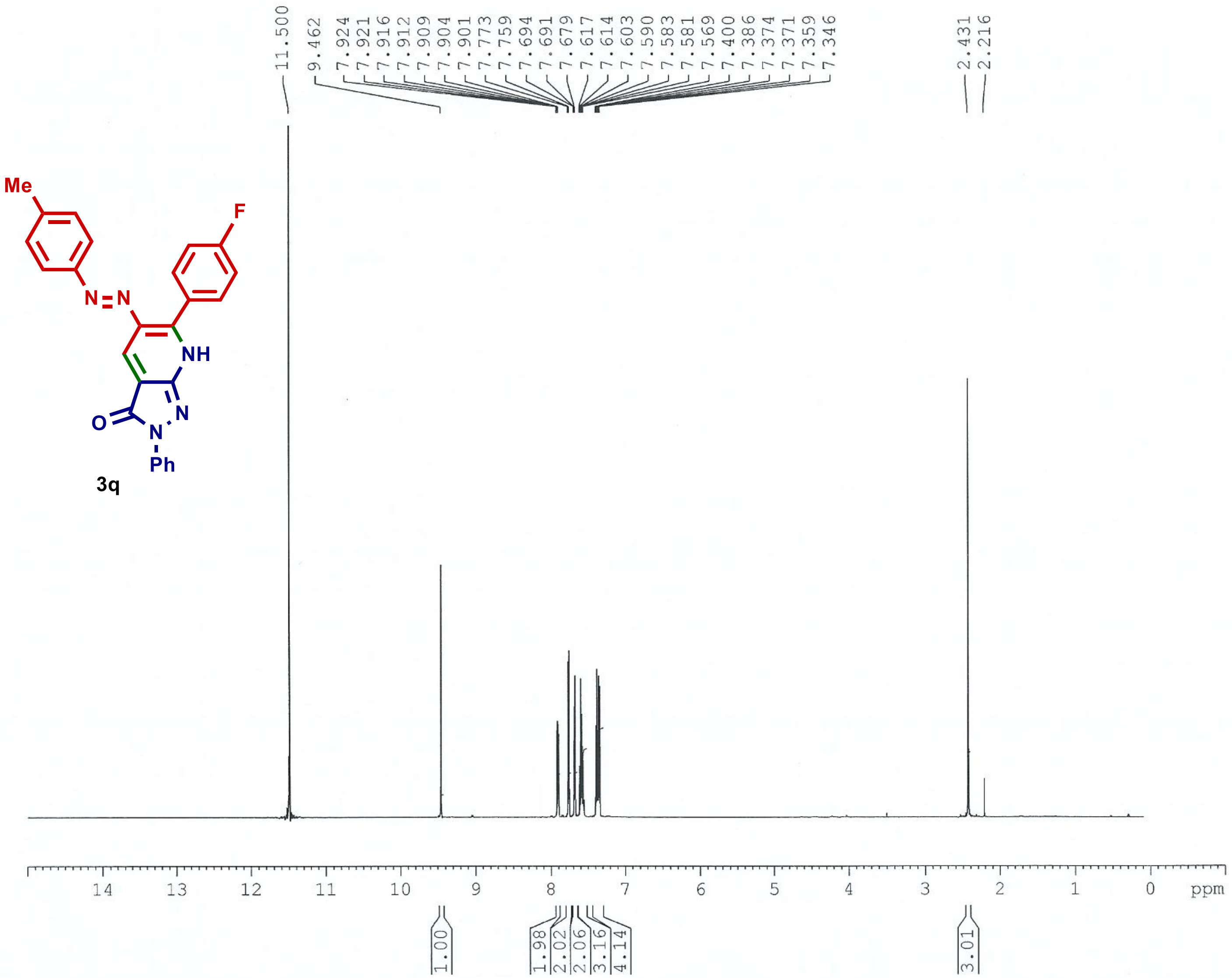


```
Current Data Parameters
NAME                NO14-1H
EXPNO                     1
PROCNO                    1

F2 - Acquisition Parameters
Time                  12.14
INSTRUM               spect
PROBHD    5 mm PABBO BB-
PULPROG                zg30
TD                    65536
SOLVENT                C6D6
NS                        8
DS                        2
SWH            12335.526 Hz
FIDRES          0.188225 Hz
AQ             2.6563926 sec
RG                      203
DW                40.533 usec
DE                 20.00 usec
TE                 298.0 K
D1            1.00000000 sec
TD0                       1

======== CHANNEL f1 ========
SFO1         600.1337060 MHz
NUC1                     1H
P1                 10.60 usec
PLW1         27.82500076 W

F2 - Processing parameters
SI                    32768
SF           600.1274823 MHz
WDW                      EM
SSB        0
LB                  0.30 Hz
GB         0
PC                     1.00
```

Figure S33. ^{1}H NMR Spectrum (TFA-*d*, 600 MHz) for compound **3q**.

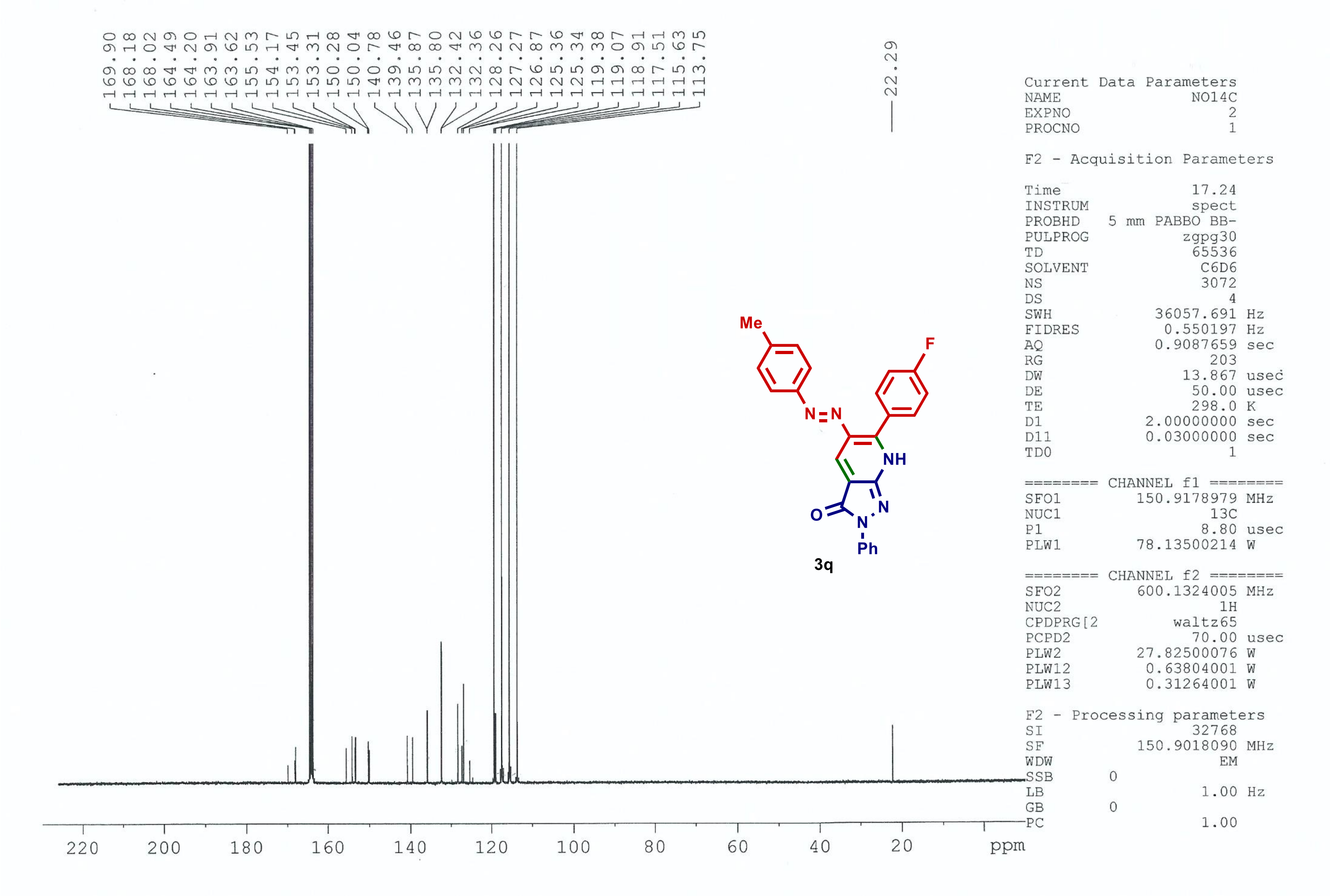

Figure S34. ^{13}C NMR Spectrum (TFA-*d*, 150 MHz) for compound **3q**.

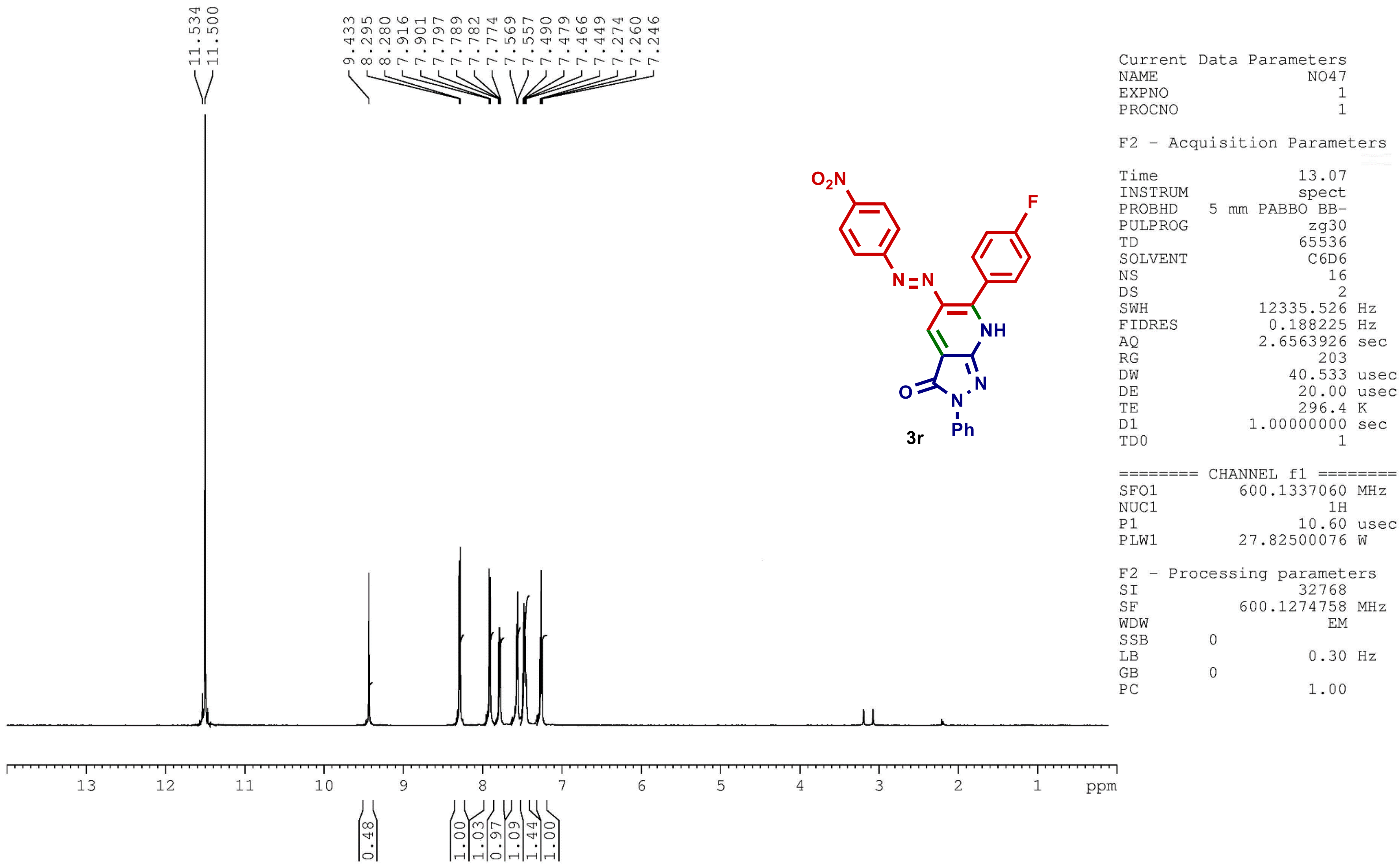

3r

Current Data Parameters
NAME NO47
EXPNO 1
PROCNO 1

F2 - Acquisition Parameters

Time 13.07
INSTRUM spect
PROBHD 5 mm PABBO BB-
PULPROG zg30
TD 65536
SOLVENT C6D6
NS 16
DS 2
SWH 12335.526 Hz
FIDRES 0.188225 Hz
AQ 2.6563926 sec
RG 203
DW 40.533 usec
DE 20.00 usec
TE 296.4 K
D1 1.00000000 sec
TD0 1

======== CHANNEL f1 ========
SFO1 600.1337060 MHz
NUC1 1H
P1 10.60 usec
PLW1 27.82500076 W

F2 - Processing parameters
SI 32768
SF 600.1274758 MHz
WDW EM
SSB 0
LB 0.30 Hz
GB 0
PC 1.00

Figure S35. ^{1}H NMR Spectrum (TFA-*d*, 600 MHz) for compound **4r**.

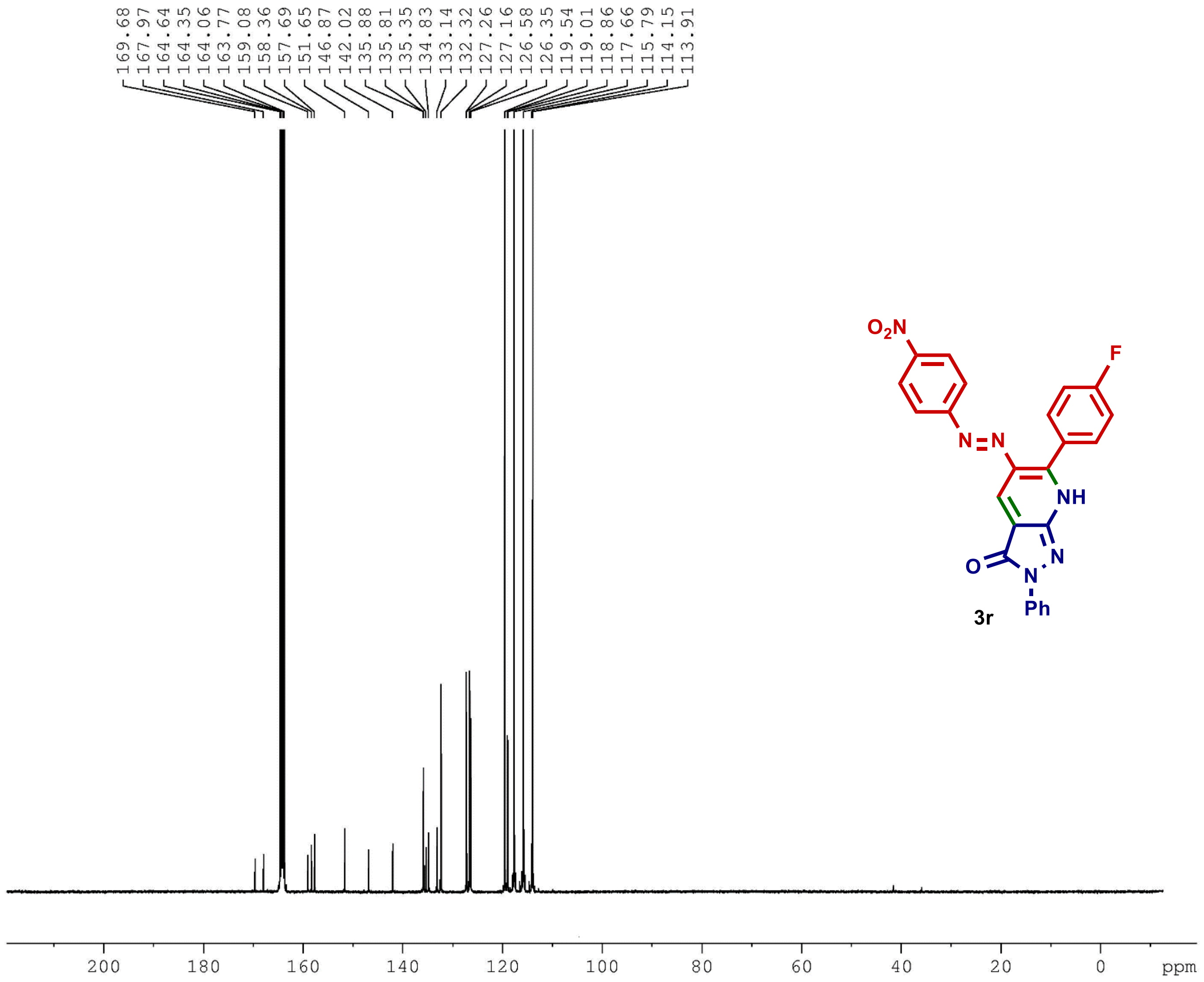

Current Data Parameters
NAME NO47
EXPNO 2
PROCNO 1

F2 - Acquisition Parameters

Time 21.37
INSTRUM spect
PROBHD 5 mm PABBO BB-
PULPROG zgpg30
TD 65536
SOLVENT C6D6
NS 10240
DS 4
SWH 36057.691 Hz
FIDRES 0.550197 Hz
AQ 0.9087659 sec
RG 203
DW 13.867 usec
DE 50.00 usec
TE 299.1 K
D1 2.00000000 sec
D11 0.03000000 sec
TD0 1

======== CHANNEL f1 ========
SFO1 150.9178979 MHz
NUC1 13C
P1 8.80 usec
PLW1 78.13500214 W

======== CHANNEL f2 ========
SFO2 600.1324005 MHz
NUC2 1H
CPDPRG[2 waltz65
PCPD2 70.00 usec
PLW2 27.82500076 W
PLW12 0.63804001 W
PLW13 0.31264001 W

F2 - Processing parameters
SI 32768
SF 150.9017695 MHz
WDW EM
SSB 0
LB 1.00 Hz
GB 0
PC 1.40

Figure S36. ^{13}C NMR Spectrum (TFA-*d*, 150 MHz) for compound **4r**.

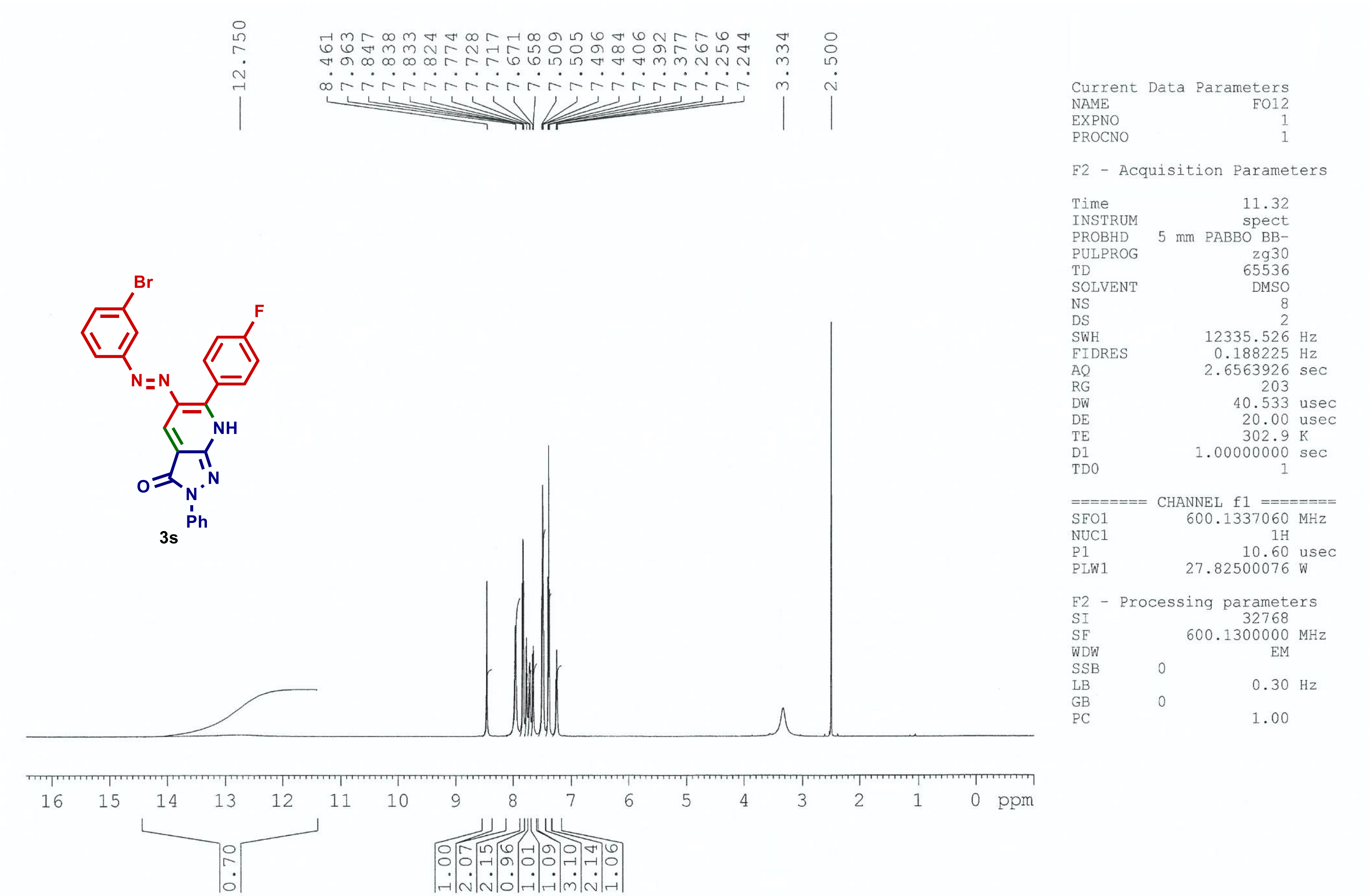

Figure S37. ^{1}H NMR Spectrum (DMSO-d_6, 600 MHz) for compound **3s**.

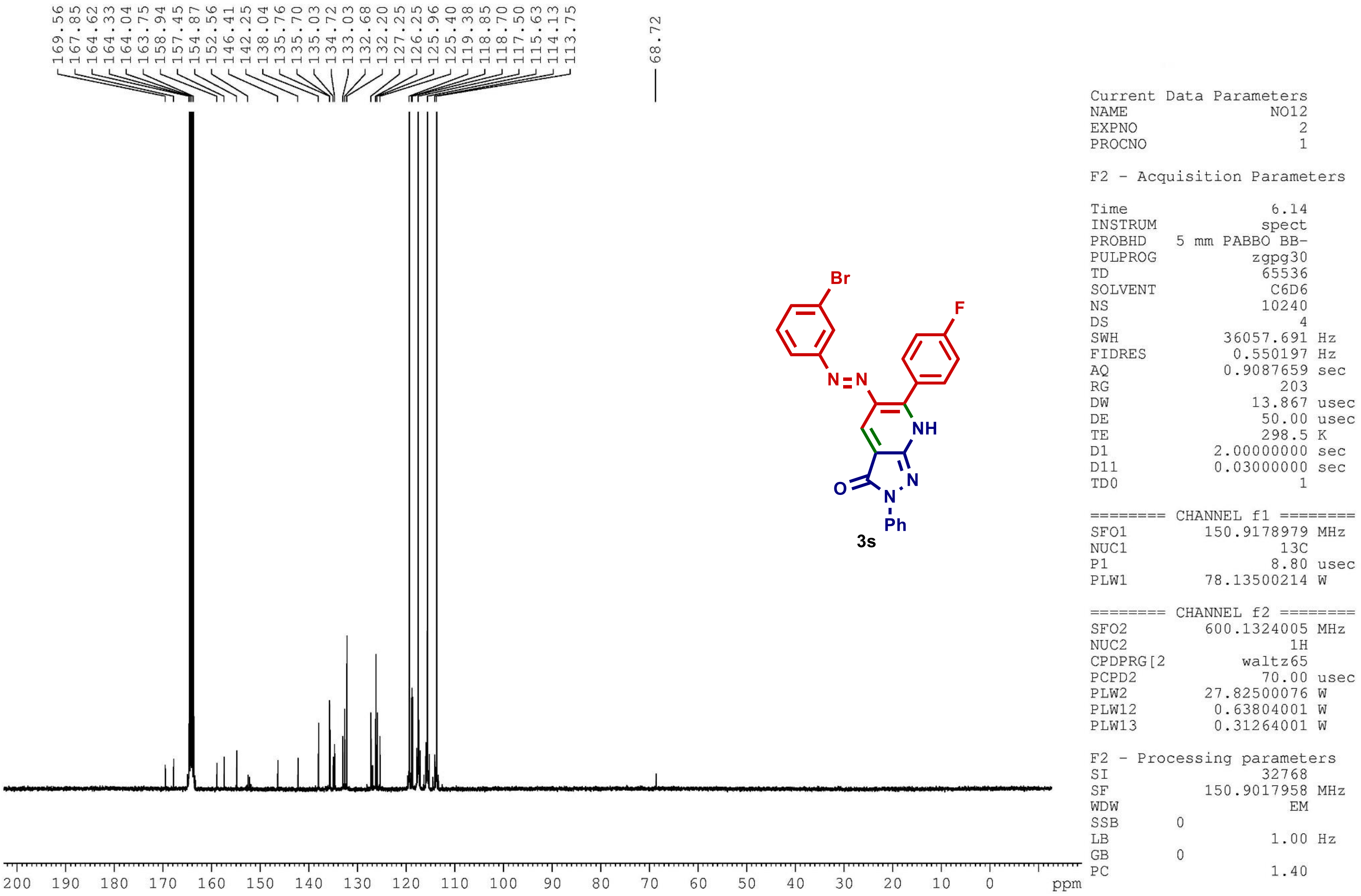

Figure S38. ^{13}C NMR Spectrum (TFA-*d*, 150 MHz) for compound **3s**

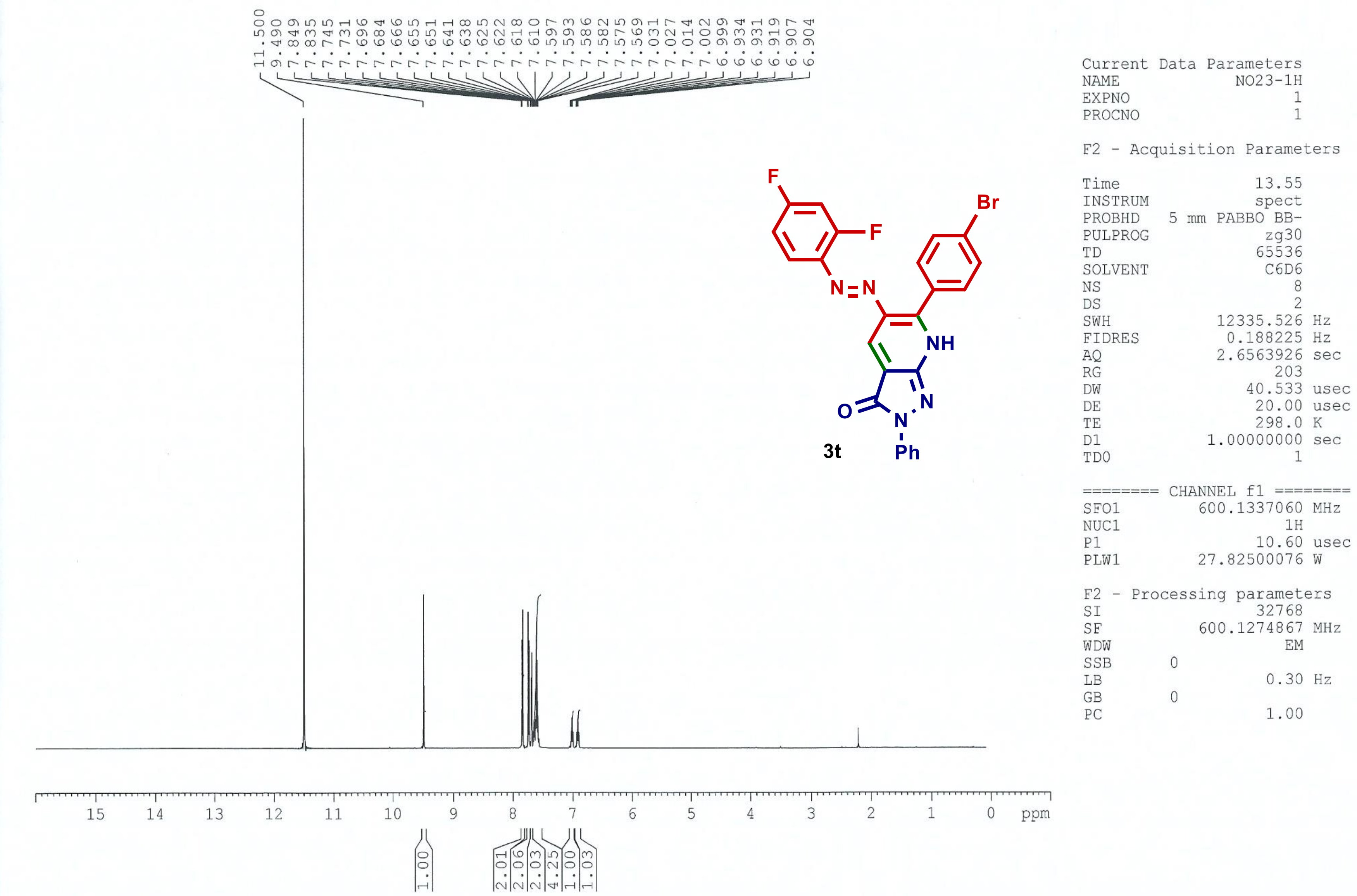

Figure S39. ^{1}H NMR Spectrum (TFA-*d*, 600 MHz) for compound **3t**.

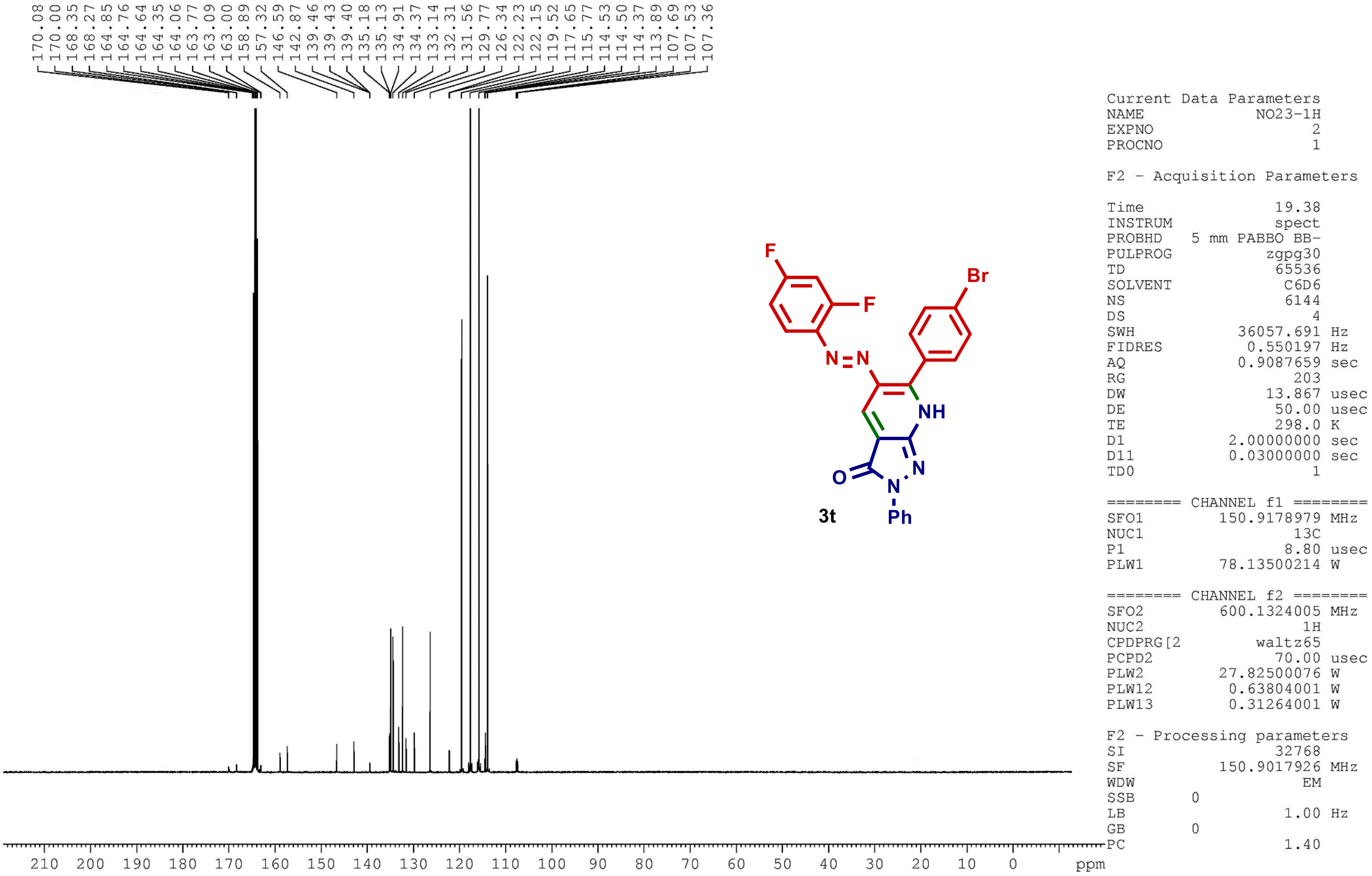

Figure S40. ^{13}C NMR Spectrum (TFA-*d*, 150 MHz) for compound **3t**.

.

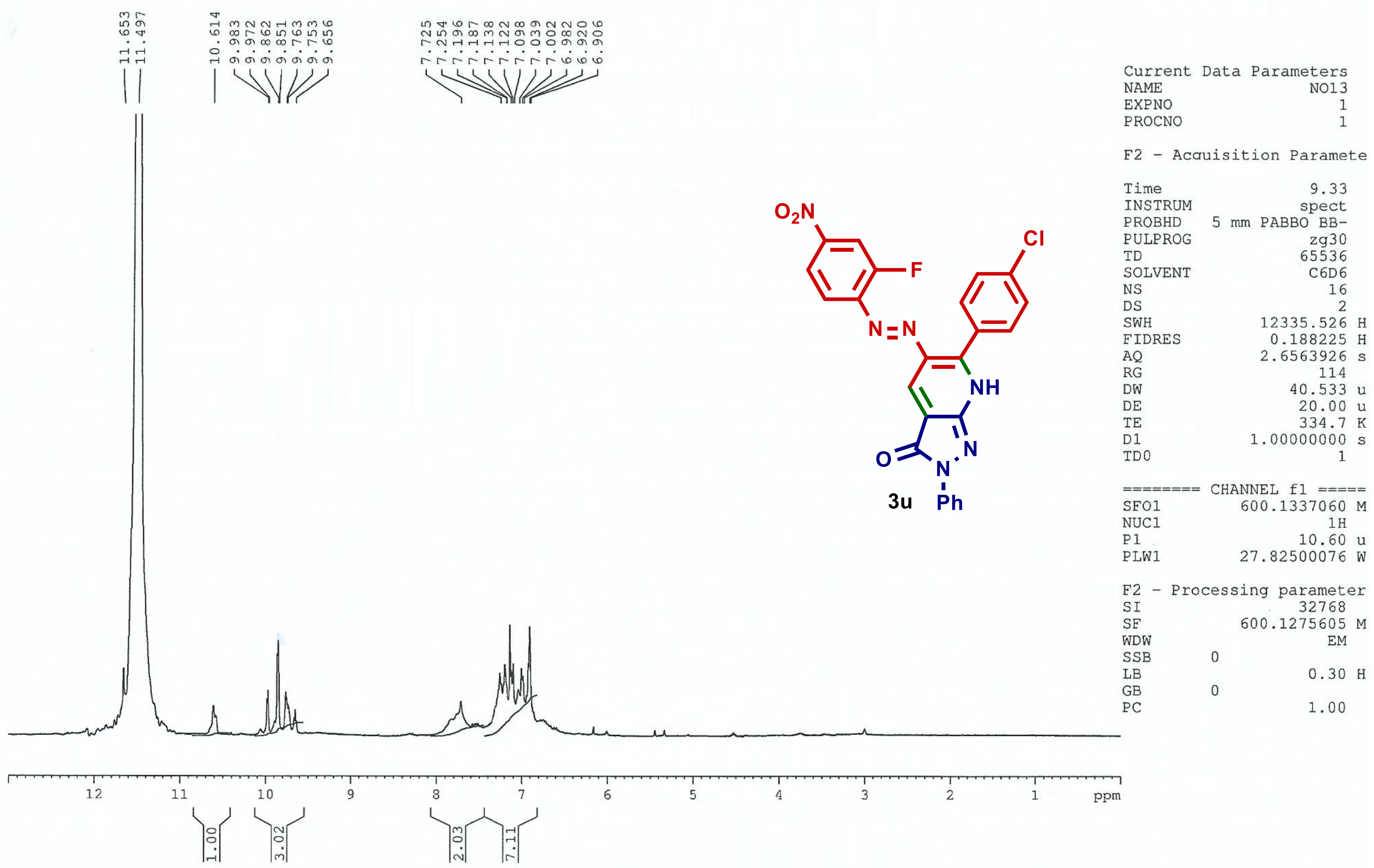

Figure S41. ^{1}H NMR Spectrum (TFA-*d*, 600 MHz) for compound **3u**.

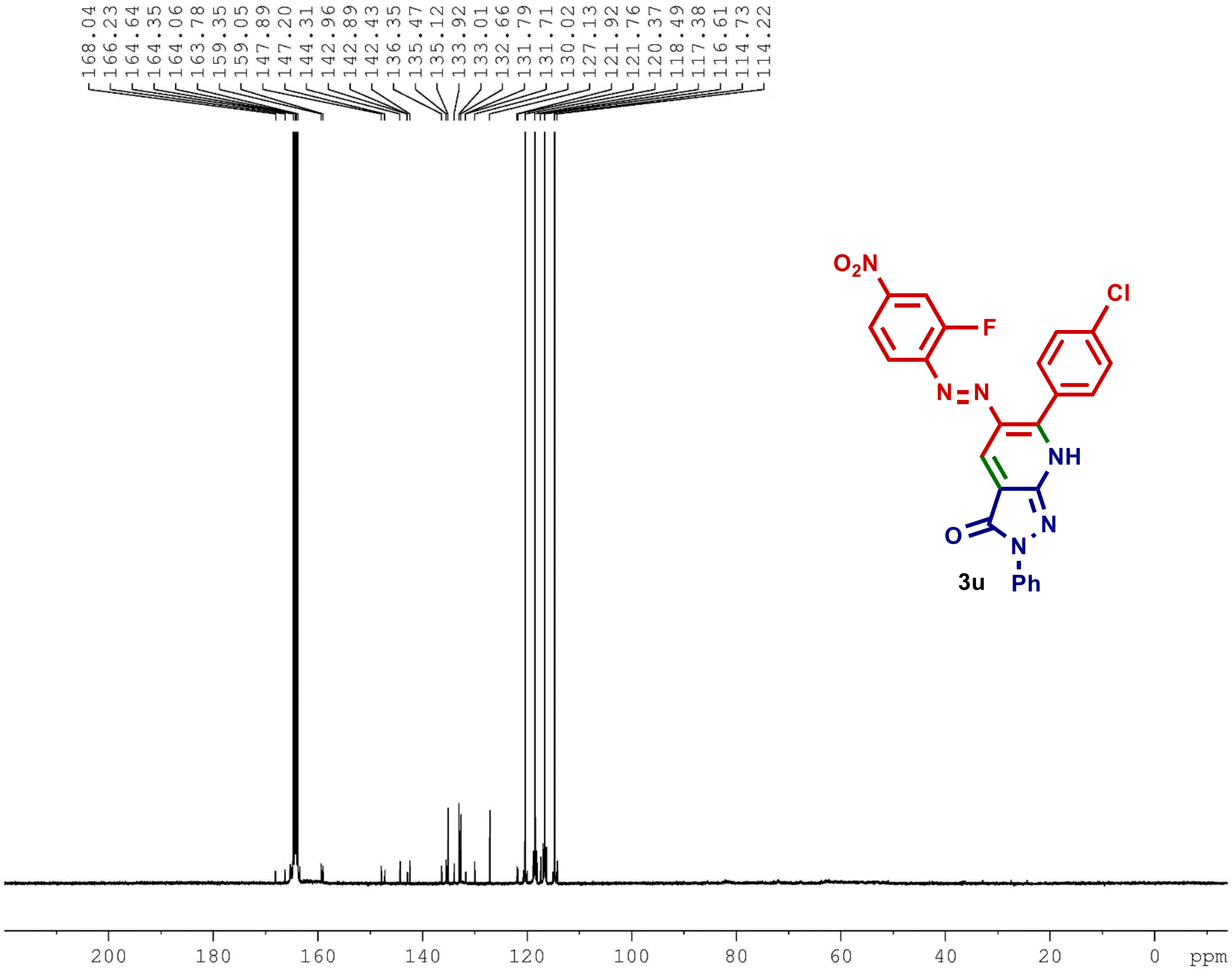

Current Data Parameters
NAME NO13-1H
EXPNO 1
PROCNO 1

F2 - Acquisition Parameters

Time 14.03
INSTRUM spect
PROBHD 5 mm PABBO BB-
PULPROG zgpg30
TD 65536
SOLVENT C6D6
NS 22528
DS 4
SWH 36057.691 Hz
FIDRES 0.550197 Hz
AQ 0.9087659 sec
RG 203
DW 13.867 usec
DE 50.00 usec
TE 298.4 K
D1 2.00000000 sec
D11 0.03000000 sec
TD0 1

======== CHANNEL f1 ========
SFO1 150.9178979 MHz
NUC1 13C
P1 8.80 usec
PLW1 78.13500214 W

======== CHANNEL f2 ========
SFO2 600.1324005 MHz
NUC2 1H
CPDPRG[2 waltz65
PCPD2 70.00 usec
PLW2 27.82500076 W
PLW12 0.63804001 W
PLW13 0.31264001 W

F2 - Processing parameters
SI 32768
SF 150.9019651 MHz
WDW EM
SSB 0
LB 1.00 Hz
GB 0
PC 1.40

Figure S42. ^{13}C NMR Spectrum (TFA-*d*, 150 MHz) for compound **3u**.

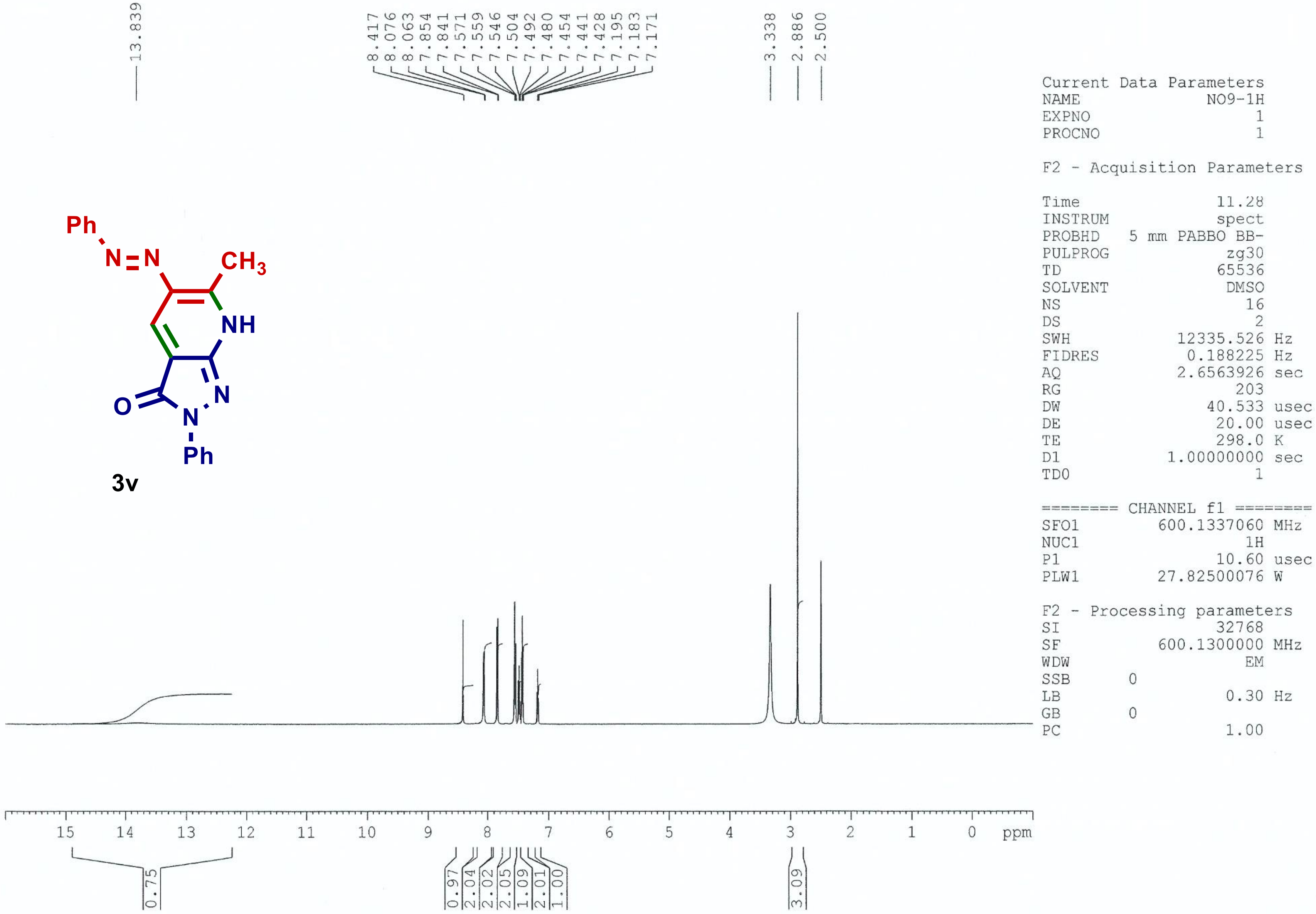

Figure S43. ^{1}H NMR Spectrum (DMSO-*d6*, 600 MHz) for compound **3v**.

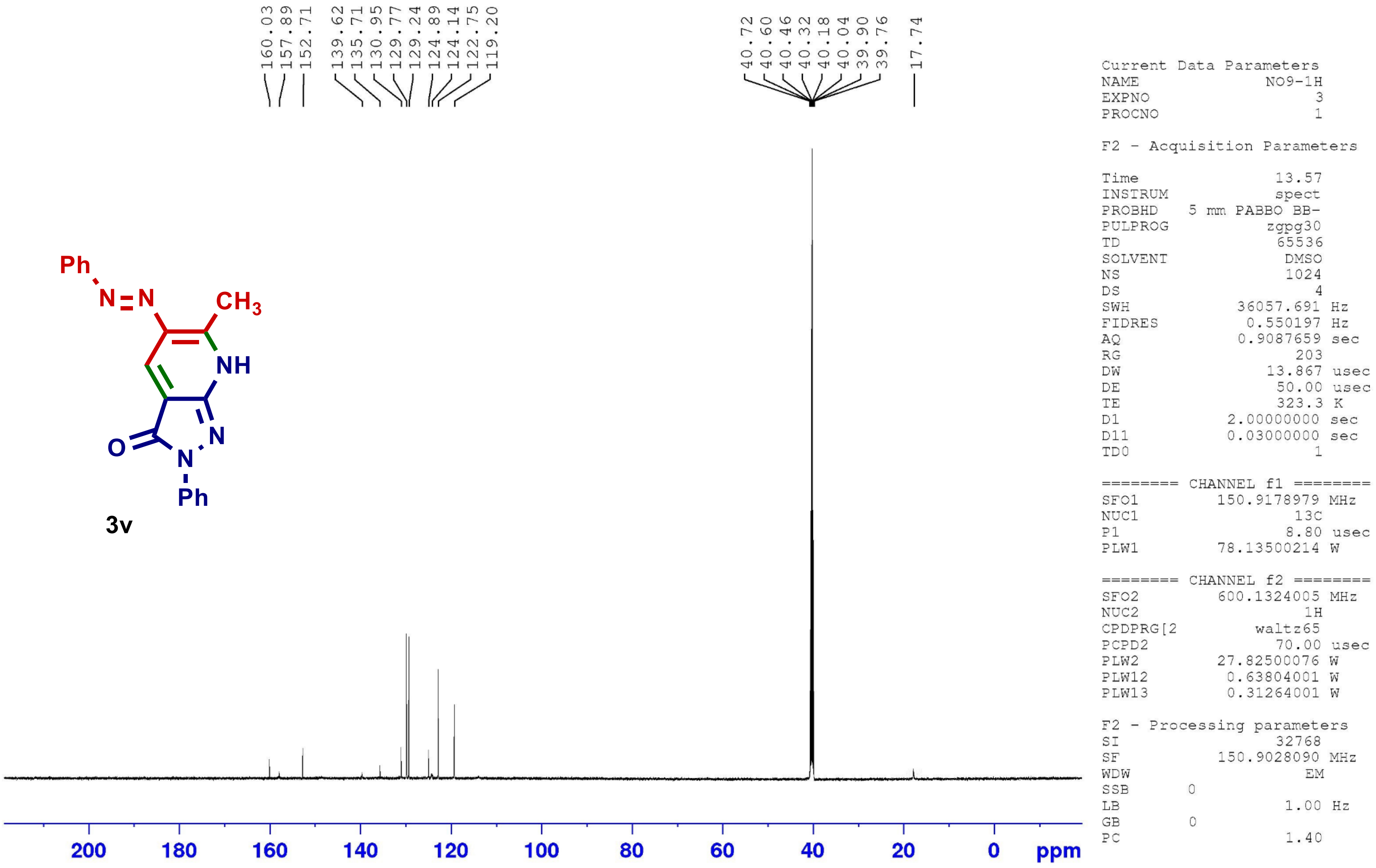

3v

Figure S44. ^{13}C NMR Spectrum (DMSO-*d6*, 150 MHz) for compound **3v**.
